# New Dihalogenated Derivatives of Condensed Benzimidazole Diones Promotes Cancer Cell Death Through Regulating STAT3/HK2 Axis/Pathway

**DOI:** 10.3390/molecules30214150

**Published:** 2025-10-22

**Authors:** Yulia Aleksandrova, Luiza Savina, Inna Shagina, Anna Lyubina, Alla Zubishina, Svetlana Makarova, Anna Bagylly, Alexander Khokhlov, Roman Begunov, Margarita Neganova

**Affiliations:** 1 Institute of Pharmacy, Yaroslavl State Medical University of the Ministry of Health of the Russian Federation, Yaroslavl 150000, Russia; yulia.aleks.97@mail.ru (Y.A.);; 2 Nesmeyanov Institute of Organoelement Compounds, Russian Academy of Sciences, Moscow 119991, Russia; 3 Arbuzov Institute of Organic and Physical Chemistry, FRC Kazan Scientific Center, Russian Academy of Sciences, Kazan 420088, Russia

**Keywords:** reductive intramolecular heterocyclization of ortho-nitro-tert-anilines, regioselectivity of the SEAr reaction, reduction of nitrohetarenes, oxidation of aromatic amines, condensed benzimidazole diones, antitumor agents, cytotoxic profile, in silico screening, STAT3, apoptosis, glycolysis

## Abstract

An effective method for synthesizing dihalogenated derivatives of condensed benzimidazole diones with a nodal nitrogen atom has been developed. As a result, five new heterocyclic quinones were obtained, which differed in the structure of the heterocycle annelated to imidazole, as well as the nature and arrangement of halogen atoms. A comprehensive analysis of the anticancer potential of new heterocyclic quinones revealed pronounced cytotoxic activity of the molecules against tumor cells. Using in silico methods for predicting activity spectra, it was found that the synthesized compounds are capable of interacting with a number of key targets that play an important role in oncogenesis, with the highest probability of binding to STAT3, the central regulator of cell growth, proliferation and metabolism. Experimental studies have shown that, despite the lack of pronounced ability to induce apoptosis, these substances effectively inhibit the activity of allosteric glycolytic enzymes, disrupting metabolic adaptation and energy balance of tumor cells. The obtained results expand the understanding of the molecular basis of the antitumor action of heterocyclic compounds and lay a solid foundation for their use as promising modulators of tumor cell metabolism.

## 1. Introduction

In recent years, the problem of effective therapy for malignant neoplasms has remained one of the most pressing challenges in modern medicine and pharmacology [1,2,3]. Progress in these fields is accompanied not only by the development of new drugs but also by a deeper understanding of the molecular mechanisms underlying their action. This enables the formation of personalized approaches to cancer treatment and the minimization of side effects associated with standard therapies. Despite advances in chemotherapy, immunotherapy, and targeted drugs, tumor cell resistance to known agents [4,5] and the pronounced toxicity of these drugs to healthy microenvironments [6,7] drive the scientific community to search for new molecules.

Molecules containing quinone functional groups are of particular interest [8]. The literature is rich with information showing that quinone compounds exhibit a wide spectrum of biological activities, including antibacterial [9,10,11,12], antifungal [13,14], antiviral [15,16], and primarily antitumor [11] properties. Quinones participate in electron transfer processes [17,18], modulate the redox status of cells [13], induce apoptosis [19,20], and can affect key signaling pathways responsible for metabolism proliferation, and survival [21] of tumor cells.

One notable example of a drug containing a quinone functional group is mitomycin C [22]. Although this molecule also includes other structural fragments such as aziridine and urethane, it has been shown that the quinone moiety is responsible for the formation of alkylating radicals that underlie its antitumor activity. Today, mitomycin C is clinically used as an antitumor agent for treating various cancers, including stomach cancer [23], pancreatic cancer [24], breast cancer [25], cervical cancer [26], bladder cancer [27,28], lung cancer (including non-small cell lung cancer) [29,30], colorectal cancer [31,32]. Nevertheless, the high reactivity of mitomycin C often leads to systemic toxicity [33] and the development of tumor cell resistance [34], which limits its broader practical application.

A promising approach to modifying the structure of mitomycin C is the synthesis of benzimidazole analogs [35], which feature a unique heterocyclic core capable of forming additional chemical interactions with biological targets in tumor cells [36,37].

**Figure 1:**
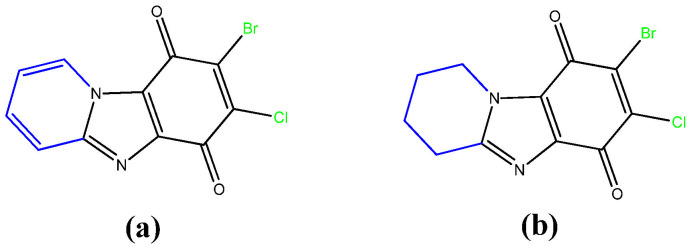
Structures of condensed benzimidazoldione derivatives with antitumor activity: (**a**)—8-bromo-7-chloropyrido[1,2-*a*]benzimidazole-6,9-dione and (**b**)—8-bromo-7-chloro-1,2,3,4-tetrahydropyrido[1,2-*a*]benzimidazole-6,9-dione.

Previously, we synthesized two new cytotoxic agents of a similar structure, 7,8-dihalogenpyrido[1,2-*a*]benzimidazole-6,8-dione (**a**) and 7,8-dihalogen-1,2,3,4-tetrahydropyrido[1,2-*a*]benzimidazole-6,8-dione (**b**) (Figure 1), and studied their cytotoxicity in in relation to cell lines of tumor and normal origin [38].

These substances had a high cytotoxic effect on all the studied tumor cell lines. The cytotoxic effect of the synthesized substances was comparable or several times higher than that of the commercial antitumor drugs Tamoxifen and Mitomycin C. Compound **b** with a saturated heterocycle was more promising than the unsaturated analogue **a**.

**Figure 2:**
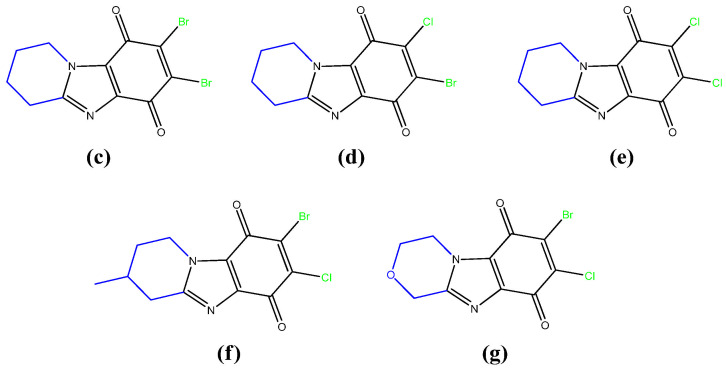
Structures of new condensed benzimidazoldione derivatives with antitumor activity: (**c**)—7,8-dibromo-1,2,3,4-tetrahydropyrido[1,2-*a*]benzimidazole-6,9-dione, (**d**)—7-bromo-8-chloro-1,2,3,4-tetrahydropyrido[1,2-*a*]benzimidazole-6,9-dione, (**e**)—7,8-dichloro-1,2,3,4-tetrahydropyrido[1,2-*a*]benzimidazole-6,9-dione, (**f**)—8-bromo-7-chloro-3-methyl-1,2,3,4-tetrahydropyrido[1,2-*a*]benzimidazole-6,9-dione, (**g**)—7-bromo-8-chloro-3,4-dihydro-1H-[1,4]oxazino[4,3-a]benzimidazole-6,9-dione.

In continuation of these studies, a number of these promising substances have been expanded in this work. For this purpose, an effective method of their synthesis has been developed. As a result, 5 new condensed benzimidazolediones with a nodal nitrogen atom were obtained (compounds **c**–**g** Figure 2), which differed in the structure of the heterocycle annelated to imidazole, the nature and arrangement of the halogen atoms.

To study the effect of the quinone structure on antitumor activity, the previously described compound **b** was also obtained.

In this work, we focused on studying the cytotoxic effects of new benzimidazole analogs of mitomycin C—condensed benzimidazolidiones with a bridging nitrogen atom—and investigating the molecular mechanisms underlying their antitumor activity. Of particular interest were the effects of these compounds on the regulation of key signaling pathways responsible for the survival of cancer cells.

It should be noted that studies like these enable the identification of rational directions for the design of new, more promising compounds for further pharmacological development and clinical application.

Thus, investigating the antitumor potential of new condensed benzimidazolidiones is a relevant and important task capable of making a significant contribution to the development of anticancer agents with improved selectivity for cancer cells and reduced side effects on healthy microenvironments.

## 2. Results and Discussion

### 2.1. Chemistry

**Scheme 1:**
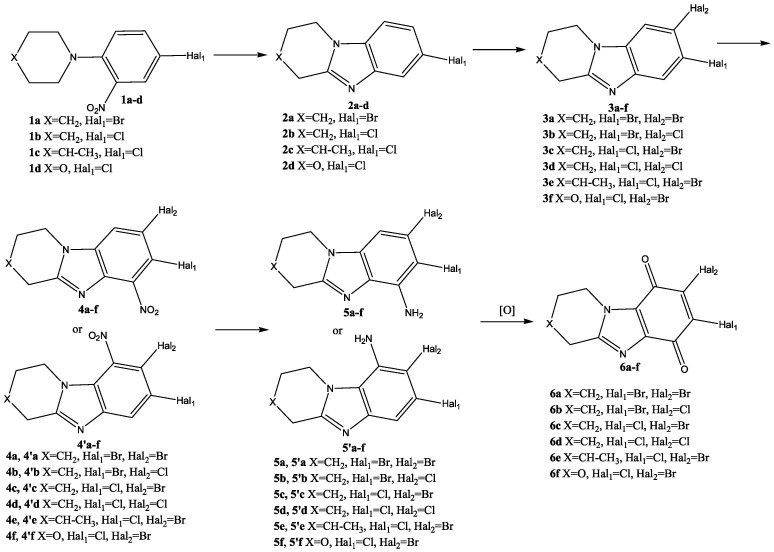
The proposed scheme for the synthesis of condensed dihalogen derivatives of benzimidazolediones **6**.

The synthesis of condensed dihalogen-substituted benzimidazoldiones **6** was planned to be carried out according to Scheme 1.

**Scheme 2:**
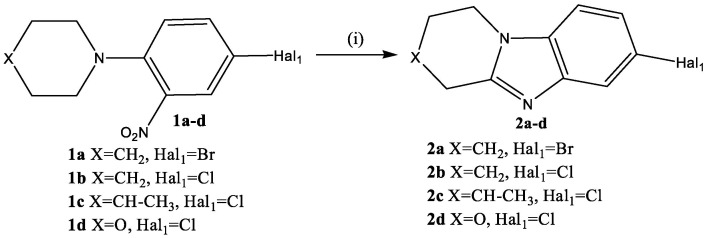
Synthesis of condensed benzimidazoles with a nodal nitrogen atom **2a**–**d**. Reagents and conditions: (i) SnCl_2_∙2H_2_O, 8% HCl, 80 °C, reducing agent application time 2 h.

At the first stage, a condensed heterocyclic core of benzimidazole **2** was formed in the reaction of reducing intramolecular cyclization of *ortho*-nitro-*tert*-anilines (**1a**–**d**) (Scheme 2).

This synthesis method is widely used to produce various condensed benzimidazole derivatives [39,40,41,42,43,44,45,46,47].

It turned out that the nature of the reducing agent and the time of its application, the concentration of HCl in the reaction mixture and the reaction temperature had a strong influence on the course of this process. The highest yield of cyclization product **2** (90–94%) was observed when using a solution of SnCl_2_·2H_2_O in 8% HCl, which was gradually added to the solution of nitro compound **1** in 8% HCl at 80 °C for 2 h.

**Figure 3:**
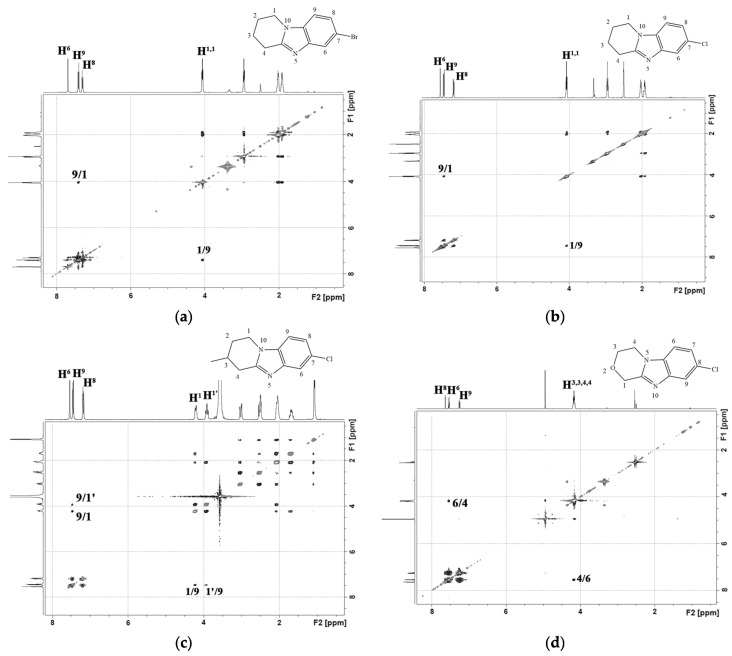
^1^H-^1^H NOESY NMR spectra of (**a**) 7-bromo-1,2,3,4-tetrahydropyrido[1,2-*a*]benzimidazole (**2a**), (**b**) 7-chloro-1,2,3,4-tetrahydropyrido[1,2-*a*]benzimidazole (**2b**), (**c**) 7-chloro-3-methyl-1,2,3,4-tetrahydropyrido[1,2-*a*]benzimidazole (**2c**) and (**d**) 8-chloro-3,4-dihydro-1H-[1,4]oxazino[4,3-*a*]benzimidazole (**2d**).

In the ^1^H-^1^H NMR spectra of the obtained condensed heterocycles **2**, a cross peak of the aromatic proton of the H^9(6)^ benzene ring with protons of the H^1,1(4,4)^ methylene group of the saturated heterocycle was observed (Figure 3).

Based on the presence of this cross peak in the ^1^H-^1^H NMR spectra, the composition of the reaction mixture products formed during the functionalization of condensed benzimidazoles **2** in the S*_E_*Ar reaction was subsequently analyzed.

**Scheme 3:**
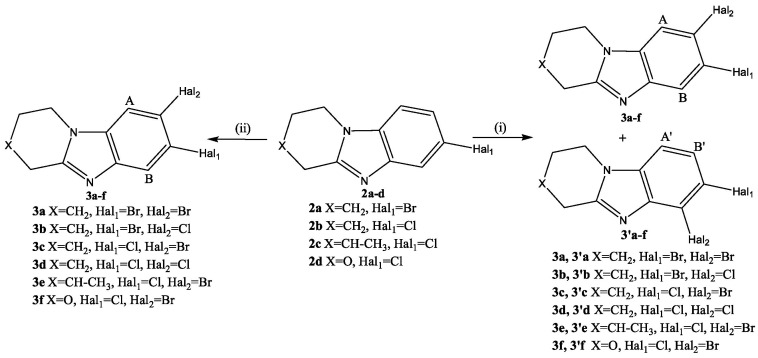
The halogenation reaction of condensed benzimidazoles with a nodal nitrogen atom **2a**–**d**. Reagents and conditions: (i) NBS or NCS, H_2_SO_4_, 35–40 °C, halogenating agent application time 0.5 min, reaction time 4 h; (ii) NBS or NCS, H_2_SO_4_, 35–40 °C, halogenating agent application time 4 h.

The halogenation reaction of condensed benzimidazole **2** was carried out in concentrated sulfuric acid with bromine or chlorosuccinimide at 35–40 °C. With the rapid introduction of a halogenating agent, two isomeric products **3** and **3′** were formed (Scheme 3(i)).

In the ^1^H NMR spectrum of the reaction mixture, signals of 2 aromatic protons (A, B), having the form of a singlet, of substance **3**, and signals of 2 protons (A′, B′) in the form of doublets (*J* = 8.4–8.7 Hz) of another isomer **3**′ were present. The signals of protons having the form of singlets belonged to the product of introducing a substituent into the 8th (7th) position of the condensed heterocycle **2**. This isomer **3** was formed in larger quantities.

**Figure 4:**
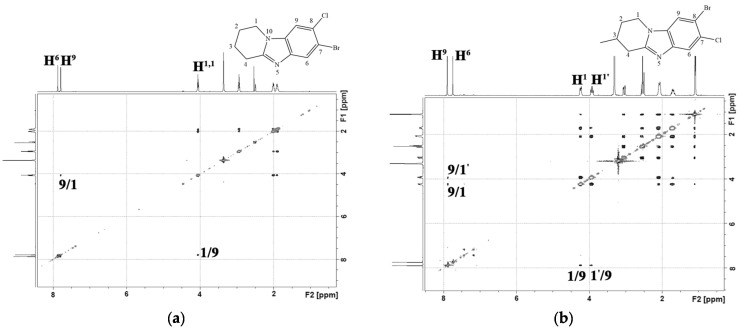
^1^H-^1^H NOESY NMR spectra of (**a**) 7-bromo-8-chloro-1,2,3,4-tetrahydropyrido[1,2-*a*]benzimidazole (**3b**) and (**b**) 8-bromo-7-chloro-3-methyl-1,2,3,4-tetrahydropyrido[1,2-*a*]benzimidazole (**3e**).

The gradual addition of a halosuccinimide solution over 4 h increased the selectivity of the reaction (Scheme 3(ii)). The reaction mixture contained trace amounts of product **3**′. After recrystallization in methanol, the yield of **3a**–**f** dihalide derivatives was 89–93%. Figure 4 shows the ^1^H-^1^H NMR spectra of 7-bromo-8-chloro-1,2,3,4-tetrahydropyrido[1,2-*a*]benzimidazole (**3b**) and 8-bromo-7-chloro-3-methyl-1,2,3,4-tetrahydropyrido[1,2-*a*]benzimidazole (**3e**). In the 2D spectra of these compounds, as well as in the spectra of the starting materials **2b** and **2e**, a cross peak of the aromatic proton H^9^ of the benzene ring with protons of the H^1,1^ methylene group of the saturated heterocycle was observed.

**Scheme 4:**
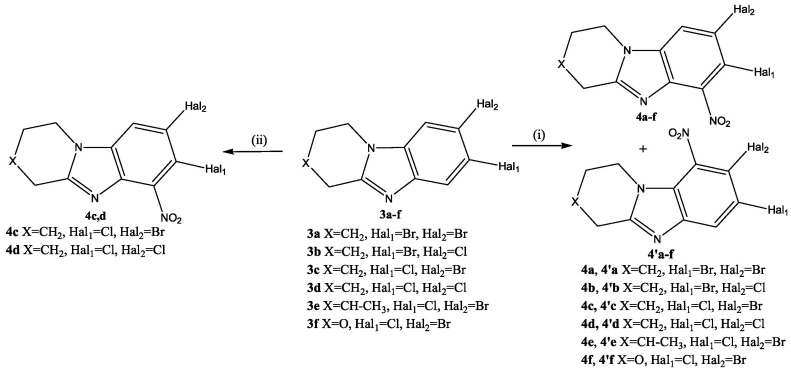
The nitration reaction of halogen derivatives of condensed benzimidazoles with a **3a**–**d** nodal nitrogen atom. Reagents and conditions: (i) KNO_3_, H_2_SO_4_, 30 °C, nitrating agent application time 0.5 min, reaction time 1 h; (ii) KNO_3_, H_2_SO_4_, 30 °C, nitrating agent application time 2 h.

**Scheme 5:**
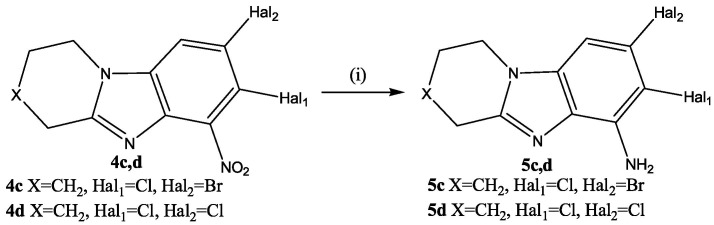
Synthesis of amino compounds **5c**,**d** in the reduction reaction. Reagents and conditions: (i) TiCl_3_, 10% HCl, *i*-PrOH, 60 °C, 5 min.

**Scheme 6:**
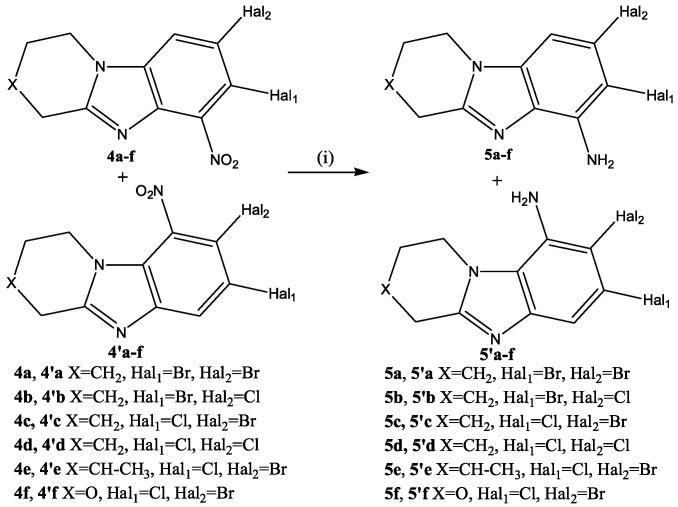
Joint synthesis of condensed benzimidazole-6-amines (**5**) and benzimidazole-9-amines (**5′**). Reagents and conditions: (i) TiCl_3_, 10% HCl, *i*-PrOH, 60 °C, 5 min.

**Scheme 7:**
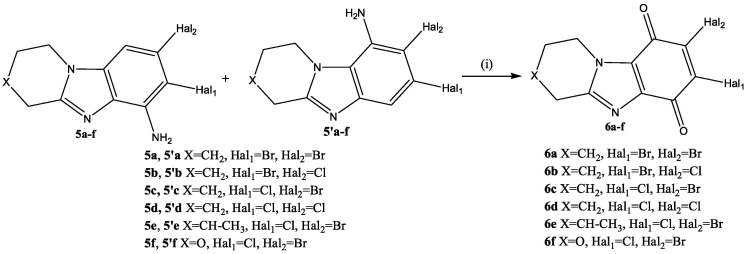
Oxidation reaction of condensed benzimidazolamine **5** or mixtures of isomeric amino compounds **5** and **5′**. Reagents and conditions: (i) KNO_3_, H_2_SO_4_, 20 °C, 8 h.

It is known that the presence of an amino group facilitates the oxidation of the benzene ring [48,49]. Therefore, subsequently, amino derivatives **5** (Scheme 4, Scheme 5 and Scheme 6) were obtained from benzannelated heterocycles **3**, which were oxidized to the corresponding condensed benzimidazoldiones **6** (Scheme 7).

Low selectivity of the S*_E_*Ar reaction was observed during nitration of condensed heterocycles **3** with a mixture of KNO_3_/H_2_SO_4_. Upon rapid mixing of solutions of substrate **3** and KNO_3_ in sulfuric acid at 30 °C, a mixture of **4a**–**f** and **4**′**a**–**f** products was formed in a ratio of 1: 0.18–0.24 (Scheme 4). The total yield of nitro derivatives **4** and **4**′ was 92–96%.

**Figure 5:**
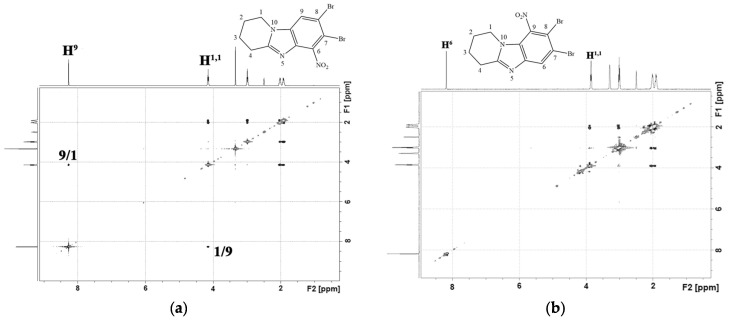
^1^H-^1^H NOESY NMR spectra of (**a**) 7,8-dibromo-6-nitro-1,2,3,4-tetrahydropyrido[1,2-*a*]benzimidazole (**4a**) and (**b**) 7,8-dibromo-9-nitro-1,2,3,4-tetrahydropyrido[1,2-*a*]benzimidazole (**4′a**).

Products **4a** and **4**′**a** were isolated individually from the reaction mixture of the nitration reaction of 7,8-dibromo-1,2,3,4-tetrahydropyrido[1,2-*a*]benzimidazole (**3a**). In the ^1^H-^1^H NMR spectrum of isomer **4a** (Figure 5a), there was a cross peak of the interaction of the aromatic proton H^9^ with protons of the H^1,1^ methylene group of the piperidine cycle. This cross peak was absent in the 2D spectrum of the isomeric product **4**′**a** (Figure 5b). This indicated the presence of a substituent in the 9th position of the **4**′**a** heterocycle.

**Figure 6:**
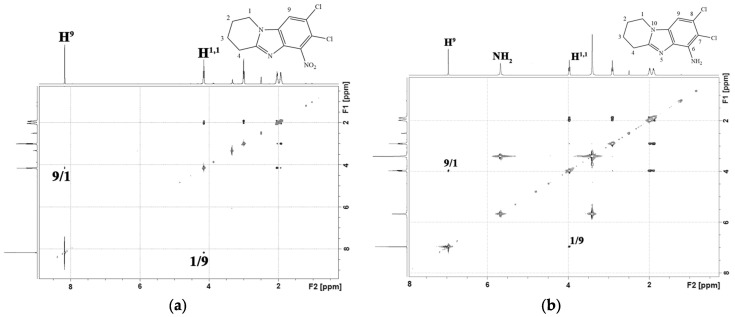
^1^H-^1^H NOESY NMR spectra of (**a**) 7,8-dicloro-6-nitro-1,2,3,4-tetrahydropyrido[1,2-*a*]benzimidazole (**4d**) (DMSO-*d*6) and of (**b**) 7,8-dicloro-1,2,3,4-tetrahydropyrido[1,2-*a*]benzimidazole-6-amine (**5d**).

The slow application of a nitrating agent for 2 h, as in the halogenation reaction, resulted in the predominant formation of only one product. Thus, after recrystallization in methanol, 8-bromo-7-chloro-6-nitro-1,2,3,4-tetrahydropyrido[1,2-*a*]benzimidazole (**4c**) and 7,8-dicloro-6-nitro-1,2,3,4-tetrahydropyrido[1,2-*a*]benzimidazole (**4d**) were obtained in yields of 91% and 89%. In the 2D spectrum of the compound (**4d**) (Figure 6a), there was only one signal of the aromatic proton H^9^, which had a cross peak with aliphatic H^1,1^.

A 15% solution of TiCl_3_ in 10% HCl was used to restore the **4c**,**d** nitro compounds (Scheme 5). Its use made it possible to quickly obtain the corresponding amino compounds **5c**,**d** with yields of 95% and 94%. No formation of by-products was observed.

An H^9^/H^1^ cross peak was present in the 2D NMR spectra 7,8-dicloro-1,2,3,4-tetrahydropyrido[1,2-*a*]benzimidazole-6-amine (**5d**) (Figure 6b). There was no cross-peak of protons of the amino group with other protons.

It should be noted that the target benzimidazoledione **6** can be obtained by oxidation of both condensed benzimidazole-6-amine **5** and isomeric benzimidazole-9-amine **5′**. Therefore, in order to simplify the synthesis of condensed benzimidazoldiones **6**, the preparation of some nitro- and amino compounds in an individual form was not carried out. The resulting mixture of nitro compounds **4** and **4′** was reduced with titanium (III) chloride under the conditions described above. The total yield of mixtures of amino derivatives **5** and **5′** was high 93–97% (Scheme 6).

The resulting mixture of nitro compounds **4** and **4′** was reduced with titanium (III) chloride under the conditions described above. The total yield of mixtures of amino derivatives **5** and **5′** was high 93–97%.

For the oxidation of individual amino compounds **5** or mixtures of amino products **5** and **5′**, the KNO_3_/H_2_SO_4_ system (Scheme 7) was used, the effectiveness of which was demonstrated by us in the work [50].

The reaction was carried out at room temperature for 8 h. The yield of condensed benzimidazolediones was 88–92%. It should be noted that the yield of quinones **6** obtained by oxidation of individual amines **5** or mixtures of isomers **5** and **5′** was approximately the same.

**Figure 7:**
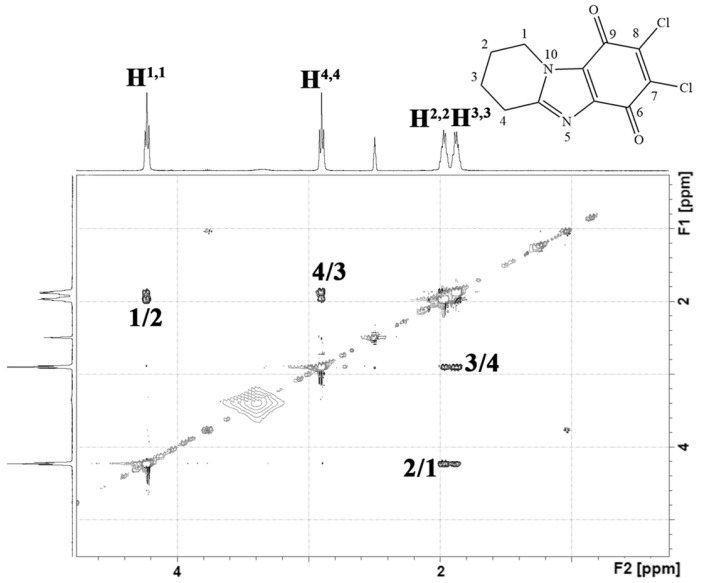
^1^H-^1^H NOESY NMR spectra of 7,8-dichloro-1,2,3,4-tetrahydropyrido[1,2-*a*]benzimidazole-6,9-dione (**6d**) (DMSO-*d*6).

In the 2D NMR spectrum of the obtained quinone **6d** (Figure 7), only cross peaks of aliphatic protons of the piperidine cycle were observed. There were no aromatic proton signals. Therefore, it can be argued that the oxidation reaction of the benzene ring containing the amino group was successful.

Thus, an effective method for the synthesis of condensed benzimidazolediones **6** was developed, which allowed us to obtain a number of new potential antitumor substances of various structures.

Subsequently, the obtained benzimidazolediones **6** were used to search for new cytostatic agents against cell lines of tumor origin.

### 2.2. In Vitro Study and In Silico Prediction of the Cytotoxic Profile of New Heterocyclic Quinones ***6a***–***6f***

In the first stage of this study, we investigated the cytotoxic properties of a series of new heterocyclic quinones. The known antitumor cytostatic agent mitomycin, which possesses a heterocyclic structure, was used as a reference molecule to validate our model system [51]. The cytotoxic effects of the synthesized compounds were tested on the cell lines A549, MCF-7, SW-480, SH-SY5Y, and HEK-293. The selection of these cell lines was deliberate. Their different origins (tumor and normal) and phenotypic features became possible to identify patterns in the selectivity of the cytotoxic action of the molecules.

As shown in Table 1, the IC_50_ values of the cytotoxic effect for the reference molecule mitomycin ranged from 8 µM to 20 µM, with the most pronounced activity observed in the SH-SY5Y and HEK-293 cell lines. These results correspond well to previously published data in relevant sources [52,53,54]. For example, IC_50_ values for the cytotoxic effect on human breast adenocarcinoma MCF-7 cells range from 16 to 35 µM at comparable exposure times [52,55]. Similar correlations were found between our data and published results for other cell lines.

During the cytotoxicity assessment of cells treated with serial dilutions (0.01, 0.1, 1, 3, 10, 30, 100 µM) of the synthesized compounds **6a**–**6f** for 24 h, a significant decrease in cell viability was observed for all compounds. The calculated IC_50_ values indicated a range from moderate (>15 µM) to high (<15 µM) cytotoxic activity.

Importantly, the IC_50_ values for most cell lines were more promising compared to the reference molecule. The synthesized compounds showed similar efficacy across all cell lines; therefore, no selectivity towards a specific cell phenotype was detected. However, a noteworthy finding is the significantly reduced cytotoxic effect of **6a**–**6f** on normal-origin HEK-293 cells. According to the data in Table 1, the IC_50_ values for human embryonic kidney cells increased by more than twofold. This effect is undoubtedly a favorable property of the molecules and can be considered a step toward developing selective compounds that target the tumor microenvironment specifically.

It is also important to note that mitomycin showed equivalent or, in some cases, more pronounced cytotoxicity toward normal HEK-293 cells. This aligns with strong literature evidence regarding the nonspecific action of mitomycin and the resulting diverse side effects observed during therapy with this cytostatic agent. For instance, intravesical administration of mitomycin C in patients with non-muscle invasive bladder cancer can cause non-allergic contact dermatitis [56]. Other dermatological side effects were reported by Cicek et al., where 10 days after receiving mitomycin treatment for metastatic breast carcinoma, a patient developed a psoriasiform drug rash [57]. All these facts explain the significant focus of many research groups on addressing the problem of reducing mitomycin’s side effects [58,59].

Additionally, to expand the understanding of the cytotoxic profile of the synthesized molecules, an in silico analysis of their cytotoxic potential was performed using the software CLC-Pred 2.0 (Cell Line Cytotoxicity Predictor, S25–S26 Supplementary Materials), which is a validated tool for in silico assessment of cytotoxic properties [60]. The calculated data are presented as probabilities of activity (P_a_) and inactivity (P_i_).

The CLC-Pred analysis revealed that at a threshold P_a_ value greater than 0.5, indicating a probable manifestation of activity under experimental conditions, most of the synthesized molecules (**6a**–**6d**) are highly likely to inhibit the viability of tumor cells of various origins. Notably, among the cancer cells predicted to be affected are lung and colon cancer cell lines, which correlates with our in vitro data from phenotypically similar A549 and SW-480 cell lines.

Interestingly, despite the seemingly similar structures of the synthesized molecules, we observed variation in the types of cells for which cytotoxic activity was predicted depending on substituents. In particular, the introduction of two chlorine atoms into the molecular structure significantly broadens the spectrum of expected effects, with a high probability of experimental validation. The potential of halogenating molecules with chlorine atoms to enhance antitumor activity is supported by numerous published studies [61,62,63].

Importantly, when setting the P_a_ threshold above 0.5, none of the studied molecules were predicted to exhibit cytotoxicity toward normal-origin cell lines. This correlates with experimental cytotoxicity profiles where IC_50_ values on human embryonic kidney HEK-293 cells for the synthesized compounds were higher compared to cancer cells.

Thus, the results obtained from the in silico prediction of the cytotoxic profile of the synthesized compounds not only confirm the data from in vitro experiments but also underscore the importance and necessity of conducting further in-depth studies. These studies will focus on a more detailed investigation and identification of the cancer types that are most sensitive and susceptible to the action of these compounds. This approach will help optimize the use of these compounds as potential therapeutic agents and enhance the effectiveness of their application.

### 2.3. In Silico and In Vitro Study of the Cytotoxic Mechanism of Heterocyclic Quinones ***6a***–***6f***

To elucidate the mechanism underlying the ability of the synthesized compounds to reduce viability of tumor cells, we conducted a combined in silico and in vitro investigation, which helped identify possible mechanisms of cytotoxic action of the heterocyclic quinones.

Initially, a molecular target analysis for all new compounds was performed using the PASS (Prediction of Activity Spectra for Substances) approach via the Way2Drug web server [64]—a widely used tool for predicting probable biological activities and mechanisms of novel compounds.

**Figure 8:**
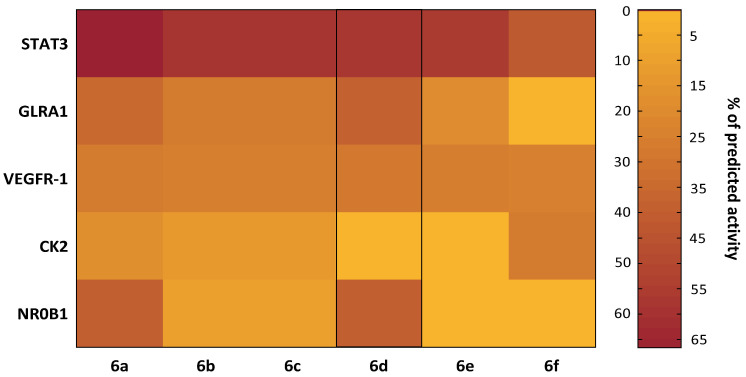
Heat map of the most probable targets involved in malignant tumor pathogenesis for heterocyclic quinones **6a**–**6f**. The smooth gradient transition from yellow to brown indicates an increase in predicted activity. NR0B1—nuclear receptor subfamily 0 group B member 1; CK2—casein kinase II; VEGFR-1—vascular endothelial growth factor receptor 1; GLRA1—glycine receptor subunit alpha-1.

As shown in the heatmap (Figure 8), the high antitumor potential of the studied heterocyclic quinones may be due to their probable action on several key targets involved in cancer pathogenesis.

A robust body of literature highlights the crucial role of nuclear receptor subfamily 0 group B member 1 (NR0B1) in cancer pathogenesis [65]. Notably, NR0B1 is associated with the development and intensification of drug resistance in tumor cells. For instance, a recent study by Tan et al. found NR0B1 promotes resistance progression in hepatocellular carcinoma cells treated with sorafenib by enhancing proliferation, migration, and invasion via apoptosis inhibition and autophagy activation. Another study by Zhang et al. showed NR0B1 contributes to increased therapeutic resistance in lung cancer cells through ferroptosis suppression via the c-JUN/NRF2-CBS signaling pathway [66].

Expression of casein kinase II (CK2) negatively correlates with overall and relapse-free survival in cancer patients, as many growth-related proteins are CK2 substrates, explaining its key role in tumor cell proliferation [67]. Similar to NR0B1, CK2 plays a critical role in enhancing resistance to anticancer therapy [68,69]. Currently, specific CK2 inhibitors are used clinically, and other therapeutic strategies targeting CK2 show promising results in treating various types of cancer [70,71].

Vascular endothelial growth factor receptor 1 (VEGFR-1), a cancer-associated receptor, is another attractive target for anticancer agents. VEGFR-1 expression correlates with invasiveness and recurrence in bladder [72], prostate [73], colorectal [74], and other cancers [75]. Another likely molecular marker and target of most synthesized compounds (except **6f**) is glycine receptor subunit alpha-1 (GLRA1). GLRA1 expression is normally limited to spinal cord, brainstem, and retina cells, but this regulation is disrupted in small cell lung cancer, facilitating apoptosis evasion and disease progression [76]. Targeting this protein is thus a promising antitumor strategy.

Of special interest are the predictions for the signal transducer and activator of transcription 3 (STAT3), which showed the highest modulation probability across all compounds. Given STAT3’s crucial regulatory role in determining cancer cell fate [77,78,79], there is intense interest in developing STAT3-targeted therapies.

In our study, all synthesized heterocyclic quinones demonstrated a high likelihood (40.70% to 66.59%) of affecting STAT3, with compound **6d** showing the highest predicted inhibitory value (66.59%). The distinctive feature of this molecule is the presence of two bromine atoms in its structure.

Notably, **6d** exhibited not only the broadest predicted activity spectrum among the aforementioned targets but also the highest experimental activity probability. This supports the promise of our chemical modification and aligns with literature showing the benefits of bromine incorporation into chemical compounds. For example, Sabarwal et al. demonstrated that brominated fisetin analogues are more effective antiproliferative agents both in vitro and in vivo [80] compared to the parent phytomolecule [81], partly by inducing cell cycle arrest and enhancing apoptosis via the STAT3 signaling pathway. Overall, there is abundant evidence supporting the high potential of halogenation in the design of anticancer agents [82,83].

It is well known that STAT3 is hyperactivated in tumor cells, contributing to therapy resistance by suppressing programmed cell death and supporting cell survival [84,85]. Hence, STAT3 plays a key role in apoptosis regulation [86]. STAT3 is activated through phosphorylation and functions as a transcription factor that upregulates genes encoding anti-apoptotic proteins, including Bcl-2 family members and apoptosis inhibitors [87,88]. These proteins in turn block caspase activation and other cell death mechanisms, suppressing apoptosis [86]. Additionally, STAT3 interacts with other signaling pathways such as NF-κB and PI3K/Akt, which participate in anti-apoptotic cell protection, rendering cells more resistant to stress and damage [89,90].

These facts motivated our investigation of the effects of heterocyclic quinones **6a**–**6f** on apoptosis by flow cytometry. Since none of the synthesized compounds showed selective activity toward a particular cell line, we chose the A549 lung adenocarcinoma cell line for this experiment. This line is widely used in such studies due to ease of cultivation, stability, reproducibility of results, and sensitivity to apoptosis-inducing stimuli.

**Figure 9:**
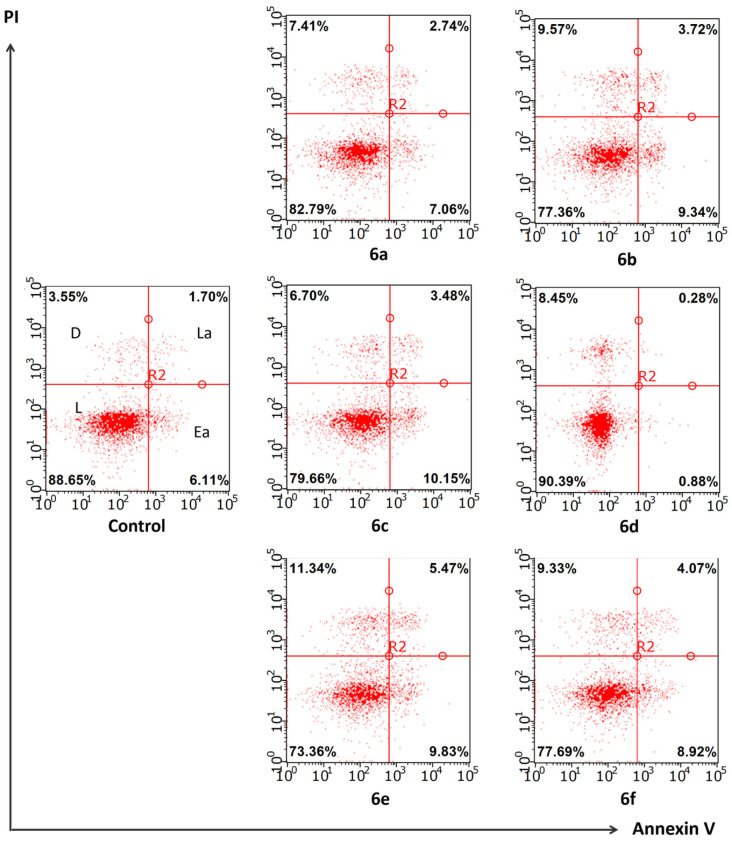
Apoptosis induction in A549 cells incubated with compounds heterocyclic quinones **6a**–**6f**. L—live cells; D—dead cells; Ea—cells in early apoptosis; La—cells in late apoptosis; and PI—propidium iodide. The dots represent events related to measuring the optical properties of cells, allowing individual cell populations to be distinguished.

As shown in Figure 9, none of the tested compounds induced significant apoptosis. This is evidenced by the fact that the proportions of live cells (lower left quadrant) and cells in early (lower right quadrant) or late (upper right quadrant) apoptosis stages in the experimental groups did not differ from those in the control group. Apoptosis values only slightly deviated from baseline levels, and statistical analysis revealed no significant differences in cell distribution across the quadrants between control and treated groups (*p* > 0.05).

Thus, the data indicate that the tested compounds do not possess apoptosis-inducing activity in the lung adenocarcinoma cell line, and the cytotoxicity of **6a**–**6f** is mediated by another mechanism of action.

Since no significant effect of the compounds on apoptosis was observed, we aimed to clarify the possible mechanism of action of the new heterocyclic quinones **6a**–**6f** through their interaction with STAT3.

Our literature review revealed a remarkable fact that STAT3 promotes cancer cell adaptation to hypoxic conditions [91,92]. This is apparently due to STAT3’s ability to enhance glucose metabolism, thereby intensifying glycolysis in cancer cells [93]. STAT3 enhances the regulation of glycolysis rate-limiting enzymes—hexokinase II (HK2), phosphofructokinase I (PFK1), and pyruvate kinase M2 (PKM2)—in various cancer types [94,95,96,97,98]. Supporting this, the expression of these enzymes directly correlates with STAT3 expression in dendritic cells [99] and gastrointestinal tract cells [100,101,102], while STAT3 suppression decreases their transcriptional activity [103,104]. This knowledge motivated our interest in studying the capability of the synthesized compounds to modulate the activity of key glycolytic enzymes.

It is well known that, to sustain continuous proliferation, metastasis, and angiogenesis, cancer cells adapt through metabolic reprogramming [105,106,107]. The complexity of the metabolic phenotype in tumor cells poses a significant challenge for effective anticancer therapy. Although more than 70 years have passed since changes in cancer cell metabolism were discovered and Otto Warburg formulated the concept of aerobic glycolysis predominance in tumors, only a small number of metabolic drugs have been successfully developed [108]. However, limitations in their use create a need for new therapeutic strategies targeting tumor metabolism. A promising direction is targeting glycolysis, which is the dominant energy source in breast [109,110,111], lung [112,113], prostate [114,115], gastrointestinal [116,117], and other cancers [118,119,120]. This is mainly due to aberrant functioning of the glycolysis rate-limiting enzymes HK2, PFK1, and PKM2.

**Figure 10:**
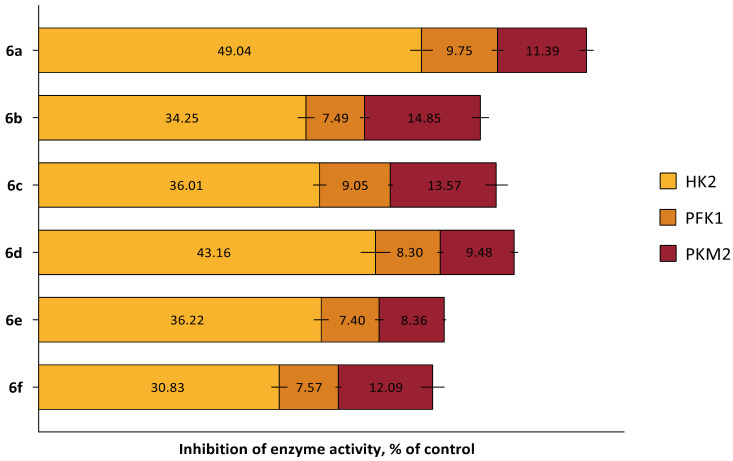
Effect of new heterocyclic quinones **6a**–**6f** at a concentration of 1/2 IC_50_ on the activity of rate-limiting glycolytic enzymes—hexokinase II, phosphofructokinase I, and pyruvate kinase M2—in MCF-7 cells. Data are presented as mean ± SEM, *n* = 3.

The inhibitory effects of the heterocyclic quinones on key glycolytic enzymes were studied according to the manufacturer’s instructions for commercial kits (#MAK091, MAK072, MAK093, Sigma Aldrich, St. Louis, MO, USA). As shown in Figure 10, a 24-h incubation of human breast cancer (MCF-7) cells with all new compounds at a concentration of half their IC_50_ resulted in a significant decrease in hexokinase II enzymatic activity but did not affect the other two enzymes analyzed. It is important to note that this concentration was chosen to maintain the viability of most cells while still being sufficient to induce metabolic stress.

In this experiment, we also observed that the HK2-inhibitory effect depended on the substituent in molecule (Figure 10). Compounds containing two identical halogen atoms (two chlorine atoms **6d** and two bromine atoms **6a**) exhibited the most favorable hexokinase inhibition profile, reducing enzyme activity by more than 40%. Interestingly, in line with in silico studies, **6a** demonstrated the strongest HK2 inhibition, reaching nearly 50%. It is plausible that modifying incubation conditions of **6a** with cells could enhance this effect, but further research is necessary, as increasing compound concentrations causes significant cell death, preventing reliable assessment of enzymatic activity.

Thus, the comprehensive in silico and in vitro study of possible cytotoxic mechanisms of the synthesized heterocyclic quinones demonstrated that the ability of **6a**–**6f** to reduce the viability of tumor-origin cells may be mediated by their involvement in the pathological STAT3/HK2 pathway.

### 2.4. In Silico ADME Profile of New Heterocyclic Quinones ***6a***–***6f***

It is well known that researchers continuously strive to develop potential therapeutic agents with excellent pharmacological activity. However, their clinical implementation often faces challenges due to poor pharmacokinetic and pharmacodynamic properties of the molecules [121]. This underscores the critical importance of studying absorption, distribution, metabolism, and excretion (ADME) properties of new compounds as a primary task within the unified drug development cycle [122]. It is also important to emphasize that evaluating the ADME profile of potential drug candidates at this stage is crucial, as it allows early identification of inherently unpromising compounds and prevents unnecessary experiments aimed at deepening the study of therapeutic potential.

To this end, we used the ADMETlab 3.0 web tool, an integrated online platform for accurate and comprehensive ADME property prediction [123].

As shown in Table 2, all synthesized molecules possess favorable physicochemical properties typical of drug-like compounds. This is evidenced by the lack of violations of empirical rules defining drug-likeness. Specifically, the molecular weights of **6a**–**6f** do not exceed 500 g/mol, the number of hydrogen bond donors and acceptors is less than 5 and 10, respectively, and the octanol–water partition coefficient (log P) ranges from 1.69 to 2.21, fully complying with the requirement of ≤5. All of this highlights the feasibility of further detailed pharmacokinetic profile analyses of the synthesized compounds.

Equally important physicochemical property is the logarithm of aqueous solubility (logS). It is well known that low solubility impairs gastrointestinal absorption of drug agents, underscoring the critical importance of logS. In our case, compounds **6d** and **6f** were predicted to have favorable solubility, with logS values ranging from −4 to −0.5. Meanwhile, **6a**–**6c**, and **6e** showed slightly lower values (<−4), but these deviations were minor and allowed continuation of the evaluation of other parameters.

**Figure 11:**
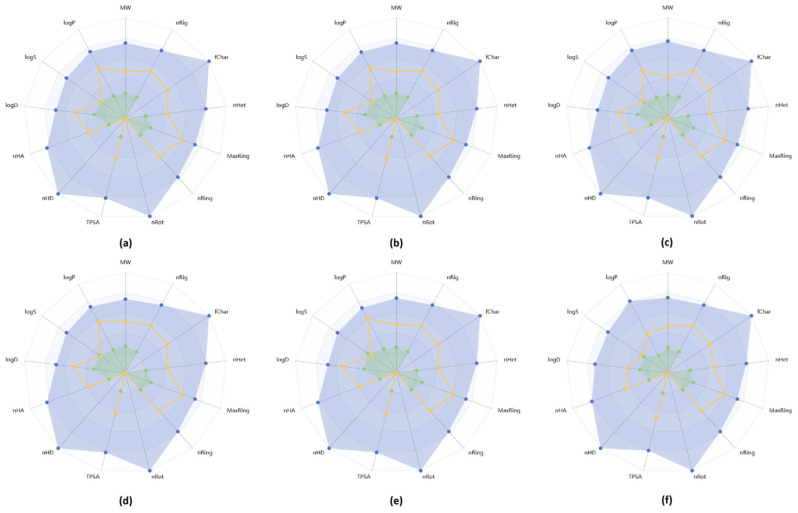
Physicochemical profile of heterocyclic quinones **6a**–**6f** (**a**–**f**), presented as radar charts obtained from the ADMETlab 3.0 web server. Blue area—lower limit, green area—upper limit, yellow line—compound properties.

Figure 11 presents radar charts generated using the ADMETlab 3.0 web tool to visually illustrate the conformity of the molecules’ physicochemical properties with those of drug-like compounds. The parameters of all studied molecules fall within the acceptable upper and lower predicted limits.

Further analysis of absorption parameters (Table 3), specifically Caco-2 and MDCK permeability, revealed that all molecules exhibit adequate permeability across the Caco-2 cell line but not MDCK. Caco-2 permeability values reached −4.57, which is above the acceptable threshold of >−5.15 log cm/s, suggesting good passive diffusion of **6a**–**6f** across intestinal cell membranes [124]. In contrast, the calculated MDCK permeability values ranged from −4.71 to −4.42, indicating low permeability (<2 × 10^−6^ cm/s) and, consequently, an inability of **6a**–**6f** to cross the blood–brain barrier. However, this characteristic is critical for positioning compounds as neuroprotective agents [125] rather than anticancer drugs. Therefore, this finding does not impose serious limitations on using these molecules for anticancer therapy, and on the contrary, it may help avoid side effects in the central nervous system.

Special attention should be given to the predicted oral bioavailability results. This parameter is a key pharmacokinetic characteristic of potential drug candidates, determining the fraction of the drug that actually reaches systemic circulation after oral administration [126]. Poor bioavailability is a major cause of failures in late-stage drug development, leading to ineffective treatment and even unpredictable adverse reactions [127]. In our case, using a predictive model for **6a**–**6f** potential bioavailability, all synthesized heterocyclic quinones demonstrated a promising oral bioavailability profile, with a maximum predicted level of up to 50%.

We also analyzed the excretion profile of the molecules. It was found that most molecules demonstrate favorable plasma clearance and half-life values. Compounds **6a**–**6c** and **6f** are characterized by low clearance (<5 mL/min/kg), while **6d** and **6e** show moderate clearance (5–15 mL/min/kg). These results correlate with the molecules’ half-life data, which reflect their excretion rates. The shortest half-lives were observed for **6d** and **6e** (approximately 50 and 65 min, respectively), which is consistent with the higher clearance.

It is also interesting to note a certain dependence of the predicted effect on the molecular structure. The molecule **6a**, which is structurally distinct due to the presence of two bromine atoms, exhibits the lowest clearance and the longest half-life.

Thus, as a result of the in silico ADME profile study of this series of new heterocyclic quinones, we identified favorable pharmacokinetic properties that anticancer drugs should possess. This supports the rationale for further investigation of the therapeutic potential of these compounds.

## 3. Materials and Methods

### 3.1. Chemistry

#### 3.1.1. Reagents and Materials

The solvents and reagents used for this project were purchased from Acros Organics (Geel, Belgium) and used without purification unless otherwise stated. The melting points of compounds were determined on a PolyTherm A (Munchen, Germany) heating stage at a heating rate of 4 °C/min and were not corrected. ^1^H and ^13^C NMR spectra were recorded on a Bruker (Billerica, MA, USA) Bruker DRX400 instrument (the frequencies for ^1^H and ^13^C were 400 and 100 MHz, respectively) in DMSO-d_6_ at 303 K (^1^H and ^13^C NMR spectra are in Supplementary Materials). The residual proton (δ 2.5) and carbon (δ 39.5) signals of the DMSO-d_6_ were used as internal standards. The assignment of the proton spectra was carried out using 2D {^1^H-^1^H} NOESY spectroscopy. High resolution mass spectra were recorded on a MicrOTOF II instrument (Bruker Daltonics, Billerica, MA, USA) using electrospray ionization. The mass scanning range (*m*/*z* 50) was 3000 Da; the compounds were injected with a syringe. MeCN and MeOH solvents were used; the solution flow rate was 4.0 μL/min. The FTIR spectra were obtained using a PerkinElmer Spectrum 65 FT-IR Spectrometer (PerkinElmer Inc., Shelton, CT, USA) and the Attenuated Total Reflection (ATR) technique, which employs a Zinc Selenide (ZnSe) crystal to measure the infrared absorption of a sample.

#### 3.1.2. Synthesis and General Procedures

General procedure for the synthesis of monohalogenated condensed benzimidazole derivatives (**2a**–**d**).

To a solution of 0.01 mol of *ortho*-nitro-*tert*-anilines **1a**–**d** in 70 mL of 8% HCl at 80 °C, 2.7 g (0.012 mol) of SnCl_2_·2H_2_O in 70 mL of 8% HCl was added dropwise over 2 h. After cooling, the reaction mixture was poured into ice and treated with NH_4_OH to pH = 7–8. The product was extracted with hot chloroform. After chloroform distillation, the product was dried and crystallized in methanol.

7-Bromo-1,2,3,4-tetrahydropyrido[1,2-*a*]benzimidazole (**2a**): Yield 93%. m.p. 158–160 °C. ^1^H NMR (400 MHz, DMSO-*d_6_*) *δ* 7.71 (d, *J* = 2.1 Hz, 1H, H^6^), 7.41 (d, *J* = 8.7 Hz, 1H, H^9^), 7.29 (dd, *J* = 8.7 Hz, 2.1 Hz, 1H, H^8^), 4.07 (t, *J* = 6.1 Hz, 2H, H^1,1^), 2.93 (t, *J* = 6.3 Hz, 2H, H^4,4^), 2.06–1.96 (m, 2H, H^2,2^), 1.94–1.88 (m, 2H, H^3,3^). ^13^C NMR (101 MHz, DMSO-*d_6_*) *δ* 153.79, 144.64, 134.34, 124.32, 121.10, 114.38, 111.93, 42.92, 25.53, 22.52, 20.64. FT-IR (cm^−1^): 1612 *ν* (C=N, imidazole). HRMS: (ESI/TOF) *m*/*z* calculated for C_11_H_12_BrN_2_ 252.1298 [M + H]^+^, Found: 252.1087.

7-Chloro-1,2,3,4-tetrahydropyrido[1,2-*a*]benzimidazole (**2b**): Yield 94%. m.p. 147–149 °C. ^1^H NMR (400 MHz, DMSO-*d_6_*) *δ* 7.56 (d, *J* = 1.9 Hz, 1H, H^6^), 7.47 (d, *J* = 8.6 Hz, 1H, H^9^), 7.19 (dd, *J* = 8.6 Hz, 1.9 Hz, 1H, H^8^), 4.08 (t, *J* = 6.1 Hz, 2H, H^1,1^), 2.95 (t, *J* = 6.3 Hz, 2H, H^4,4^), 2.06–1.98 (m, 2H, H^2,2^), 1.95–1.89 (m, 2H, H^3,3^). ^13^C NMR (101 MHz, DMSO-*d_6_*) *δ* 154.0, 144.1, 134.0, 126.6, 121.7, 118.1, 111.4, 42.9, 25.6, 22.5, 20.6. FT-IR (cm^−1^): 1614 *ν* (C=N, imidazole). HRMS: (ESI/TOF) *m*/*z* calculated for C_11_H_12_ClN_2_ [M + H]^+^: 207.6788. Found: 207.6783.

7-Chloro-3-methyl-1,2,3,4-tetrahydropyrido[1,2-*a*]benzimidazole (**2c**): Yield 92%. m.p. 127–129 °C. ^1^H NMR (400 MHz, DMSO-*d_6_*) *δ* 7.54 (d, *J* = 1.3 Hz, 1H, H^6^), 7.45 (d, *J* = 8.4 Hz, 1H, H^9^), 7.18 (dd, *J* = 8.5 Hz, 1.8 Hz, 1H, H^8^), 4.16–4.26 (m, 1H, H^1^), 3.87–3.97 (m, 1H, H^1′^), 2.95–3.07 (m, 1H, H^4′^), 2.57–2.46 (m, 2H, H^4^), 2.12–2.01 (m, 2H, H^2,2^), 1.61–1.75 (m, 1H, H^3^), 1.08 (d, *J* = 6.4 Hz, 3H, CH_3_). ^13^C NMR (101 MHz, DMSO-*d_6_*) *δ* 154.13, 144.07, 133.87, 126.64, 121.87, 118.09, 111.59, 42.11, 33.31, 30.14, 27.34, 21.38. FT-IR (cm^−1^): 1615 *ν* (C=N, imidazole). HRMS: (ESI/TOF) *m*/*z* calculated for C_12_H_14_ClN_2_ [M + H]^+^: 221.7054. Found: 221.7059.

8-Chloro-3,4-dihydro-1H-[1,4]oxazino[4,3-a]benzimidazole (**2d**): Yield 90%. m.p. 193–196 °C. ^1^H NMR (400 MHz, DMSO-*d_6_*) *δ* 7.64 (d, *J* = 1.9 Hz, 1H, H^9^), 7.54 (d, *J* = 8.6 Hz, 1H, H^6^), 7.26 (dd, *J* = 8.5 Hz, 1.9 Hz, 1H, H^7^), 4.95 (s, 2H, H^1^), 4.23–4.12 (m, 4H, H^3,3,4,4^). ^13^C NMR (101 MHz, DMSO-*d_6_*) *δ* 150.44, 143.86, 133.61, 127.02, 122.44, 118.77, 111.82, 65.01, 63.91, 42.62. FT-IR (cm^−1^): 1603 *ν* (C=N, imidazole). HRMS: (ESI/TOF) *m*/*z* calculated for C_10_H_10_ClN_2_O [M + H]^+^: 209.6517. Found: 209.6521.

General procedure for the synthesis of dihalogenated condensed benzimidazole derivatives (**3a**–**f**).

A solution of 0.018 mol of NBS/NCS in 70 mL of H_2_SO_4_ was slowly added dropwise for 4 h at 35 °C to a solution of 0.01 mol of the monohalogen derivative **2a**–**c** in 50 mL of H_2_SO_4_. After cooling, the reaction mixture was poured into ice and treated with NH_4_OH to pH = 7–8. The precipitate was filtered, dried, and crystallized in methanol.

7,8-dibromo-1,2,3,4-tetrahydropyrido[1,2-*a*]benzimidazole (**3a**): Yield 93%. m.p. 184–187 °C. ^1^H NMR (400 MHz, DMSO-*d_6_*) *δ* 7.92 (s, 1H, H^6^), 7.89 (s, 1H, H^9^), 4.07 (t, *J* = 6.0 Hz, 2H, H^1,1^), 2.94 (t, *J* = 6.3 Hz, 2H, H^4,4^), 2.06–1.86 (m, 4H, H^2,2,3,3^). ^13^C NMR (101 MHz, DMSO-*d_6_*) *δ* 155.06, 144.14, 135.95, 123.08, 116.23, 115.76, 114.90, 43.22, 25.62, 22.57, 20.60. FT-IR (cm^−1^): 1614 *ν* (C=N, imidazole). HRMS: (ESI/TOF) *m*/*z* calculated for C_11_H_11_Br_2_N_2_: 331.0259 [M + H]^+^. Found: 331.0265.

7-bromo-8-chloro-1,2,3,4-tetrahydropyrido[1,2-*a*]benzimidazole (**3b**): Yield 89%. m.p. 184–188 °C. ^1^H NMR (400 MHz, DMSO-*d_6_*) *δ* 7.88 (s, 1H, H^6^), 7.79 (s, 1H, H^9^), 4.07 (t, *J* = 6.0 Hz, 2H, H^1,1^), 2.95 (t, *J* = 6.3 Hz, 2H, H^4,4^), 2.06–1.88 (m, 4H, H^2,2,3,3^). ^13^C NMR (101 MHz, DMSO-*d_6_*) *δ* 155.01, 143.14, 135.33, 125.81, 122.90, 114.16, 112.03, 43.18, 25.54, 22.39, 20.51. FT-IR (cm^−1^): 1615 *ν* (C=N, imidazole). HRMS: (ESI/TOF) *m*/*z* calculated for C_11_H_11_BrClN_2_: 286.5749 [M + H]^+^. Found: 286.5746.

8-bromo-7-chloro-1,2,3,4-tetrahydropyrido[1,2-*a*]benzimidazole (**3c**): Yield 91%. m.p. 166–169 °C. ^1^H NMR (400 MHz, DMSO-*d_6_*) *δ* 7.92 (s, 1H, H^9^), 7.76 (s, 1H, H^6^), 4.08 (t, *J* = 6.0 Hz, 2H, H^1,1^), 2.95 (t, *J* = 6.3 Hz, 2H, H^4,4^,), 2.04–1.96 (m, 2H, H^2,2^), 1.94–1.86 (m, 2H, H^3,3^). ^13^C NMR (101 MHz, DMSO-*d_6_*) *δ* 155.09, 143.41, 135.19, 126.34, 119.77, 115.11, 113.72, 43.26, 25.49, 22.41, 20.54. FT-IR (cm^−1^): 1615 *ν* (C=N, imidazole). HRMS: (ESI/TOF) *m*/*z* calculated for C_11_H_11_BrClN_2_: 286.5749 [M + H]^+^. Found: 286.57451.

7,8-dichloro-1,2,3,4-tetrahydropyrido[1,2-*a*]benzimidazole (**3d**): Yield 90%. m.p. 209–212 °C. ^1^H NMR (400 MHz, DMSO-*d_6_*) *δ* 7.76 (s, 1H, H^6^), 7.73 (s, 1H, H^9^), 4.06 (t, *J* = 6.0 Hz, 2H, H^1,1^), 2.94 (t, *J* = 6.3 Hz, 2H, H^4,4^), 2.06–1.96 (m, 2H, H^2,2^), 1.94–1.86 (m, 2H, H^3,3^). ^13^C NMR (101 MHz, DMSO-*d_6_*) *δ* 155.14, 142.94, 134.97, 124.79, 124.40, 119.79, 111.80, 43.23, 25.60, 22.53, 20.57. FT-IR (cm^−1^): 1616 *ν* (C=N, imidazole). HRMS: (ESI/TOF) *m*/*z* calculated for C_11_H_11_Cl_2_N_2_: 242.1239 [M + H]^+^. Found: 242.1241.

8-bromo-7-chloro-3-methyl-1,2,3,4-tetrahydropyrido[1,2-*a*]benzimidazole (**3e**): Yield 93%. m.p. 165–167 °C. ^1^H NMR (400 MHz, DMSO-*d_6_*) *δ*. 7.90 (s, 1H, H^6^), 7.75 (s, 1H, H^9^), 4.27–4.19 (m, 1H, H^1^), 3.97–3.87 (m, 1H, H^1’^), 3.08–2.99 (m, 1H, H^4’^), 2.59–2.47 (m, 1H, H^4^), 2.14–2.03 (m, 2H, H^2,2^), 1.74–1.66 (m, 1H, H^3^), 1.09 (d, *J* = 6.5 Hz, 3H, CH_3_). ^13^C NMR (101 MHz, DMSO-*d_6_*) *δ* 155.17, 143.66, 135.04, 126.32, 119.49, 115.22, 113.69, 42.41, 33.47, 27.31, 21.38. FT-IR (cm^−1^): 1618 *ν* (C=N, imidazole). HRMS: (ESI/TOF) *m*/*z* calculated for C_12_H_13_BrClN_2_: 300.6015 [M + H]^+^. Found: 300.6021.

7-bromo-8-chloro-3,4-dihydro-1H-[1,4]oxazino[4,3-a]benzimidazole (**3f**): Yield 91%. m.p. 192–199 °C. ^1^H NMR (400 MHz, DMSO-*d_6_*) *δ* 8.02 (s, 1H, H^6^), 7.84 (s, 1H, H^9^), 4.94 (s, 2H, H^1^), 4.09–4.23 (m, 4H, H^3,3,4,4^). ^13^C NMR (101 MHz, DMSO-*d_6_*) *δ* 151.43, 143.09, 134.71, 126.78, 120.64, 115.57, 114.42, 65.01, 63.91, 43.12. FT-IR (cm^−1^): 1605 *ν* (C=N, imidazole). HRMS: (ESI/TOF) *m*/*z* calculated for C_10_H_9_BrClN_2_O: 288.5477 [M + H]^+^. Found: 288.5471.

General procedure for the synthesis of condensed benzimidazole nitro derivatives (**4c**,**d**) and mixtures of nitro compounds (**4a**–**f** and **4’a**–**f**).

To a solution of 0.01 mol of the dihalogen derivative of 1,2,3,4-tetrahydropyrido[1,2-*a*]benzimidazole **3a**–**f** in 50 mL of H_2_SO_4_ at 35 °C, 0.011 mol of KNO_3_ in 50 mL of H_2_SO_4_ was added. The nitrating mixture was added over 2h for the synthesis of **4c**,**d** and over 0.1 min for the synthesis of mixtures **4a**–**f** and **4′a**–**f**. A mixture of nitro compounds **4a**–**f** and **4′a**–**f** was obtained over 1h at 35 °C. The reaction mixture was poured into ice and treated with NH_4_OH to pH 7–8. The precipitate was filtered and dried. The total yield of nitro derivatives **4** and **4′** was 92–96%. Mixtures of nitro compounds **4a**–**f** and **4′a**–**f** were used for reduction without purification. Nitro compounds **4c**,**d** were crystallized in methanol. The separation of the mixture of products **4a** and **4′a** was carried out by crystallization of the reaction mixture in chloroform. Upon cooling, isomer **4a** fell out of the chloroform, which was separated by filtration. The filtrate was evaporated. The dry residue was crystallized in methanol, from which the **4′a** isomer precipitated.

7,8-dibromo-6-nitro-1,2,3,4-tetrahydropyrido[1,2-*a*]benzimidazole (**4a**): Yield 63%. m.p. 182–186 °C. ^1^H NMR (400 MHz, DMSO-*d_6_*) *δ* 8.27 (s, 1H, H^9^), 4.15 (t, *J* = 6.0 Hz, 2H, H^1,1^), 2.99 (t, *J* = 6.3 Hz, 2H, H^4,4^), 2.07–1.97 (m, 2H, H^2,2^), 1.95–1.86 (m, 2H, H^3,3^). ^13^C NMR (101 MHz, DMSO-*d_6_*) *δ* 157.79, 141.38, 137.25, 135.33, 117.70, 116.34, 107.51, 43.78, 25.52, 22.05, 20.07. FT-IR (cm^−1^): 1625 *ν* (C=N, imidazole), 1537 *ν* (C_Ar_-NO_2_). HRMS: (ESI/TOF) *m*/*z* calculated for C_11_H_10_Br_2_N_3_O_2_ 376.0235 [M + H]^+^. Found: 376.0239.

7,8-dibromo-9-nitro-1,2,3,4-tetrahydropyrido[1,2-*a*]benzimidazole (**4′a**): Yield 11%. m.p. 203–207 °C. ^1^H NMR (400 MHz, DMSO-*d_6_*) *δ* 8.19 (s, 1H, H^6^), 3.85 (t, *J* = 6.1 Hz, 2H, H^1,1^), 3.01 (t, *J* = 6.3 Hz, 2H, H^4,4^), 2.06–1.96 (m, 2H, H^2,2^), 1.95–1.85 (m, 2H, H^3,3^). ^13^C NMR (101 MHz, DMSO-*d_6_*) *δ* 157.59, 146.25, 136.98, 125.37, 117.46, 116.99, 108.15, 43.73, 26.00, 22.41, 19.66. FT-IR (cm^−1^): 1621 *ν* (C=N, imidazole), 1529 *ν* (C_Ar_-NO_2_). HRMS: (ESI/TOF) *m*/*z* calculated for C_11_H_10_Br_2_N_3_O_2_ 376.0235 [M + H]^+^. Found: 376.0241.

8-bromo-7-chloro-6-nitro-1,2,3,4-tetrahydropyrido[1,2-*a*]benzimidazole (**4c**): Yield 91%. m.p. 196–200 °C. ^1^H NMR (400 MHz, DMSO-*d_6_*) *δ* 8.31 (s, 1H, H^9^), 4.17 (t, 2H, H^1^, *J* = 6.1 Hz), 3.00 (t, 2H, H^4^, *J* = 6.3 Hz), 2.08–1.98 (m, 2H, H^2^), 1.94–1.86 (m, 2H, H^3^). ^13^C NMR (101 MHz, DMSO-*d_6_*) *δ* 158.08, 139.15, 136.72, 135.33, 117.91, 117.23, 114.10, 43.79, 25.56, 22.05, 20.05. FT-IR (cm^−1^): 1624 *ν* (C=N, imidazole), 1534 *ν* (C_Ar_-NO_2_). HRMS: (ESI/TOF) *m*/*z* calculated for C_11_H_10_BrClN_3_O_2_ 331.5725 [M + H]^+^. Found: 331.5731.

7,8-dichloro-6-nitro-1,2,3,4-tetrahydropyrido[1,2-*a*]benzimidazole (**4d**): Yield 89%. m.p. 190–194 °C. ^1^H NMR (400 MHz, DMSO-*d_6_*) *δ* 8.17 (s, 1H, H^9^), 4.15 (t, *J* = 6.0 Hz, 2H, H^1,1^), 3.00 (t, *J* = 6.3 Hz, 2H, H^4,4^), 2.11–1.97 (m, 2H, H^2,2^), 1.94–1.84 (m, 2H, H^3,3^). ^13^C NMR (101 MHz, DMSO-*d_6_*) *δ* 158.16, 139.17, 136.18, 134.87, 124.69, 115.82, 114.92, 43.79, 25.56, 22.05, 20.06. FT-IR (cm^−1^): 1623 *ν* (C=N, imidazole), 1530 *ν* (C_Ar_-NO_2_). HRMS: (ESI/TOF) *m*/*z* calculated for C_11_H_10_Cl_2_N_3_O_2_ 287.1215 [M + H]^+^. Found: 287.1209.

General procedure for the synthesis of condensed benzimidazole amino derivatives (**5c**,**d**) and mixtures of amino compounds (**5a**–**f** and **5’a**–**f**).

To a solution of 0.01 mol of 1,2,3,4-tetrahydropyrido[1,2-*a*]benzimidazole nitro derivative **4c**,**d** or a mixture of isomeric nitro compounds **4a**–**f** and **4′a**–**f** in 25 mL of *i*-PrOH and 25 mL of 10% HCl at 60 °C, 0.07 mol of a 15% TiCl_3_ solution in 10% HCl was added, and stirred for 5 min. The reaction mixture was treated with NH_4_OH to pH 7–8 and extracted with chloroform. After distillation of chloroform, amino compounds **5c**,**d** or mixtures of isomeric amino compounds **5a**–**f** and **5′a**–**f** were obtained. The total yield of the mixture of amino derivatives **5** and **5′** was 93–97%.

8-bromo-7-chloro-1,2,3,4-tetrahydropyrido[1,2-*a*]benzimidazol-6-amine (**5c**): Yield 95%. m.p. 165–169 °C. ^1^H NMR (400 MHz, DMSO-*d_6_*) *δ* 7.11 (s, 1H, H^9^), 5.61 (s, 2H, NH_2_), 3.99 (t, 2H, H^1^, *J* 6.0 Hz), 2.92 (t, 2H, H^4^, *J* 6.2 Hz), 2.08–1.96 (m, 2H, H^2^), 1.94–1.86 (m, 2H, H^3^). ^13^C NMR (101 MHz, DMSO-*d_6_*) *δ* 150.88, 137.64, 134.20, 130.88, 115.14, 108.40, 102.52, 43.03, 25.42, 22.56, 20.69. FT-IR (cm^−1^): 3442 *ν* (C_Ar_-NH), 3297 *ν* (C_Ar_-NH), 1613 *ν* (C=N, imidazole). HRMS: (ESI/TOF) *m*/*z* calculated for C_11_H_12_BrClN_3_ 301.5895 [M + H]^+^. Found: 301.5895.

7,8-dichloro-1,2,3,4-tetrahydropyrido[1,2-*a*]benzimidazol-6-amine (**5d**)**.** Yield 94%. m.p. 249–252 °C. ^1^H NMR (400 MHz, DMSO-*d_6_*) *δ* 6.96 (s, 1H, H^9^), 5.64 (s, 2H, NH_2_), 3.98 (t, *J* = 6.0 Hz, 2H, H^1,1^), 2.91 (t, *J* = 6.3 Hz, 2H, H^4,4^), 2.04–1.95 (m, 2H, H^2,2^), 1.94–1.84 (m, 2H, H^3,3^). ^13^C NMR (101 MHz, DMSO-*d_6_*) *δ* 150.92, 137.65, 133.54, 130.36, 125.35, 106.81, 99.44, 43.02, 25.42, 22.54, 20.69. FT-IR (cm^−1^): 3456 *ν* (C_Ar_-NH), 3303 *ν* (C_Ar_-NH), 1614 *ν* (C=N, imidazole). HRMS: (ESI/TOF) *m*/*z* calculated for C_11_H_12_Cl_2_N_3_ 257.1385 [M + H]^+^. Found: 257.1393.

General procedure for the synthesis of condensed benzimidazole diones **6a**–**f**.

To a solution of 0.1 mol KNO_3_ in 20 mL H_2_SO_4_ was added to a solution of 0.01 mol of individual amino compounds **5** or mixtures of amino products **5** and **5′** in 25 mL H_2_SO_4_. The reaction mixture was stirred for 8 h at 20 °C. Next, the reaction mixture was poured into ice and treated with NH_4_OH to pH 8. The precipitate was filtered, dried, and crystallized into DMFA.

7,8-dibromo-1,2,3,4-tetrahydropyrido[1,2-*a*]benzimidazole-6,9-dione (**6a**): Yield 90%. m.p. 202–205 °C. ^1^H NMR (400 MHz, DMSO-*d_6_*) *δ* 4.23 (t, *J* = 6.1 Hz, 2H, H^1,1^), 2.90 (t, *J* = 6.3 Hz, 2H, H^4,4^), 2.04–1.84 (m, 4H H^2,2,3,3^). ^13^C NMR (101 MHz, DMSO-*d_6_*) *δ* 171.68, 168.37, 153.35, 140.49, 139.55, 139.22, 130.15, 45.98, 25.03, 22.05, 19.59. FT-IR (cm^−1^): 1669 *ν* (C=O, 1,4-benzoquinones), 1611 *ν* (C=N, imidazole). HRMS: (ESI/TOF) *m*/*z* calculated for C_11_H_9_Br_2_N_2_O_2_ 361.0088 [M + H]^+^. Found: 361.0106.

7-bromo-8-chloro-1,2,3,4-tetrahydropyrido[1,2-*a*]benzimidazole-6,9-dione (**6b**): Yield 91%. m.p. 175–180 °C. ^1^H NMR (400 MHz, DMSO-*d_6_*) *δ* 4.22 (t, *J* = 5.9 Hz, 2H, H^1,1^), 2.89 (t, *J* = 6.3 Hz, 2H, H^4,4^), 2.04–1.94 (m, 2H, H^2,2^), 1.92–1.84 (m, 2H, H^3,3^). ^13^C NMR (101 MHz, DMSO-*d_6_*) *δ* 172.08, 167.74, 153.46, 143.17, 140.62, 135.88, 130.36, 46.00, 25.03, 22.04, 19.58. FT-IR (cm^−1^): 1676 *ν* (C=O, 1,4-benzoquinones), 1613 *ν* (C=N, imidazole). HRMS: (ESI/TOF) *m*/*z* calculated for C_11_H_9_BrClN_2_O_2_ 316.5578 [M + H]^+^. Found: 316.5569.

8-bromo-7-chloro-1,2,3,4-tetrahydropyrido[1,2-*a*]benzimidazole-6,9-dione (**6c**): Yield 93%. m.p. 195–199 °C. ^1^H NMR (400 MHz, DMSO-*d*_6_) δ 4.23 (t, *J* = 5.9 Hz, 2H, H^1,1^), 2.90 (t, *J* = 6.2 Hz, 2H, H^4,4^), 2.03–1.94 (m, 2H, H^2,2^), 1.93–1.82 (m, 2H, H^3,3^). ^13^C NMR (101 MHz, DMSO-*d*_6_) δ 171.15, 168.79, 153.46, 143.46, 140.71, 135.58, 130.23, 46.03, 25.05, 22.06, 19.60. FT-IR (cm^−1^): 1671 *ν* (C=O, 1,4-benzoquinones), 1612 *ν* (C=N, imidazole). HRMS: (ESI/TOF) *m*/*z* calculated for C_11_H_9_BrClN_2_O_2_ 316.5578 [M + H]^+^. Found: 316.5571.

7,8-dichloro-1,2,3,4-tetrahydropyrido[1,2-*a*]benzimidazole-6,9-dione (**6d**): Yield 93%. m.p. 191–194 °C. ^1^H NMR (400 MHz, DMSO-*d*_6_) δ 4.23 (t, *J* = 6.0 Hz, 2H, H^1,1^,), 2.90 (t, *J* = 6.3 Hz, 2H, H^4,4^), 2.00–1.94 (m, 2H, H^2,2^), 1.91–1.85 (m, 2H, H^3,3^). ^13^C NMR (101 MHz, DMSO-*d*_6_) δ 171.57, 168.21, 153.59, 140.82, 140.21, 139.95, 130.43, 46.05, 25.05, 22.06, 19.59. FTIR (cm^−1^): 1678 *ν* (C=O, 1,4-benzoquinones), 1615 *ν* (C=N, imidazole). HRMS: (ESI/TOF) *m*/*z* calculated for C_11_H_9_Cl_2_N_2_O_2_ 272.1068 [M + H]^+^. Found: 272.1061.

8-bromo-7-chloro-3-methyl-1,2,3,4-tetrahydropyrido[1,2-*a*]benzimidazole-6,9-dione (**6e**): Yield 92%. m.p. 225–229 °C. ^1^H NMR (400 MHz, DMSO-*d*_6_) *δ* 4.44–4.36 (m, 1H, H^1^), 4.12–4.01 (m, 1H, H^1’^), 3.07–2.96 (m, 1H, H^4’^), 2.56–2.42 (m, 1H, H^4^), 2.09–2.00 (m, 2H, H^2,2^), 1.72–1.58 (m, 1H, H^3^), 1.08 (d, *J* = 6.5 Hz, 3H, CH_3_). ^13^C NMR (101 MHz, DMSO-*d*_6_) *δ* 171.08, 168.73, 153.58, 143.62, 141.07, 135.51, 130.34, 45.14, 32.87, 29.79, 26.45, 20.98. FT-IR (cm^−1^): 1682 *ν* (C=O, 1,4-benzoquinones), 1618 *ν* (C=N, imidazole). HRMS: (ESI/TOF) *m*/*z* calculated for C_12_H_11_BrClN_2_O_2_ 330.5843 [M + H]^+^. Found: 330.5849.

7-bromo-8-chloro-3,4-dihydro-1H-[1,4]oxazino[4,3-a]benzimidazole-6,9-dione (**6f**): Yield 88%. m.p. 195–198 °C. ^1^H NMR (400 MHz, DMSO-*d*_6_) *δ* 4.91 (s, 2H, H^1^), 4.31 (t, 2H, H^3^, *J* = 5.3 Hz), 4.07 (t, 2H, H^4^, *J* = 5.3 Hz). ^13^C NMR (101 MHz, DMSO-*d*_6_) δ 172.25, 169.05, 155.86, 145.06, 141.21, 137.18, 131.73, 67.56, 64.97, 44.84. FT-IR (cm^−1^): 1667 *ν* (C=O, 1,4-benzoquinones), 1608 *ν* (C=N, imidazole). HRMS: (ESI/TOF) *m*/*z* calculated for C_10_H_7_BrClN_2_O_3_ 318.5306 [M + H]^+^. Found: 318.5311.

### 3.2. Biological Evaluation

#### 3.2.1. Cell Lines and Their Cultivation

Human lung adenocarcinoma A549 cells, breast carcinoma MCF-7 cells, colon adenocarcinoma SW480 cells, neuroblastoma SH-SY5Y cells, and normal human embryonic kidney HEK-293 cells were obtained from the collection of the Institute of Cytology of the Russian Academy of Sciences (Saint Petersburg, Russia). Cultivation was performed in complete DMEM supplemented with glutamax, containing 10% fetal bovine serum and 1% penicillin-streptomycin. Cell cultures were incubated at 37 °C in a humidified atmosphere with 5% CO_2_.

#### 3.2.2. Cytotoxicity Profile Study

The MTT assay was used to determine the cytotoxic profile of the synthesized compounds [128]. This method is based on the reduction of the tetrazolium salt (3-(4,5-dimethylthiazol-2-yl)-2,5-diphenyl tetrazolium bromide) by mitochondrial dehydrogenases of living cells to water-insoluble formazan crystals.

Cells in the exponential growth phase were seeded in 96-well culture plates. After 24 h, the test compounds were added to each well at various concentrations (from 0.01 to 100 µM) in triplicate. Control wells contained 1% DMSO solution.

Following a 24-h incubation with the compounds, 5 mg/mL MTT solution was added to each well, and the cells were incubated at 37 °C for 2 h. Then, the MTT solution was carefully removed, and 200 µL DMSO was added to dissolve the formed formazan crystals. Optical density was measured at a wavelength of 540 nm.

The efficacy indicators of the cytotoxic effect were IC_50_ values, representing compound concentrations causing 50% cell death in the sample.

#### 3.2.3. Study of the Compounds’ Effect on Apoptosis

A549 cells were seeded in six-well plates at 1 × 10^6^ cells/well in a total volume of 2 mL. After 24 h of incubation, the compounds were added at concentrations equal to their IC_50_ cytotoxic effect.

Cells were then harvested by centrifugation at 2000 rpm for 5 min and washed twice with ice-cold PBS, followed by resuspension in binding buffer. Samples were incubated with 5 µL of the Annexin V-Elab Fluor 647/PI apoptosis detection kit (Elabsciens, TX, USA) and 5 µL of propidium iodide for 15 min at room temperature in the dark. Finally, the cells were analyzed by flow cytometry (Guava EasyCyte, MERCK, Darmstadt, Germany) within 1 h. Experiments were performed in triplicate.

#### 3.2.4. Study of the Compounds’ Effect on the Activity of Allosteric Glycolytic Enzymes

To assess the effect of the compounds on key glycolytic enzyme activities, colorimetric assays were conducted using commercially available kits (#MAK091, MAK072, MAK093, Sigma, St. Louis, MI, USA). Sample preparation included lysis on ice of A549 lung carcinoma cells at 1 × 10^6^ cells/well, which were pre-incubated with the test compounds at IC50 concentrations for 90 min. Lysis was performed in NP-40 buffer containing 2 mM DTT and protease inhibitors, followed by centrifugation at 12,000 rpm. All subsequent analysis steps were performed according to the manufacturer’s instructions. Results are presented as mean ± standard error of the mean (SEM) with *n* = 5.

#### 3.2.5. In Silico Toxicological Characteristics and Molecular Targets of the Compounds

The toxicological profiles of the compounds were evaluated using the Cell-Line Cytotoxicity Predictor 2.0 (CLC-Pred) web service (https://www.way2drug.com/clc-pred/, accessed on 1 June 2025), and possible molecular targets of the synthesized compounds were analyzed using PASS Online (https://www.way2drug.com/passonline, accessed on 23 June 2025), both based on the PASS technology [60].

The 2D chemical structures of the compounds were drawn using the ChemDraw Ultra 12.0 program (SMILE mode).

#### 3.2.6. In Silico ADME Profile Prediction

For ADME property prediction of the synthesized compounds, we used the publicly available program ADMETlab 3.0 (https://admetlab3.scbdd.com/, accessed on 16 July 2025). This model is based on over 30 different datasets containing information on more than 400,000 molecules [123].

## 4. Conclusions

In this study, new anticancer agents were designed featuring quinone fragments and halogen atoms in a condensed benzimidazole nucleus. A convenient synthesis method from inexpensive, readily available materials was developed, with a key step being the carefully controlled reducing the intramolecular heterocyclization of ortho-nitro-tert-anilines to form the benzimidazole core without side products.

The synthesized heterocyclic quinones showed pronounced cytotoxic activity against tumor cells, confirming their potential as effective anticancer agents. In silico analysis predicted interactions with key cancer-related targets, especially STAT3, a central regulator of tumor growth, metabolism, and cell survival.

Although these compounds do not strongly induce apoptosis, they effectively inhibit key glycolytic enzymes, disrupting tumor cell metabolism and leading to cell death via a non-apoptotic mechanism of metabolic reprogramming.

Our results open new avenues for developing antitumor agents with atypical mechanisms of action targeting tumor cell metabolic pathways through STAT3-mediated modulation of glycolytic function. This approach is especially relevant in the context of tumor resistance to apoptosis inducers.

Moreover, the synthesized compounds demonstrated a favorable ADME profile, significantly enhancing their value as candidates for further preclinical and clinical development as potential drugs.

## Figures and Tables

**Table 1:** Cytotoxic profile of new heterocyclic quinones **6a**–**6f**.

IC_50_ of Cytotoxic Effect, µM					
Compound	A549 ^1^	MCF-7 ^2^	SW-480 ^3^	SH-SY5Y ^4^	Hek-293 ^5^
**6a**	9.53 ± 0.59	8.81 ± 1.65	9.11 ± 0.13	9.66 ± 0.57	24.77 ± 0.42
**6b**	8.86 ± 3.19	7.79 ± 0.03	10.54 ± 0.68	8.60 ± 0.06	16.52 ± 0.15
**6c**	7.81 ± 0.64	8.35 ± 0.32	10.22 ± 0.04	9.49 ± 0.80	16.01 ± 0.18
**6d**	7.76 ± 0.17	8.52 ± 0.23	8.68 ± 0.20	8.52 ± 0.49	15.09 ± 0.38
**6e**	8.23 ± 0.39	8.97 ± 0.05	9.53 ± 0.51	9.28 ± 0.15	20.17 ± 0.30
**6f**	11.28 ± 2.49	8.90 ± 0.19	8.44 ± 0.07	10.75 ± 0.93	25.38 ± 0.02
Mytomycin C	18.09 ± 1.80	20.37 ± 1.74	11.28 ± 0.46	7.04 ± 0.02	6.59 ± 1.46

^1^ Human non-small cell lung cancer; ^2^ Human breast adenocarcinoma cells; ^3^ Human colorectal cancer cells; ^4^ Human neuroblastoma; ^5^ Human embryonic kidney.

**Table 2:** Physicochemical properties of new heterocyclic quinones **6a**–**6f**.

Compound	Physicochemical Properties				
	MW ^1^	nHA ^2^	nHD ^3^	logS ^4^	LogP ^5^
**6a**	357.90	4	0	−4.18	4.02
**6b**	313.95	4	0	−4.08	4.27
**6c**	313.95	4	0	−4.05	3.91
**6d**	270.00	4	0	−3.82	4.74
**6e**	327.96	4	0	−4.66	5.66
**6f**	315.93	5	0	−3.69	3.25

^1^ Molecular Weight; ^2^ Number of hydrogen bond acceptors; ^3^ Number of hydrogen bond donors; ^4^ The logarithm of aqueous solubility value; ^5^ The logarithm of the n-octanol/water distribution coefficient.

**Table 3:** Properties of drug-likeness of heterocyclic quinones **6a**–**6f**.

Compound	Properties of Drug-Likeness						
	Caco-2 Permeability	MDCK Permeability	F20% ^1^	F30% ^2^	F50% ^3^	CL_plasma_ ^4^	T_1/2_ ^5^
**6a**	−4.55	−4.42	excellent	excellent	excellent	2.96	1.65
**6b**	−4.46	−4.51	excellent	excellent	excellent	4.33	1.11
**6c**	−4.46	−4.51	excellent	excellent	excellent	4.72	1.08
**6d**	−4.34	−4.71	excellent	excellent	excellent	6.01	0.81
**6e**	−4.57	−4.45	excellent	excellent	excellent	6.05	1.04
**6f**	−4.48	−4.56	excellent	excellent	excellent	4.47	1.49

^1^ The human oral bioavailability 20%; ^2^ The human oral bioavailability 30%; ^3^ The human oral bioavailability 50%; ^4^ Plasma clearance; ^5^ The half-life of molecule.

## Data Availability

The original contributions presented in this study are included in the article/Supplementary Materials. Further inquiries can be directed to the corresponding authors.

## Supplementary Materials

The following supporting information can be downloaded at: https://www.mdpi.com/article/10.3390/molecules30214150/s1.
