# Supplementary material for: New Dihalogenated Derivatives of Condensed Benzimidazole Diones Promotes Cancer Cell Death Through Regulating STAT3/HK2 Axis/Pathway
Source: Molecules. 2025 Oct 22;30(21):4150. doi: 10.3390/molecules30214150 (PMC12610816; doi:10.3390/molecules30214150)
Supplement: Supplementary file 1 [file molecules-30-04150-s001.zip › molecules-3911882-supplementary.pdf]

Supplementary information

New Dihalogenated Derivatives of Condensed Benzimidazole Diones Promotes Cancer Cell Death through Regulating STAT3/HK2 Axis/Pathway

Yulia Aleksandrova[1,2], Luiza Savina[1], Inna Shagina[1,2], Anna Lyubina[3], Alla Zubishina[1], Svetlana Makarova[1], Anna Bagylly[1], Alexander Khokhlov[1], Roman Begunov[1,*] and Margarita Neganova[1,2,*]

[1] Institute of Pharmacy, Yaroslavl State Medical University of the Ministry of Health of the Russian Federation, Yaroslavl 150000, Russia
[2] Nesmeyanov Institute of Organoelement Compounds, Russian Academy of Sciences, Moscow 119991, Russia
[3] Arbuzov Institute of Organic and Physical Chemistry, FRC Kazan Scientific Center, Russian Academy of Sciences, Kazan 420088, Russia
* Correspondence: begunov@uniyar.ac.ru (R.B.); neganova83@mail.ru (M.N.)

Table of Contents:

1. ^{1}H and ^{13}C NMR spectra of compounds **2a-2d** S2-S5
2. ^{1}H and ^{13}C NMR spectra of compounds **3a-3f** S6-S11
3. ^{1}H and ^{13}C NMR spectra of compounds **4a, 4'a, 4c and 4d** S12-S16
4. ^{1}H and ^{13}C NMR spectra of compounds **5c, 5d** S17-S18
5. ^{1}H and ^{13}C NMR spectra of compounds **6a-6f** S19-S24
6. Table "In silico prediction of cytotoxicity of new heterocyclic quinones **6a-6f**" S25-S26.

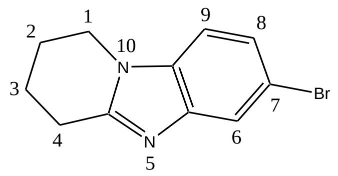

2a

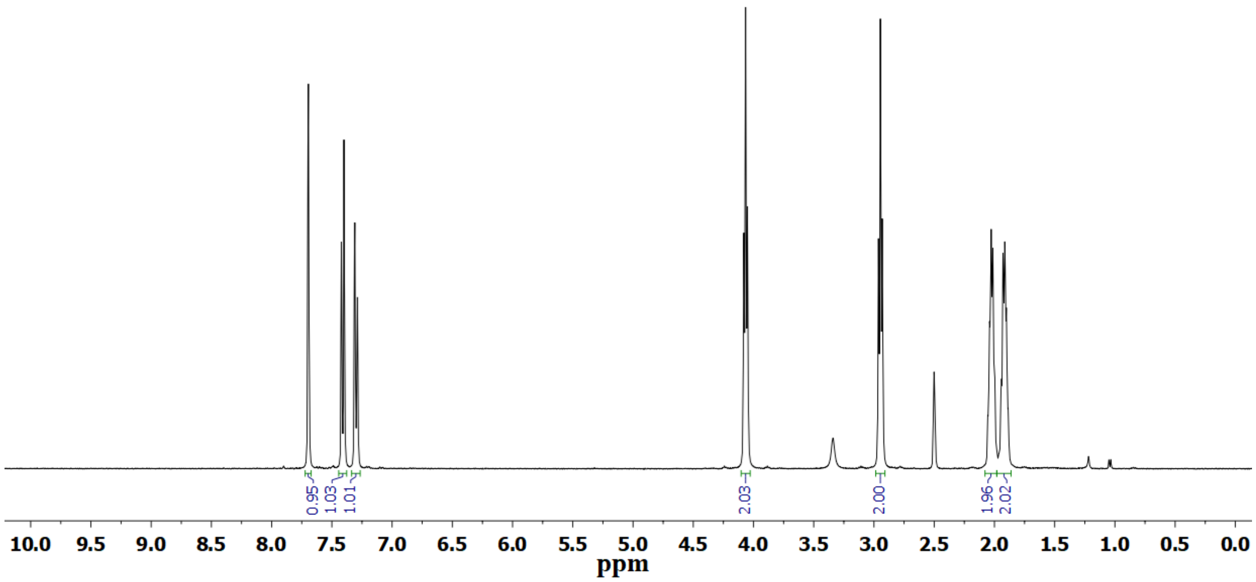

^{1}H NMR spectra of 7-bromo-1,2,3,4-tetrahydropyrido[1,2-*a*]benzimidazole **(2a)** (DMSO-*d*6)

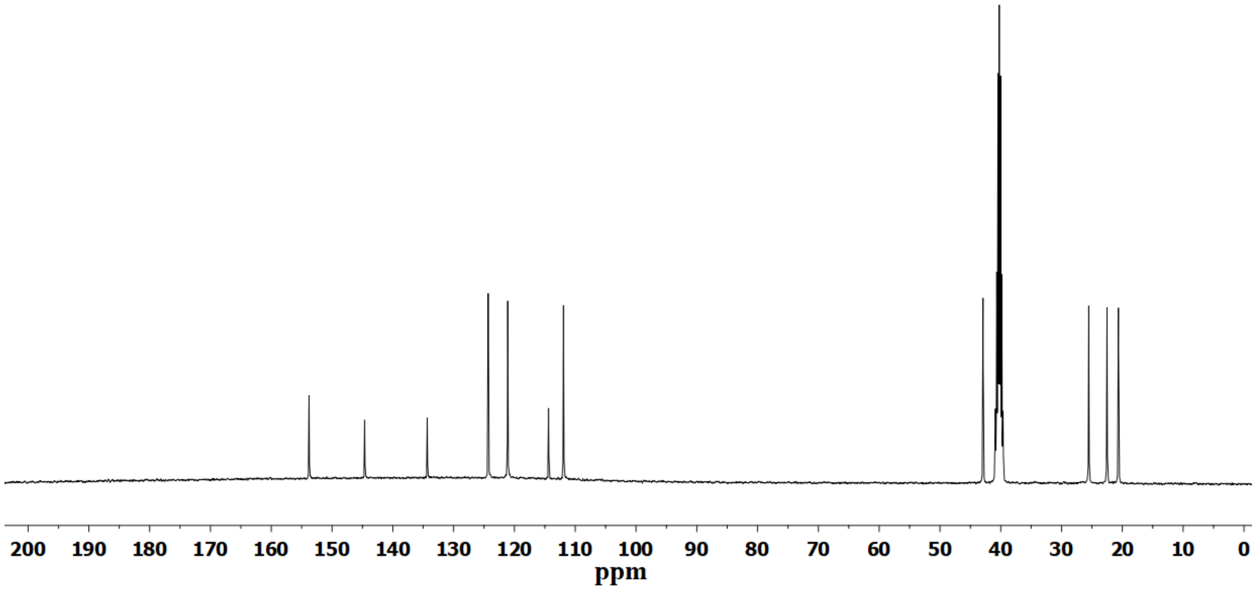

^{13}C NMR spectra of 7-bromo-1,2,3,4-tetrahydropyrido[1,2-*a*]benzimidazole **(2a)** (DMSO-*d*6)

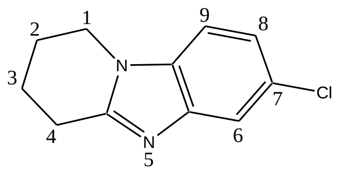

2b

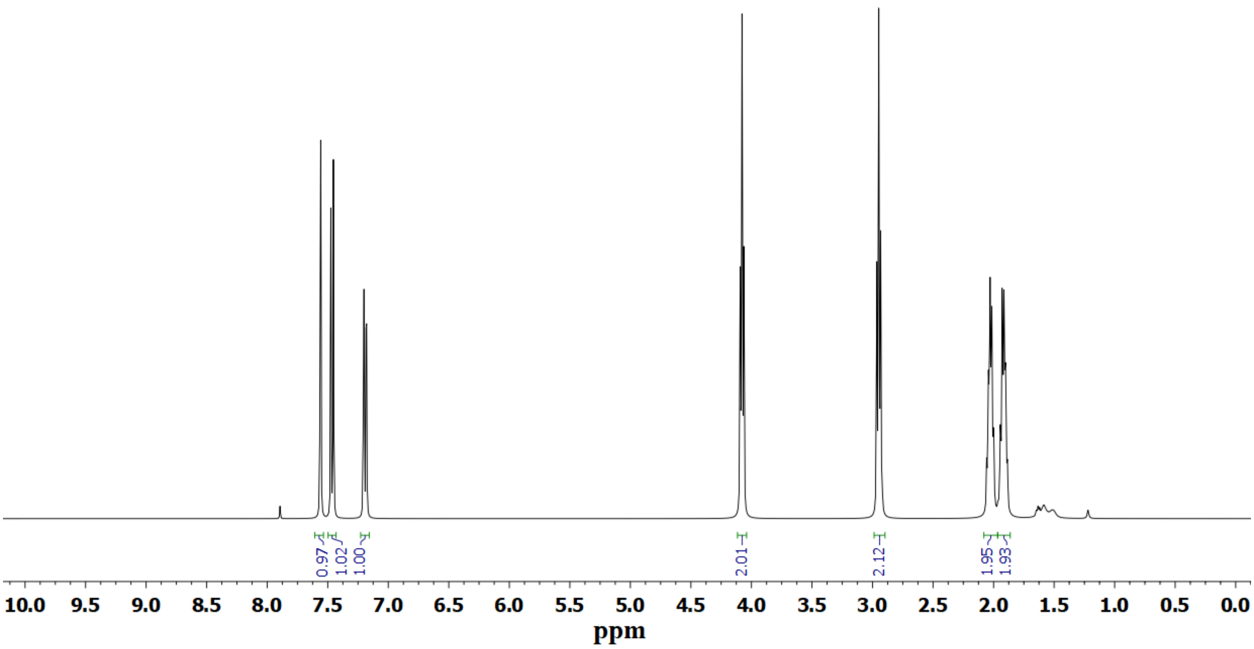

^{1}H NMR spectra of 7-chloro-1,2,3,4-tetrahydropyrido[1,2-*a*]benzimidazole **(2b)** (DMSO-*d*6)

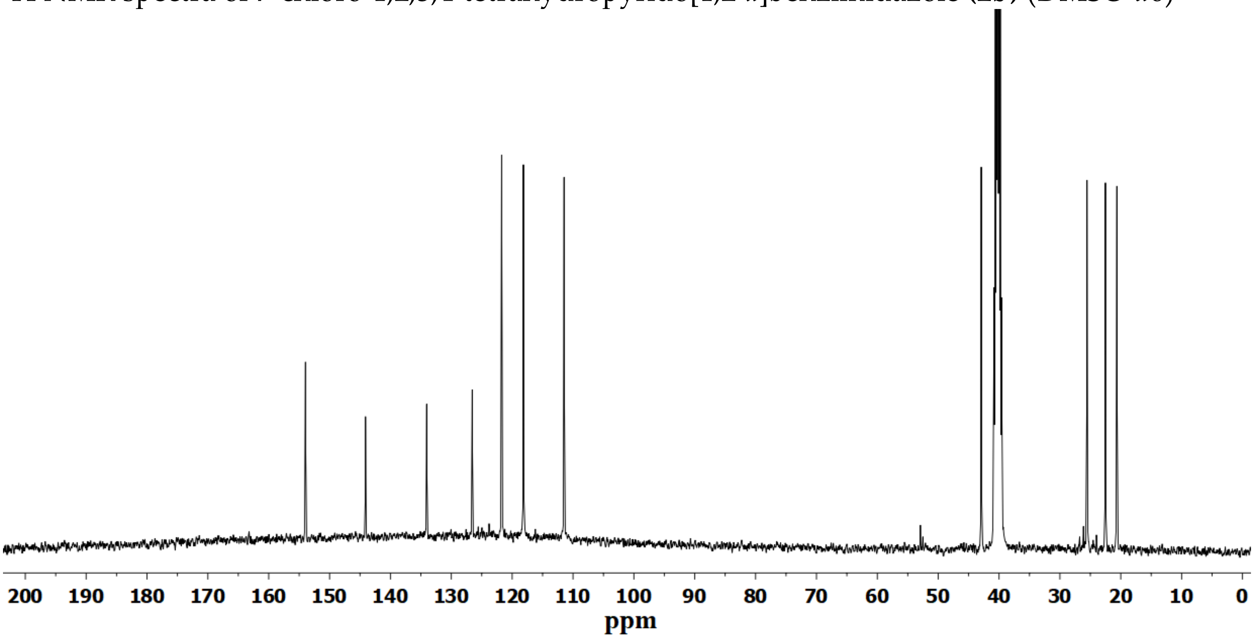

^{13}C NMR spectra of 7-chloro-1,2,3,4-tetrahydropyrido[1,2-*a*]benzimidazole **(2b)** (DMSO-*d*6)

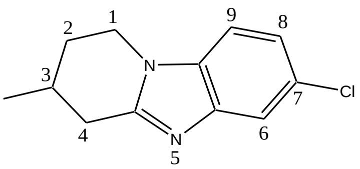

2c

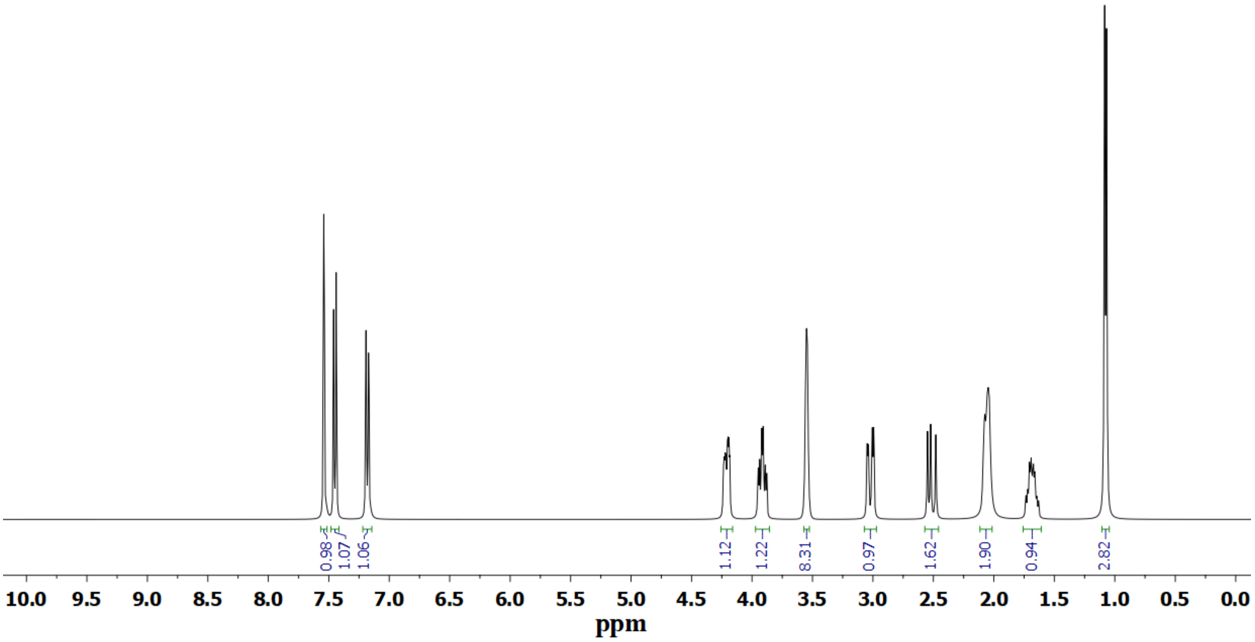

^{1}H NMR spectra of 7-chloro-3-methyl-1,2,3,4-tetrahydropyrido[1,2-*a*]benzimidazole **(2c)** (DMSO-*d*6)

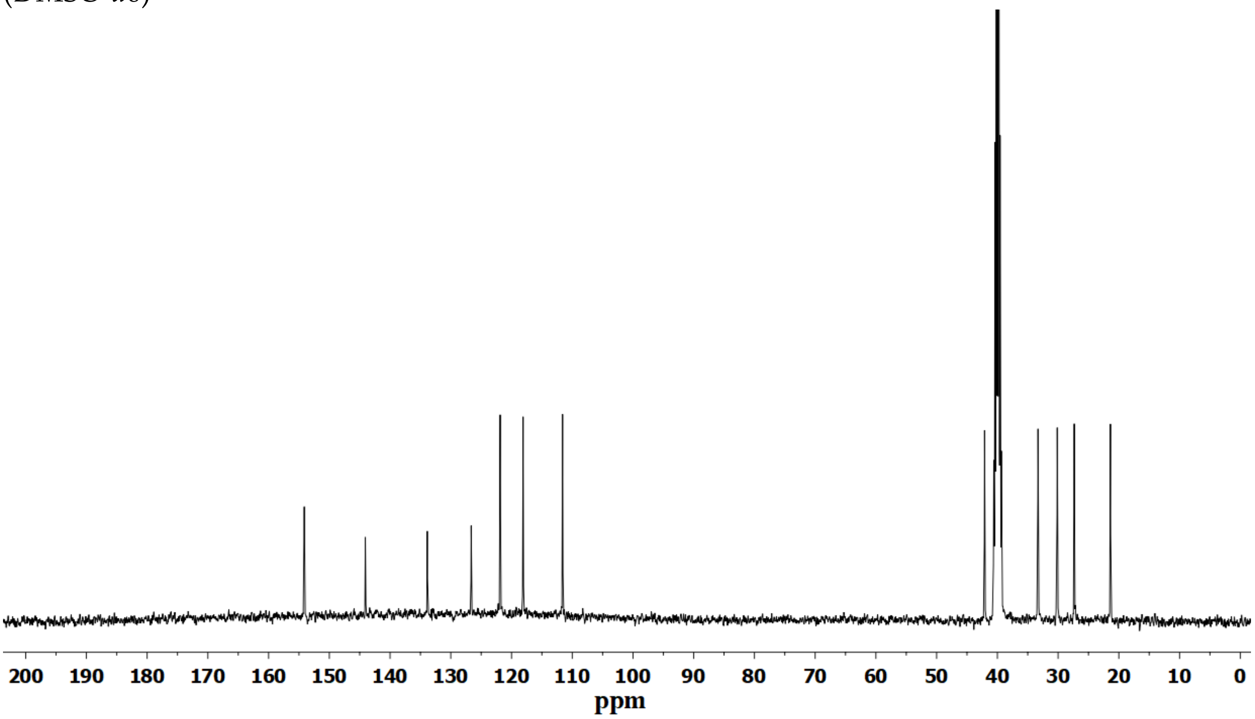

^{13}C NMR spectra of 7-chloro-3-methyl-1,2,3,4-tetrahydropyrido[1,2-*a*]benzimidazole **(2c)** (DMSO-*d*6)

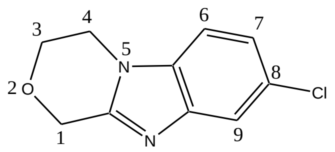

2d

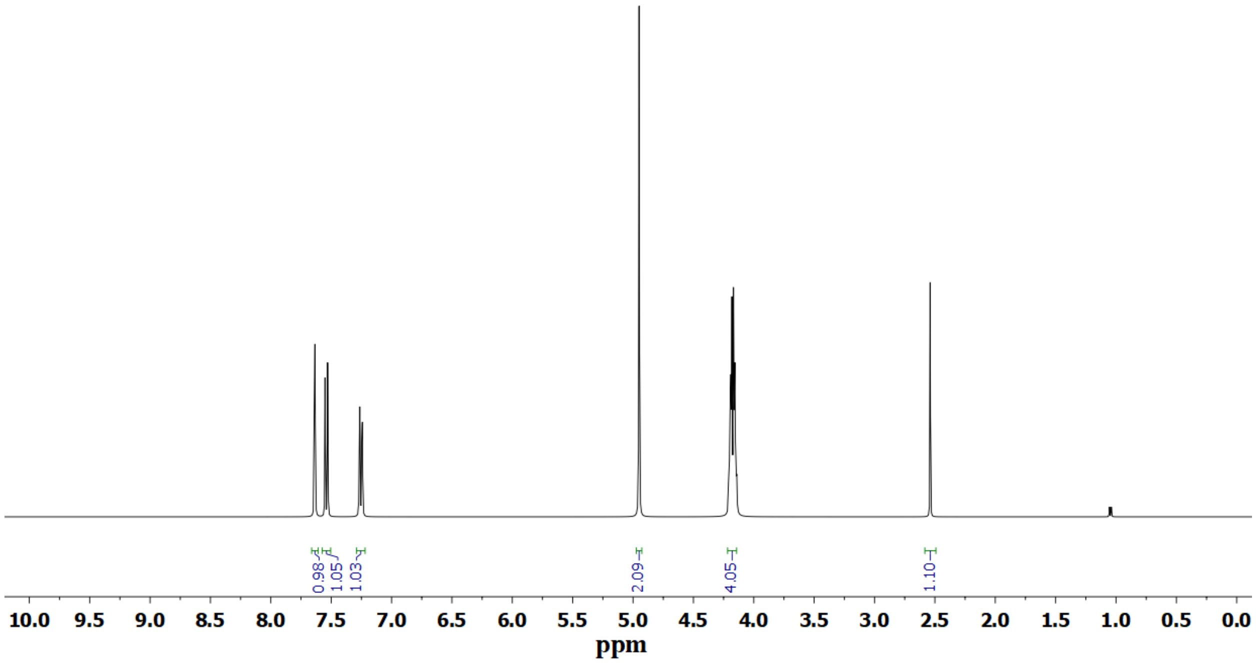

^{1}H NMR spectra of 8-chloro-3,4-dihydro-1*H*-[1,4]oxazino[4,3-*a*]benzimidazole **(2d)** (DMSO-*d*6)

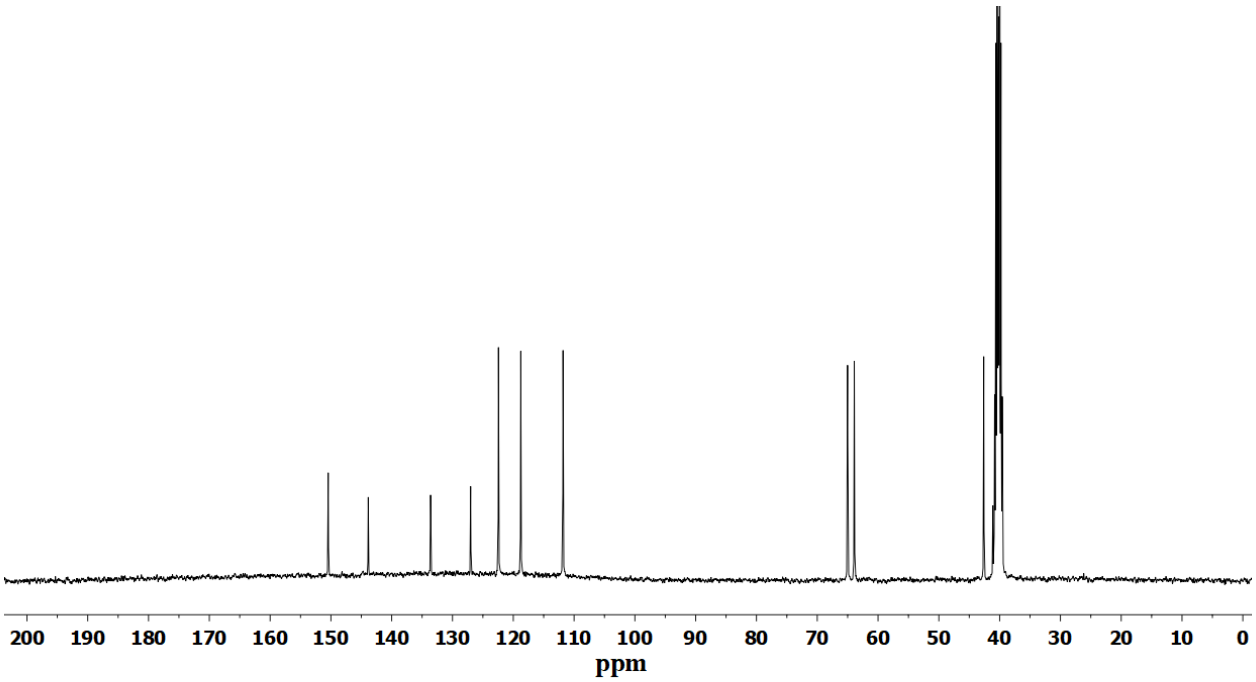

^{13}C NMR spectra of 8-chloro-3,4-dihydro-1*H*-[1,4]oxazino[4,3-*a*]benzimidazole **(2d)** (DMSO-*d*6)

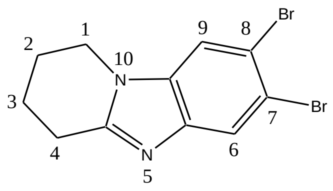

3a

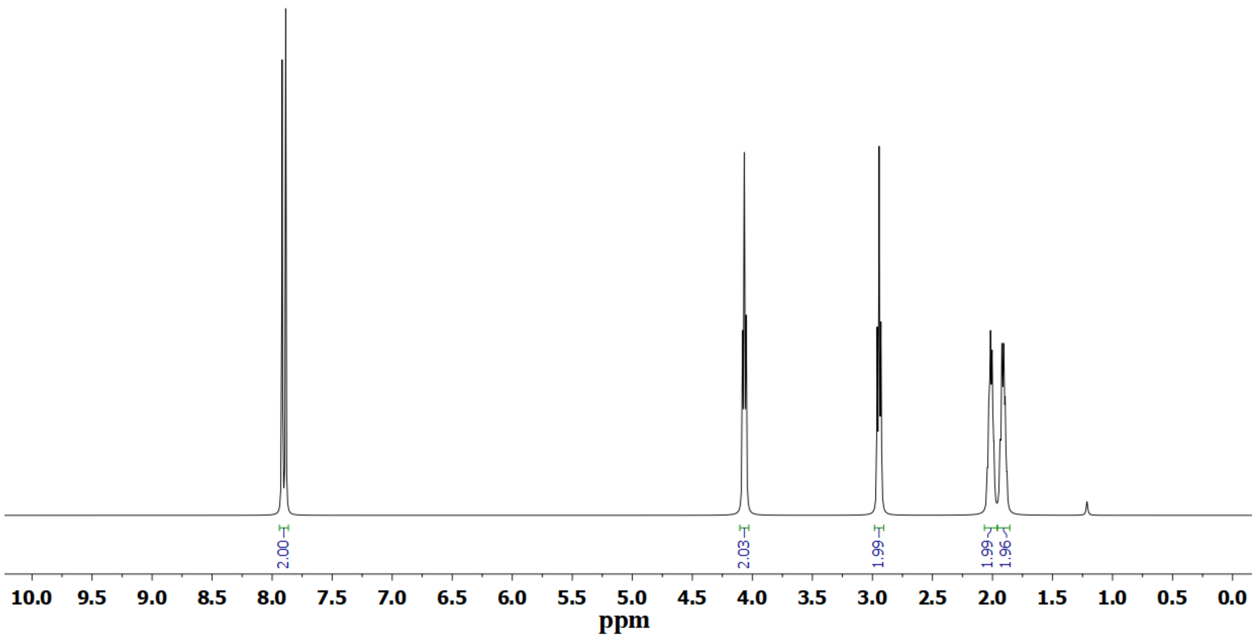

^{1}H NMR spectra of 7,8-dibromo-1,2,3,4-tetrahydropyrido[1,2-*a*]benzimidazole **(3a)** (DMSO-*d*6)

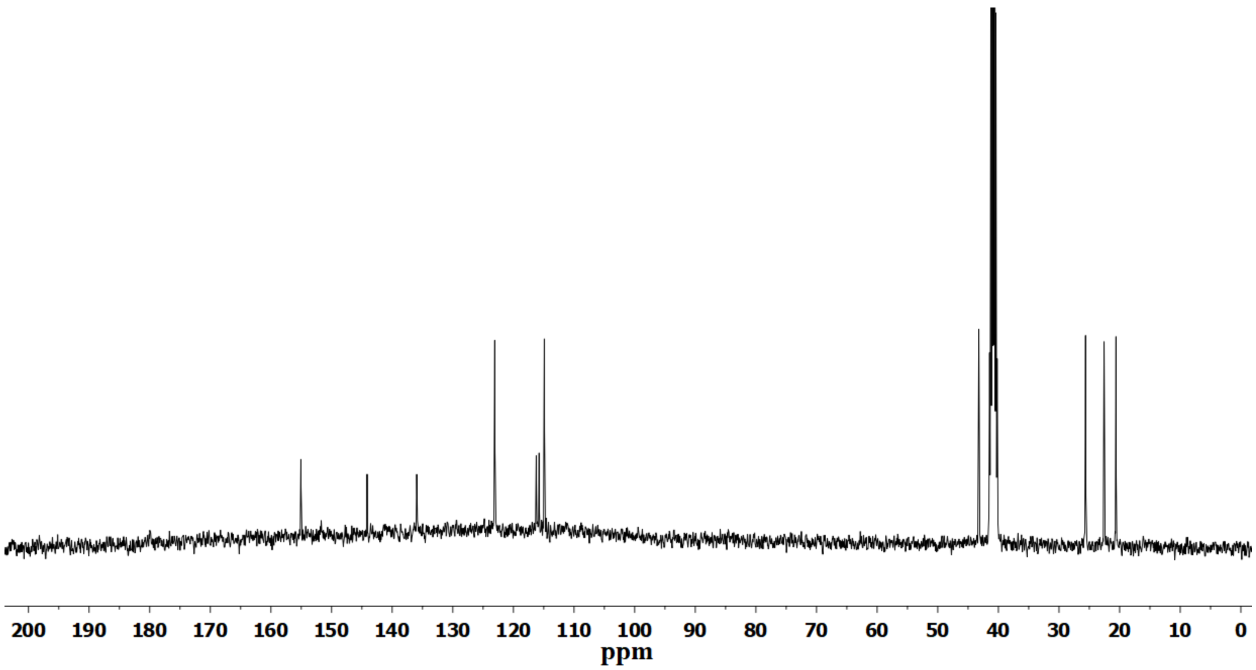

^{13}C NMR spectra of 7,8-dibromo-1,2,3,4-tetrahydropyrido[1,2-*a*]benzimidazole **(3a)** (DMSO-*d*6)

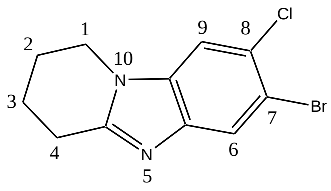

3b

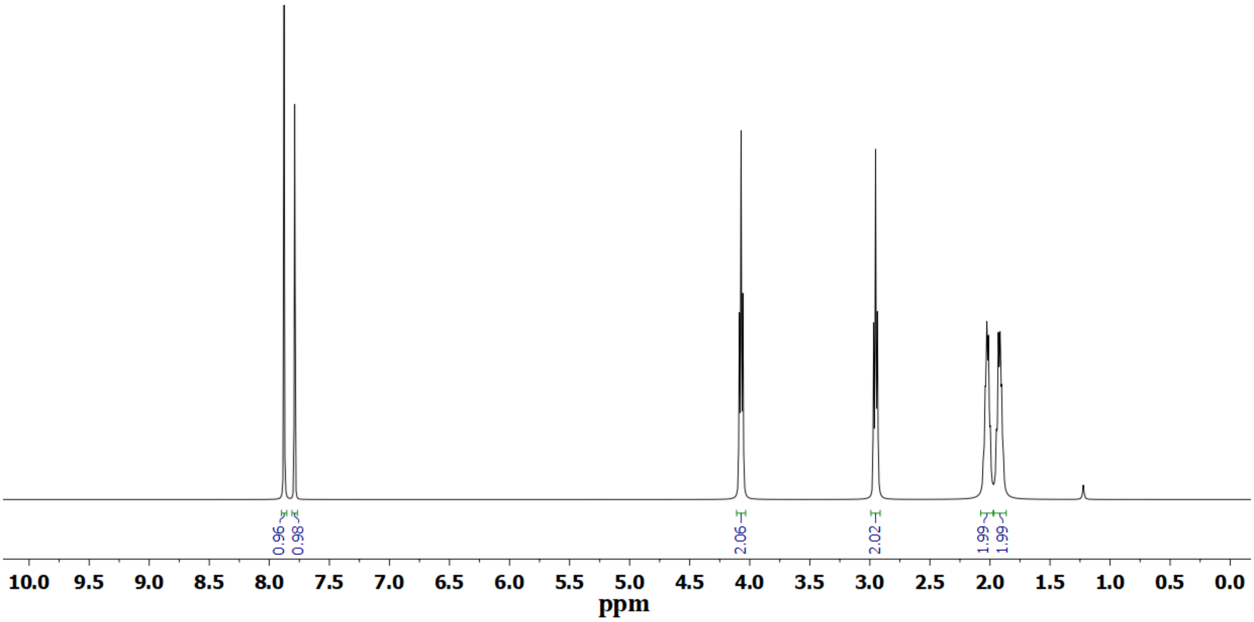

^{1}H NMR spectra of 7-bromo-8-chloro-1,2,3,4-tetrahydropyrido[1,2-*a*]benzimidazole **(3b)** (DMSO-*d*6)

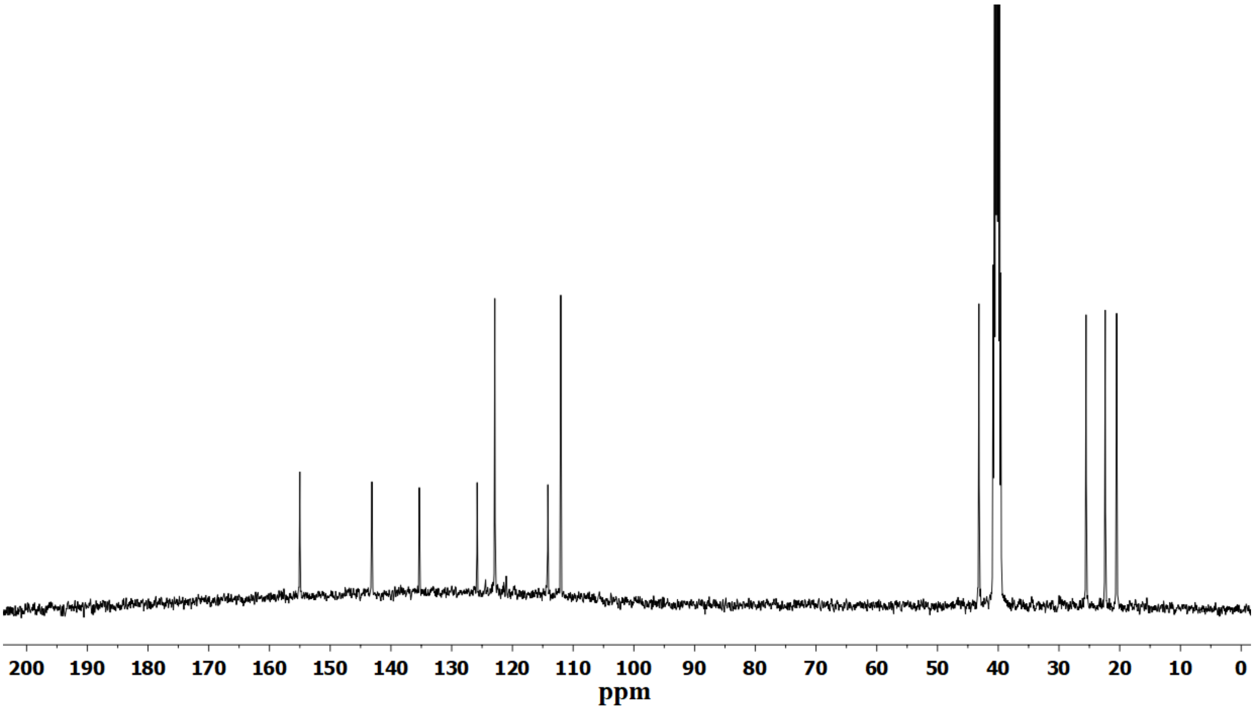

^{13}C NMR spectra of 7-bromo-8-chloro-1,2,3,4-tetrahydropyrido[1,2-*a*]benzimidazole **(3b)** (DMSO-*d*6)

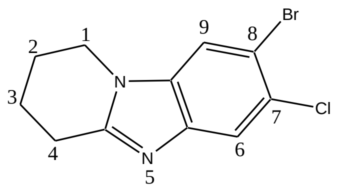

3c

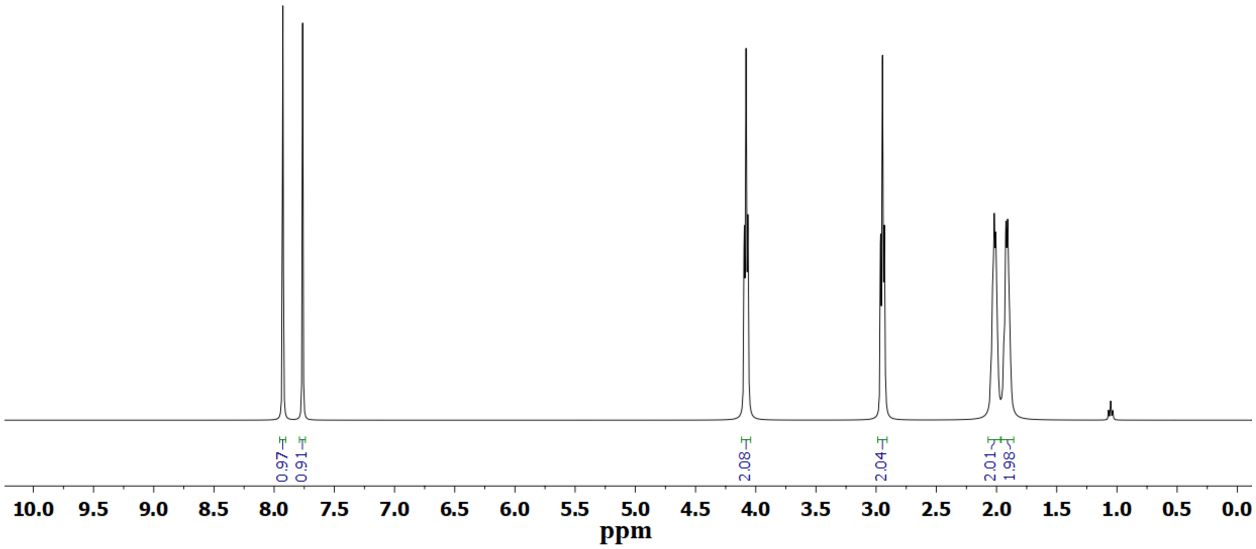

^{1}H NMR spectra of 8-bromo-7-chloro-1,2,3,4-tetrahydropyrido[1,2-*a*]benzimidazole **(3c)** (DMSO-*d*6)

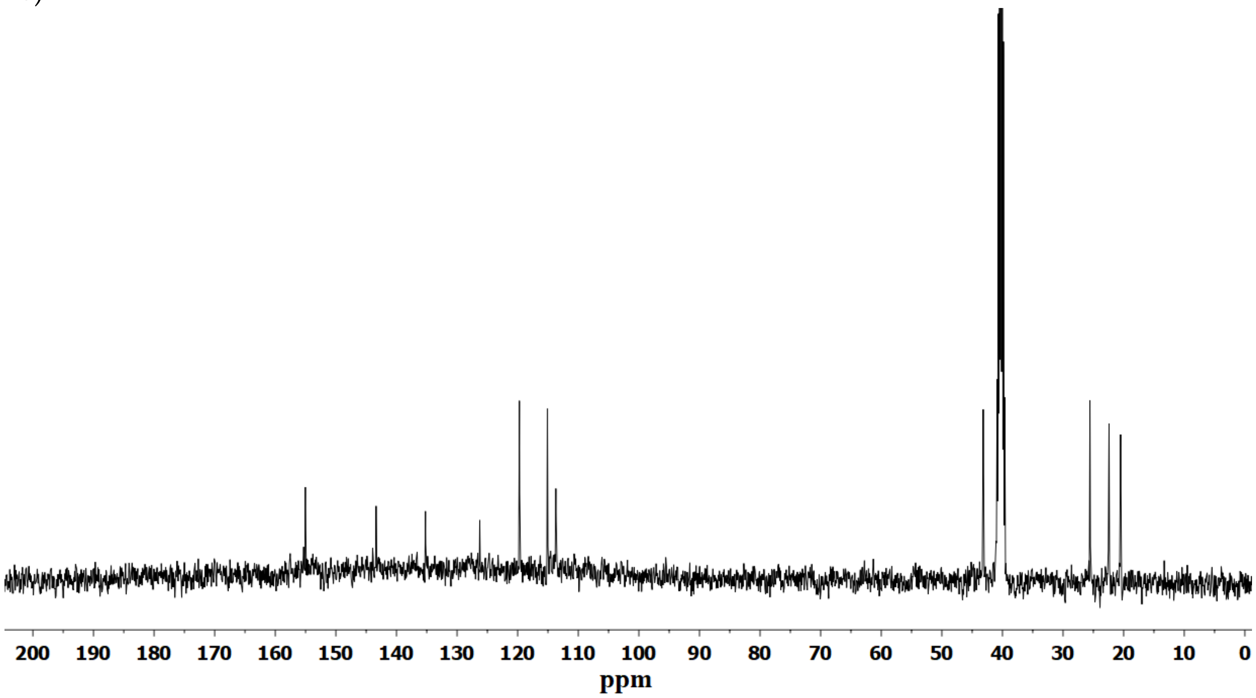

^{13}C NMR spectra of 8-bromo-7-chloro-1,2,3,4-tetrahydropyrido[1,2-*a*]benzimidazole **(3c)** (DMSO-*d*6)

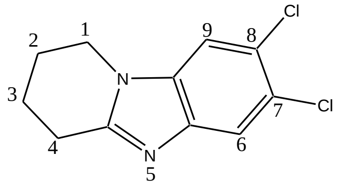

3d

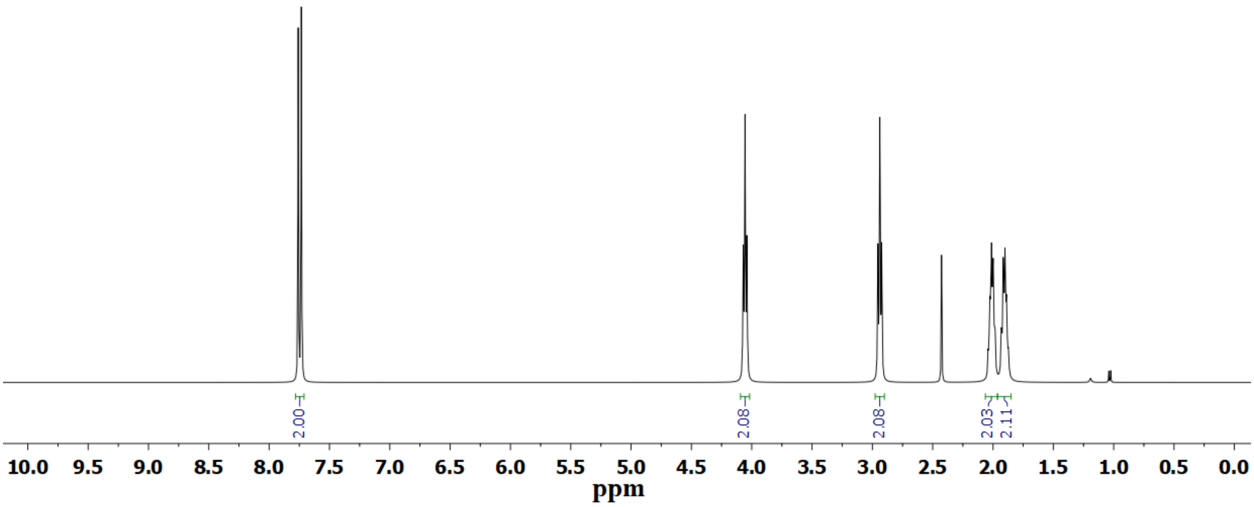

^{1}H NMR spectra of 7,8-dichloro-1,2,3,4-tetrahydropyrido[1,2-*a*]benzimidazole **(3d)** (DMSO-*d*6)

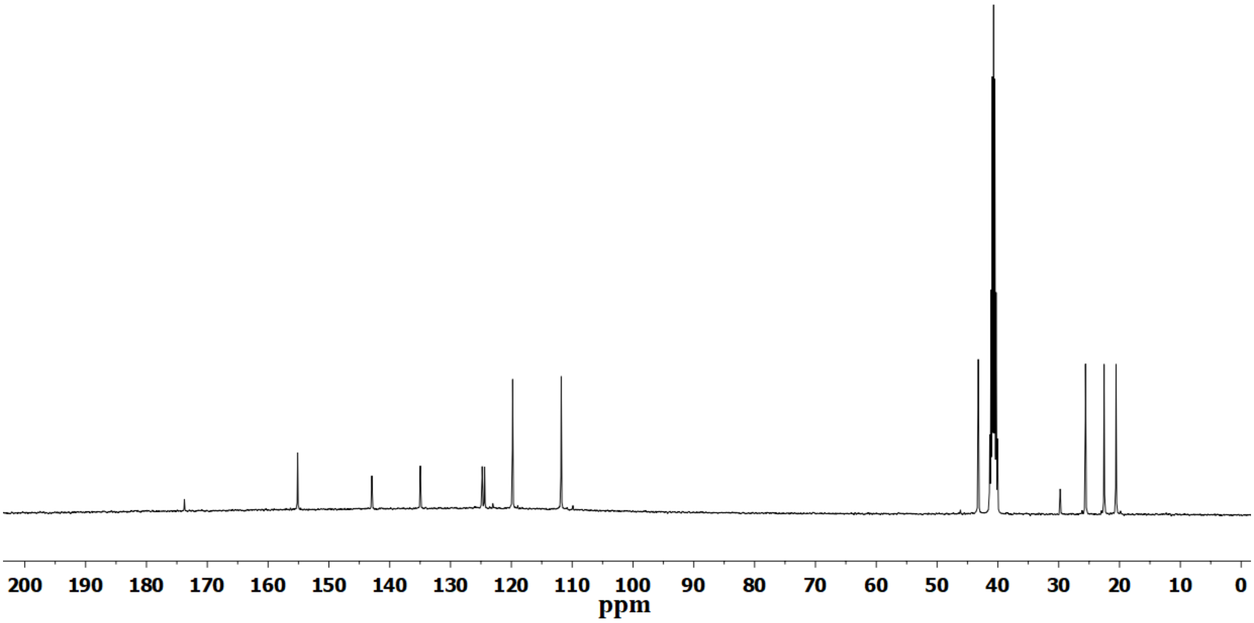

^{13}C NMR spectra of 7,8-dichloro-1,2,3,4-tetrahydropyrido[1,2-*a*]benzimidazole **(3d)** (DMSO-*d*6))

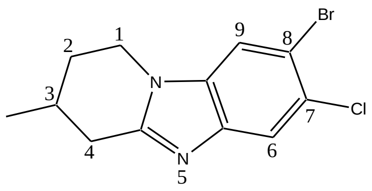

3e

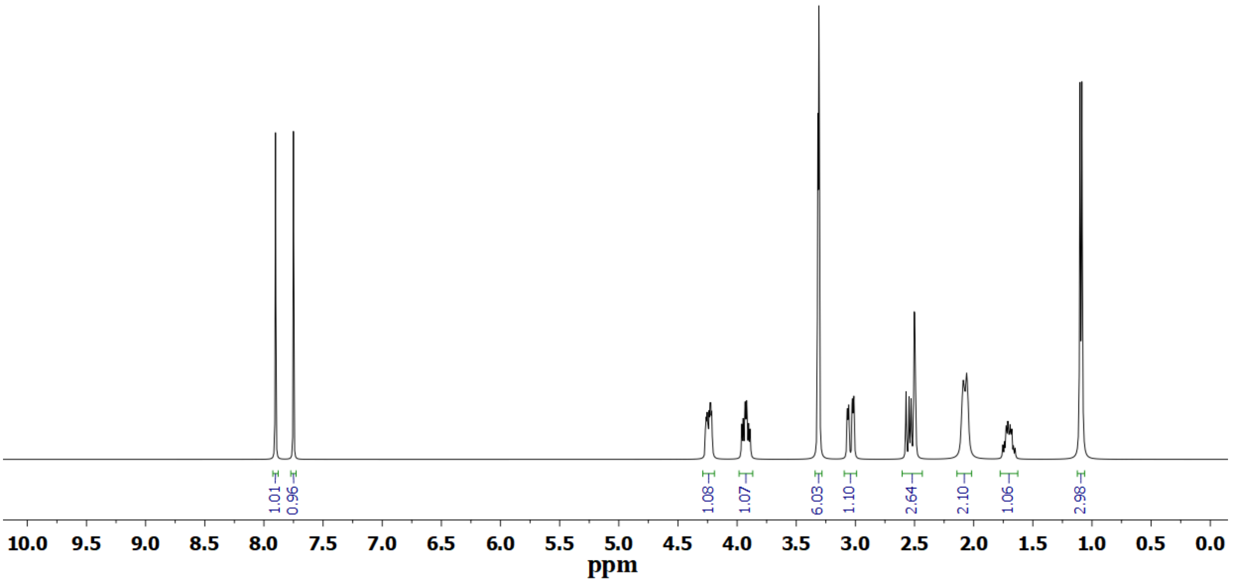

^{1}H NMR spectra of 8-bromo-7-chloro-3-methyl-1,2,3,4-tetrahydropyrido[1,2-*a*]benzimidazole **(3e)** (DMSO-*d*6)

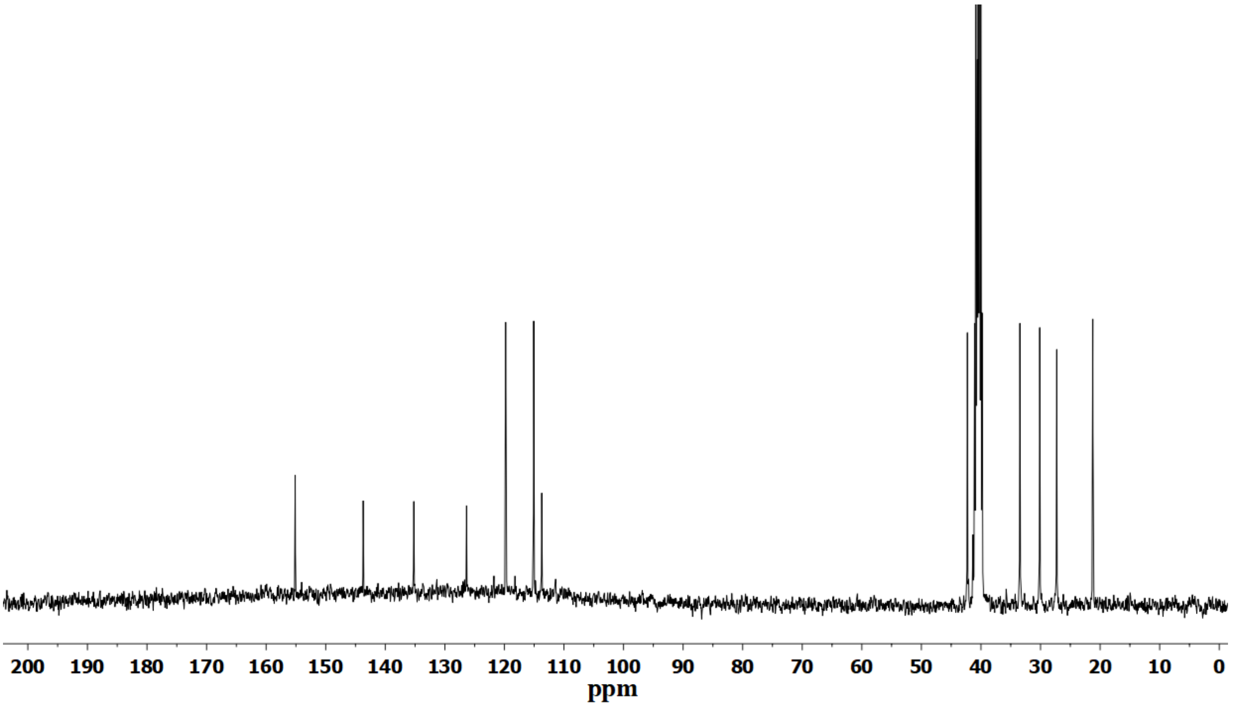

^{13}C NMR spectra of 8-bromo-7-chloro-3-methyl-1,2,3,4-tetrahydropyrido[1,2-*a*]benzimidazole **(3e)** (DMSO-*d*6)

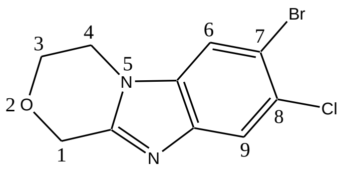

3f

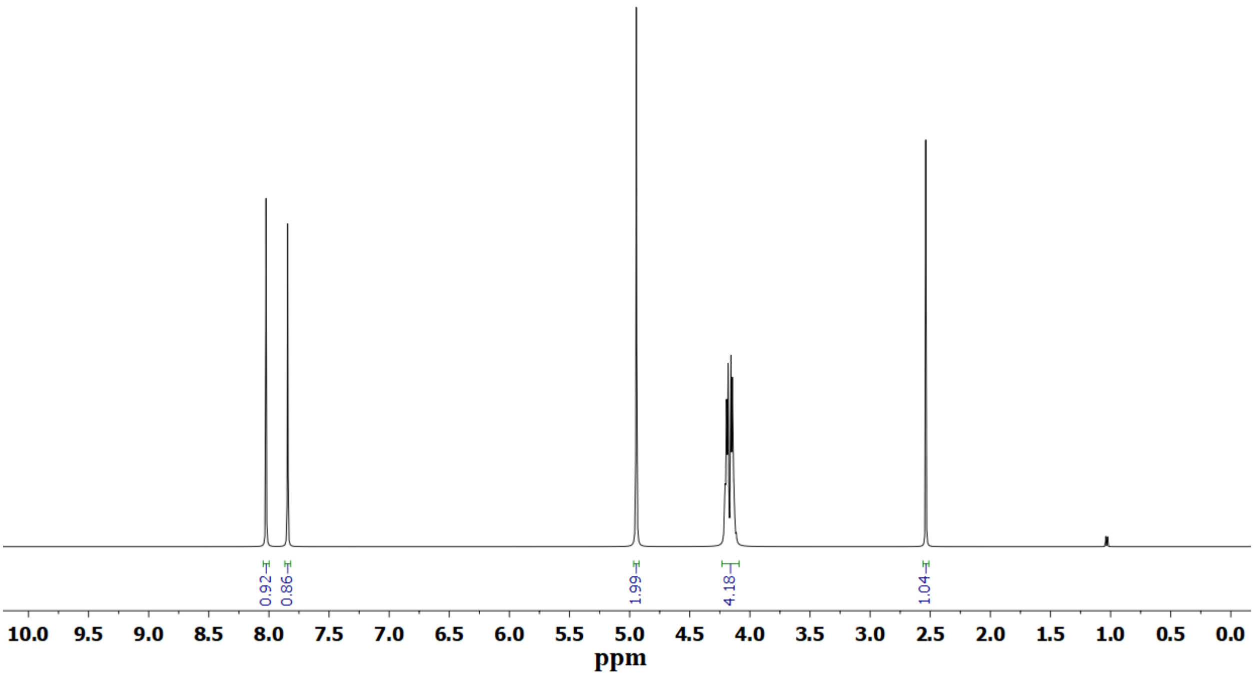

^{1}H NMR spectra of 7-bromo-8-chloro-3,4-dihydro-1*H*-[1,4]oxazino[4,3-*a*]benzimidazole **(3f)** (DMSO-*d*6)

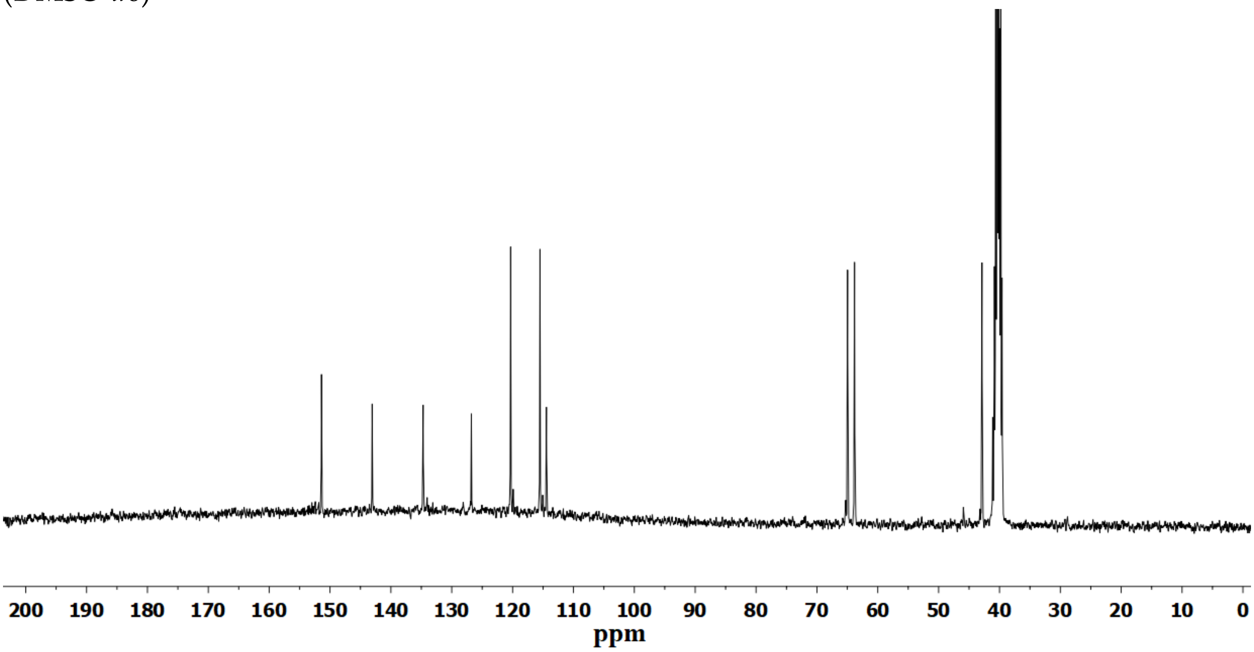

^{13}C NMR spectra of 7-bromo-8-chloro-3,4-dihydro-1*H*-[1,4]oxazino[4,3-*a*]benzimidazole **(3f)** (DMSO-*d*6)

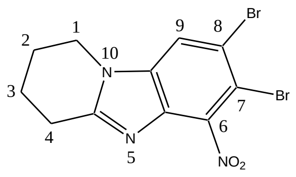

4a +

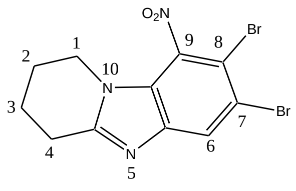

4'a

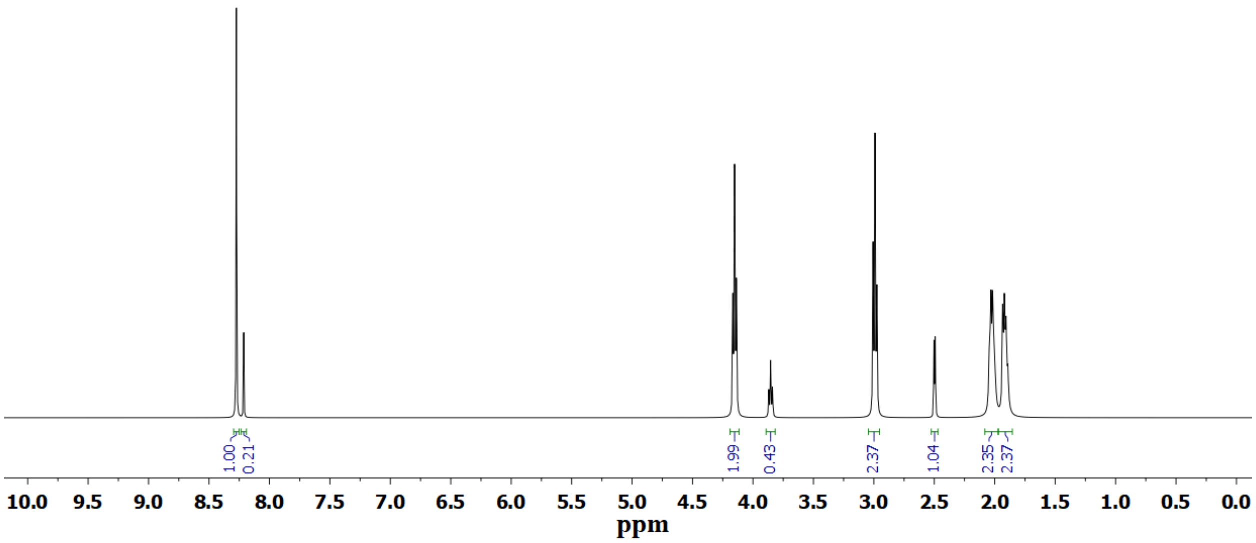

^1H NMR spectra of 7,8-dibromo-1,2,3,4-tetrahydropyrido[1,2-*a*]benzimidazole **(3a)** nitration reaction products **4a** and **4'a** (DMSO-*d*6)

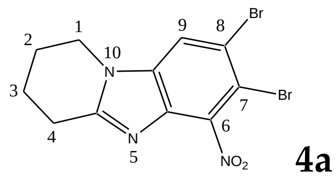

4a

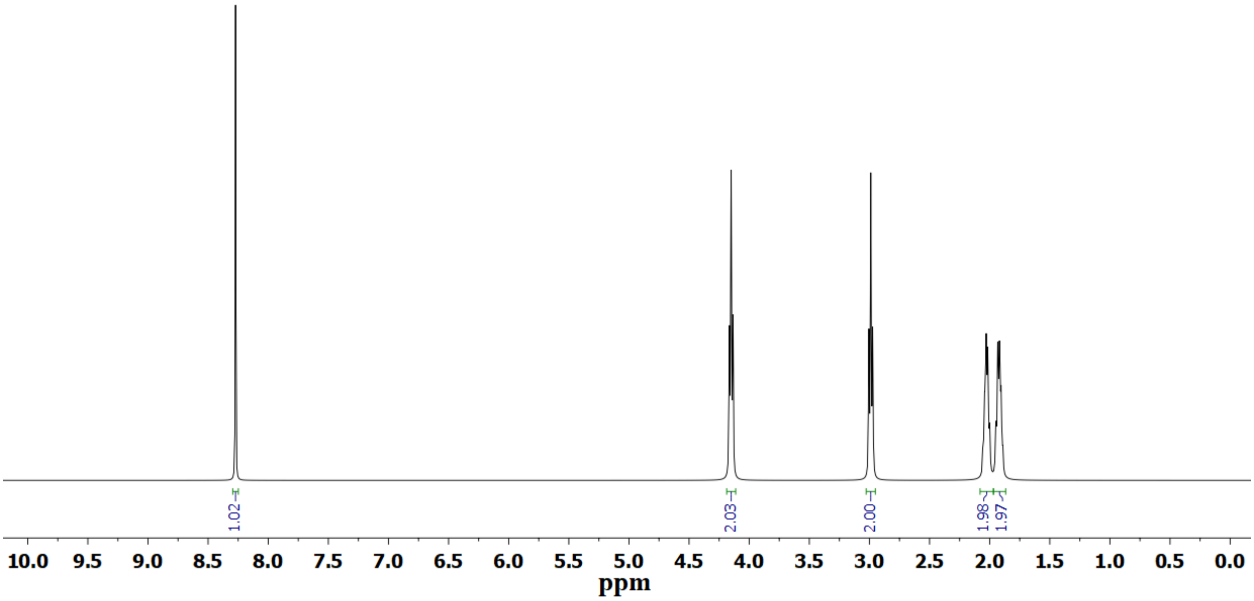

^{1}H NMR spectra of 7,8-dibromo-6-nitro-1,2,3,4-tetrahydropyrido[1,2-*a*]benzimidazole **(4a)** (DMSO-*d*6)

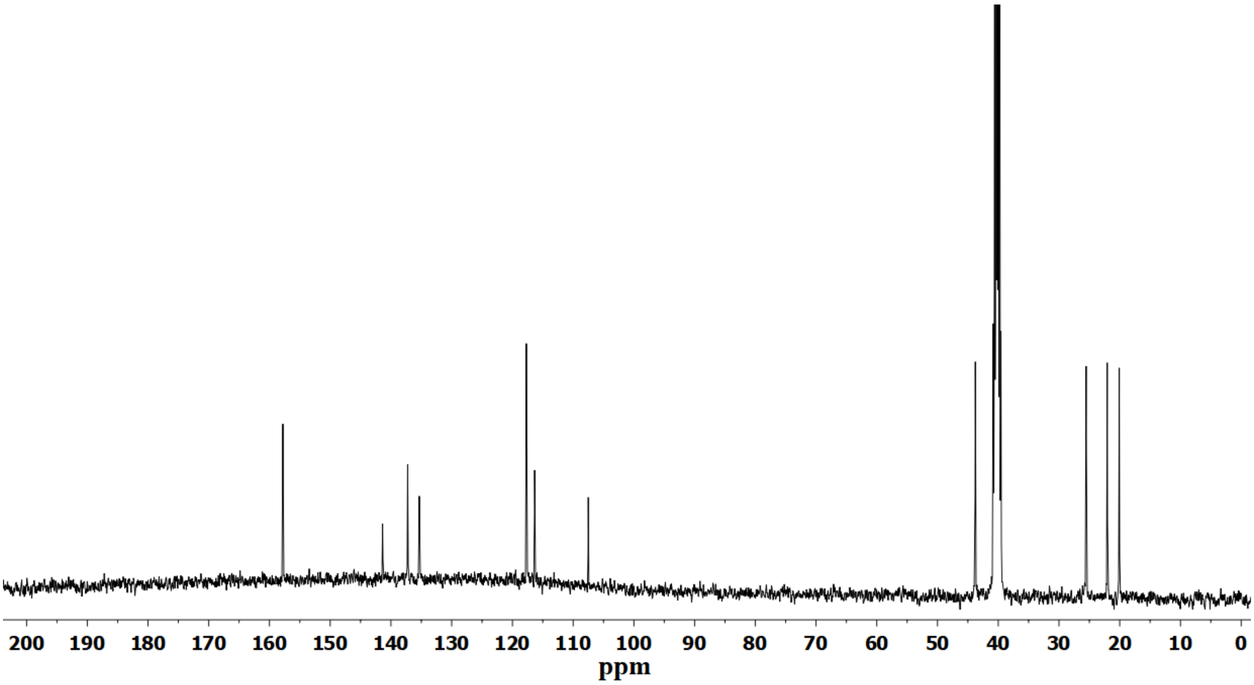

^{13}C NMR spectra of 7,8-dibromo-6-nitro-1,2,3,4-tetrahydropyrido[1,2-*a*]benzimidazole **(4a)** (DMSO-*d*6)

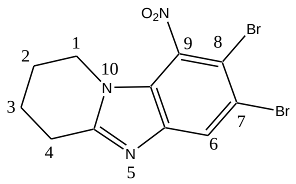

4'a

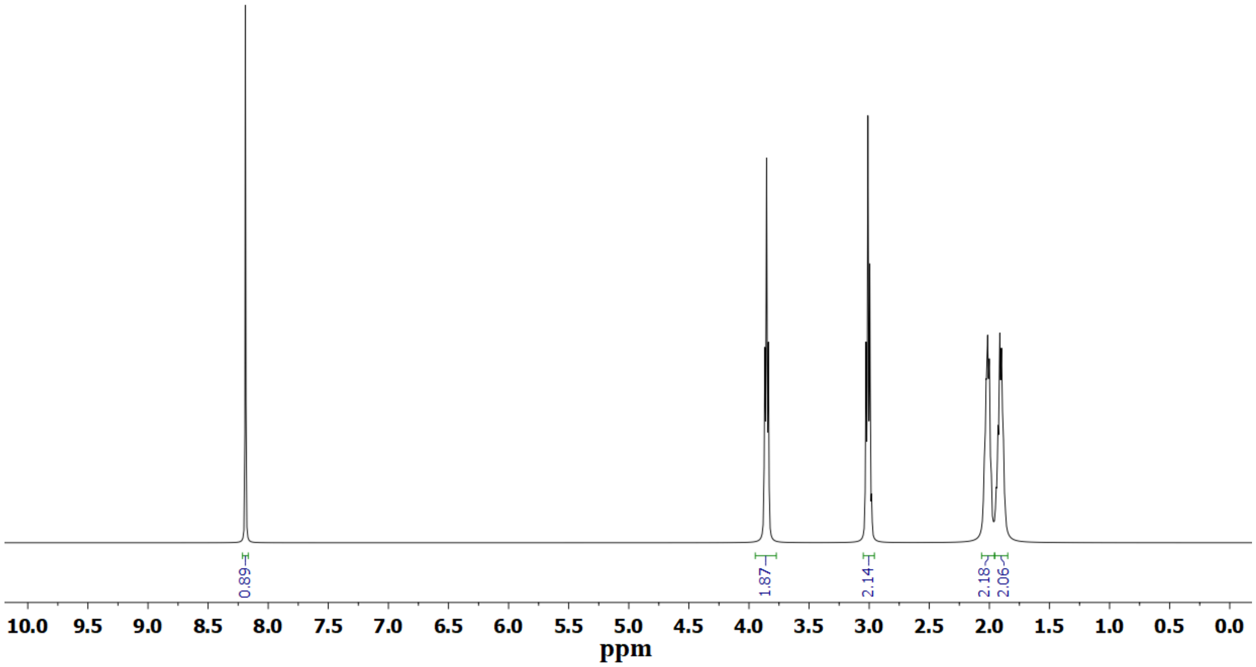

^{1}H NMR spectra of 7,8-dibromo-9-nitro-1,2,3,4-tetrahydropyrido[1,2-*a*]benzimidazole **(4’a)** (DMSO-*d*6)

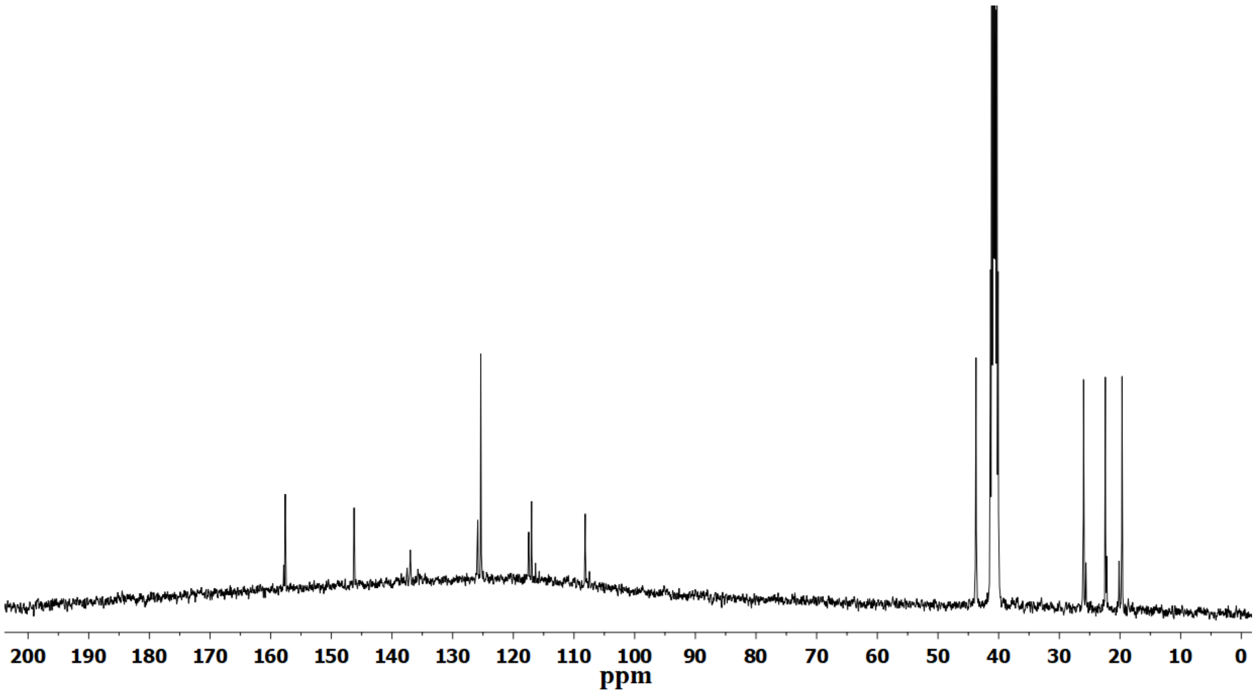

^{13}C NMR spectra of 7,8-dibromo-9-nitro-1,2,3,4-tetrahydropyrido[1,2-*a*]benzimidazole **(4’a)** (DMSO-*d*6)

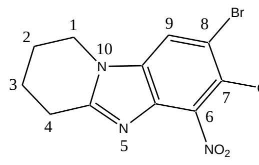

4c

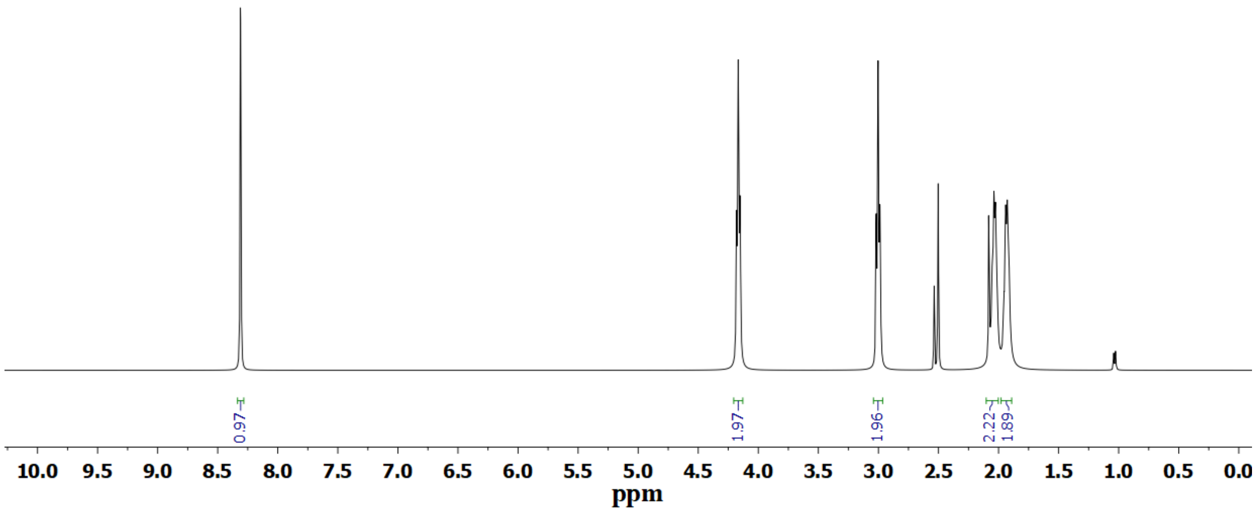

^{1}H NMR spectra of 8-bromo-7-chloro-6-nitro-1,2,3,4-tetrahydropyrido[1,2-*a*]benzimidazole **(4c)** (DMSO-*d*6)

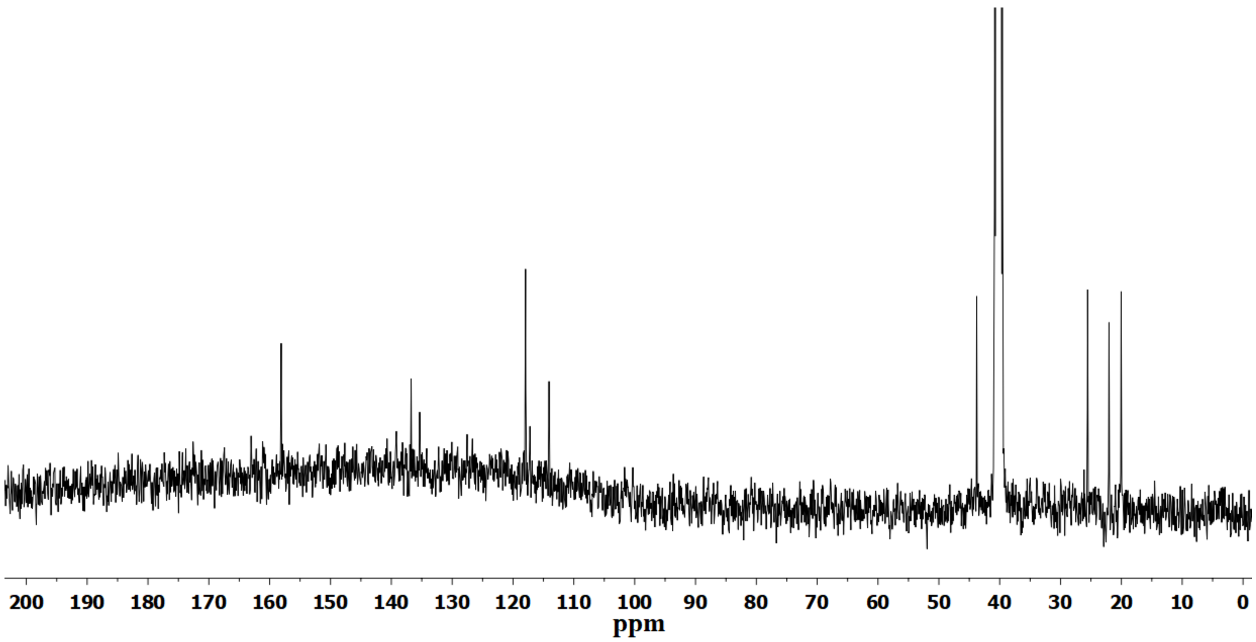

^{13}C NMR spectra of 8-bromo-7-chloro-6-nitro-1,2,3,4-tetrahydropyrido[1,2-*a*]benzimidazole **(4c)** (DMSO-*d*6)

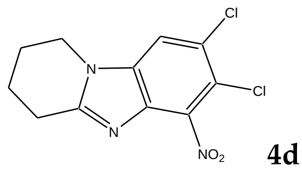

4d

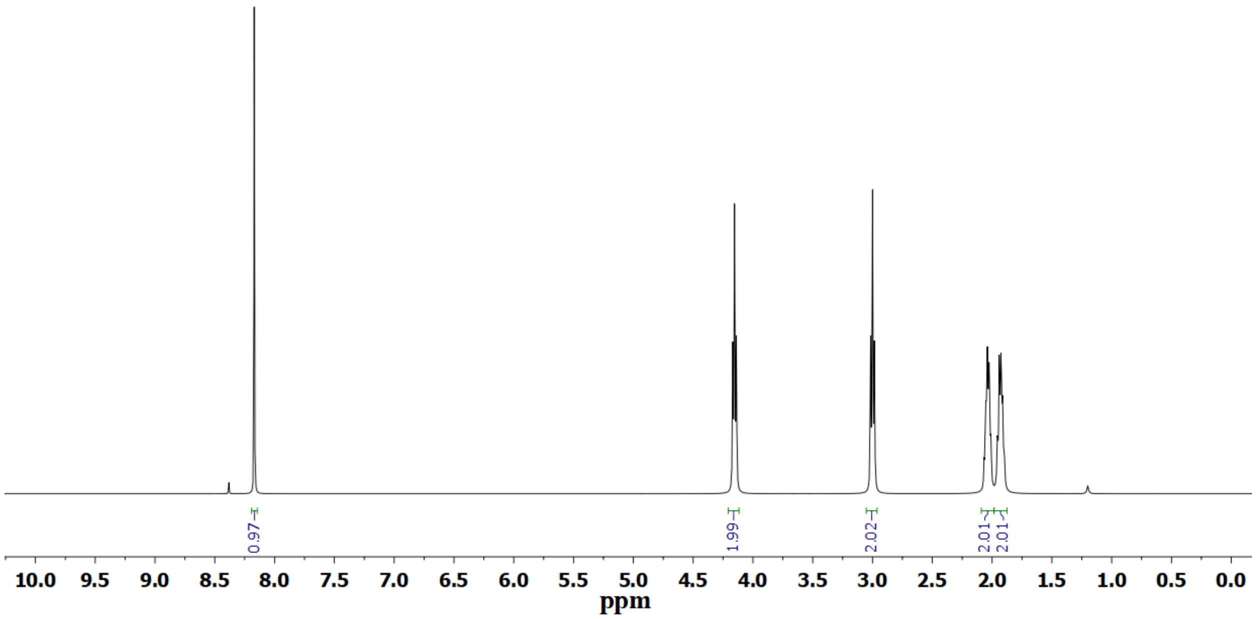

^{1}H NMR spectra of 7,8-dicloro-6-nitro-1,2,3,4-tetrahydropyrido[1,2-*a*]benzimidazole **(4d)** (DMSO-*d*6)

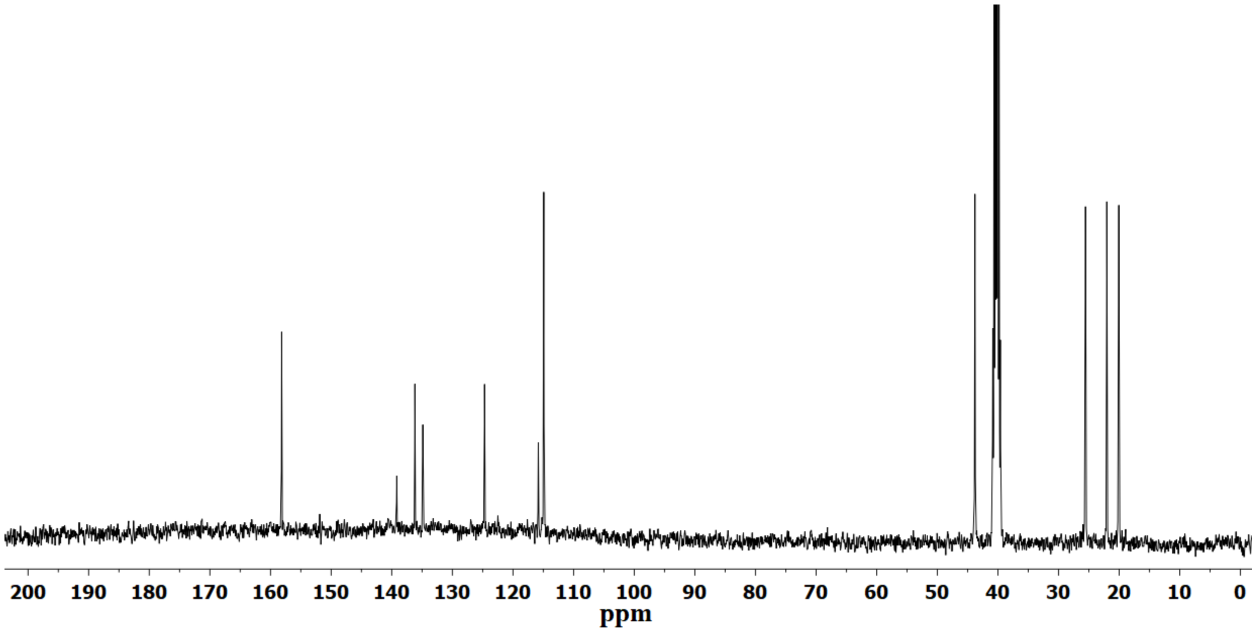

^{13}C NMR spectra of 7,8-dicloro-6-nitro-1,2,3,4-tetrahydropyrido[1,2-*a*]benzimidazole **(4d)** (DMSO-*d*6)

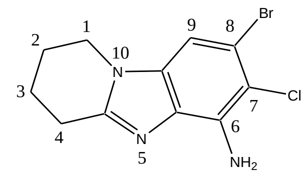

5c

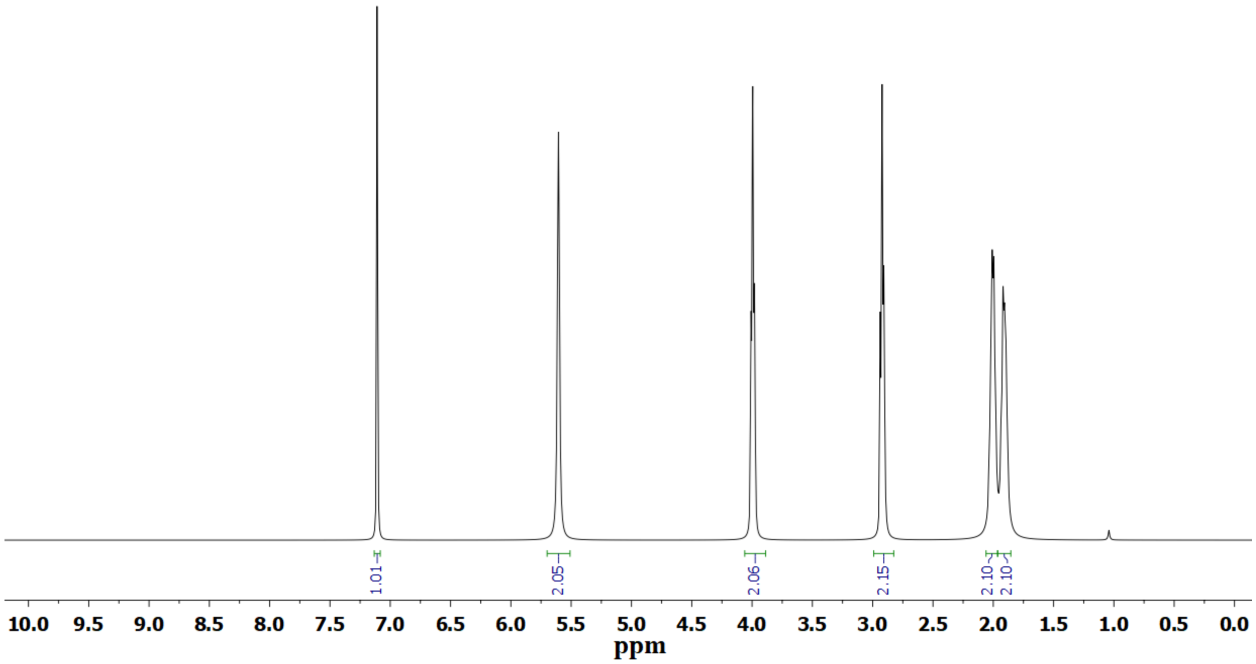

^{1}H NMR spectra of 8-bromo-7-chloro-1,2,3,4-tetrahydropyrido[1,2-*a*]benzimidazol-6-amine **(5c)** (DMSO-*d*6)

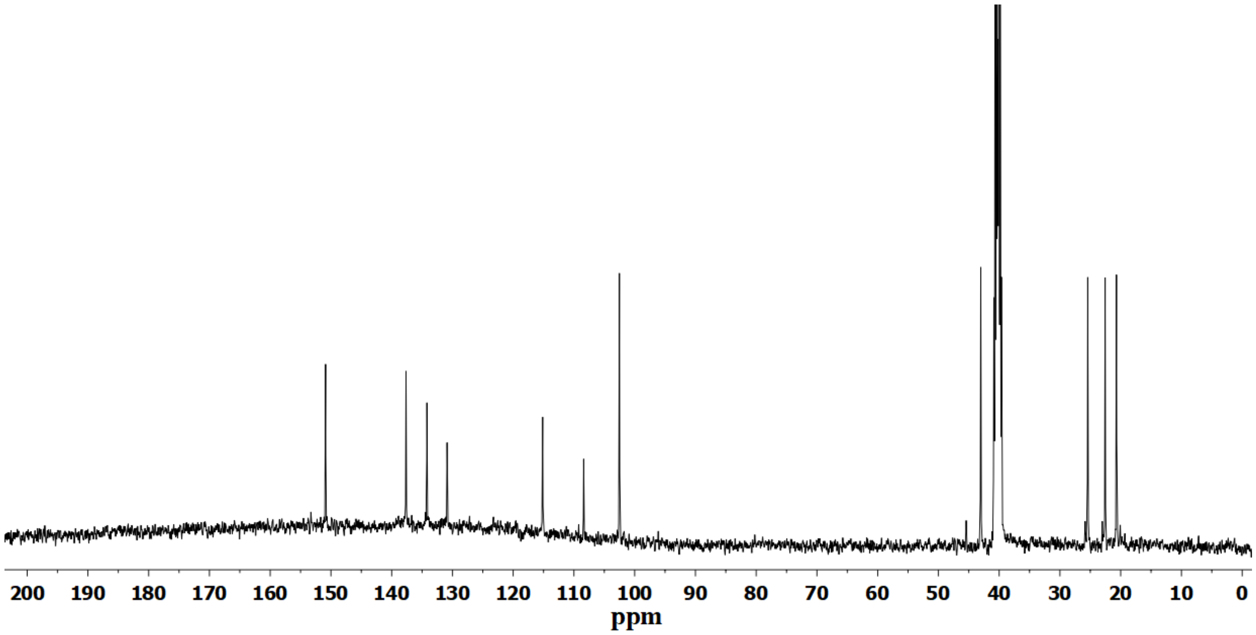

^{13}C NMR spectra of 8-bromo-7-chloro-1,2,3,4-tetrahydropyrido[1,2-*a*]benzimidazol-6-amine **(5c)** (DMSO-*d*6)

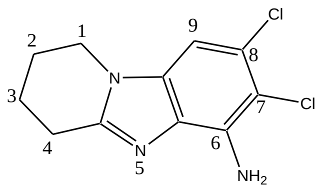

5d

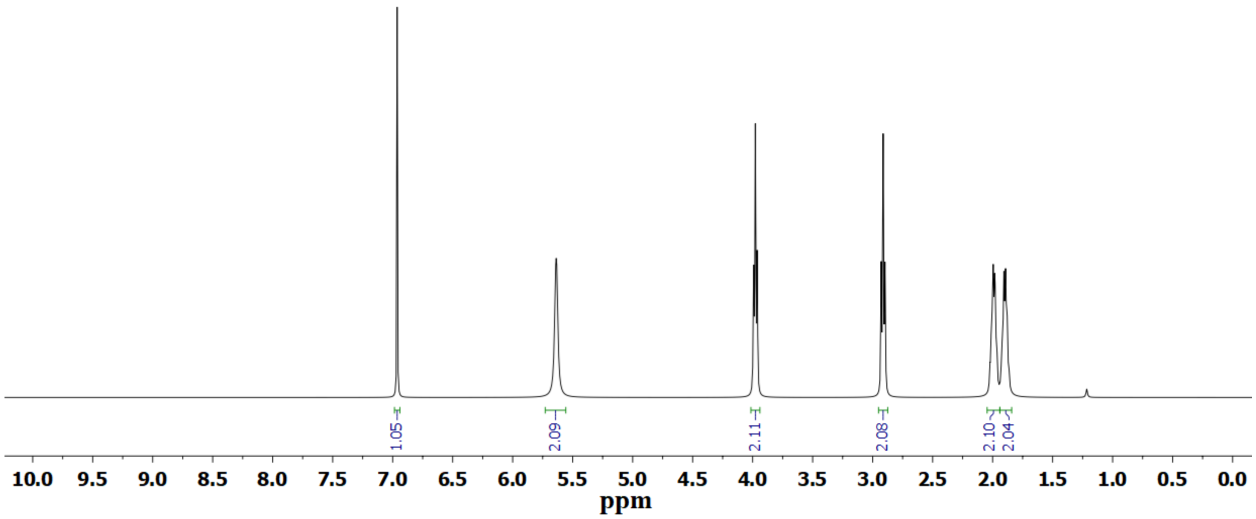

^{1}H NMR spectra of 7,8-dicloro-1,2,3,4-tetrahydropyrido[1,2-*a*]benzimidazole-6-amine **(5d)** (DMSO-*d*6)

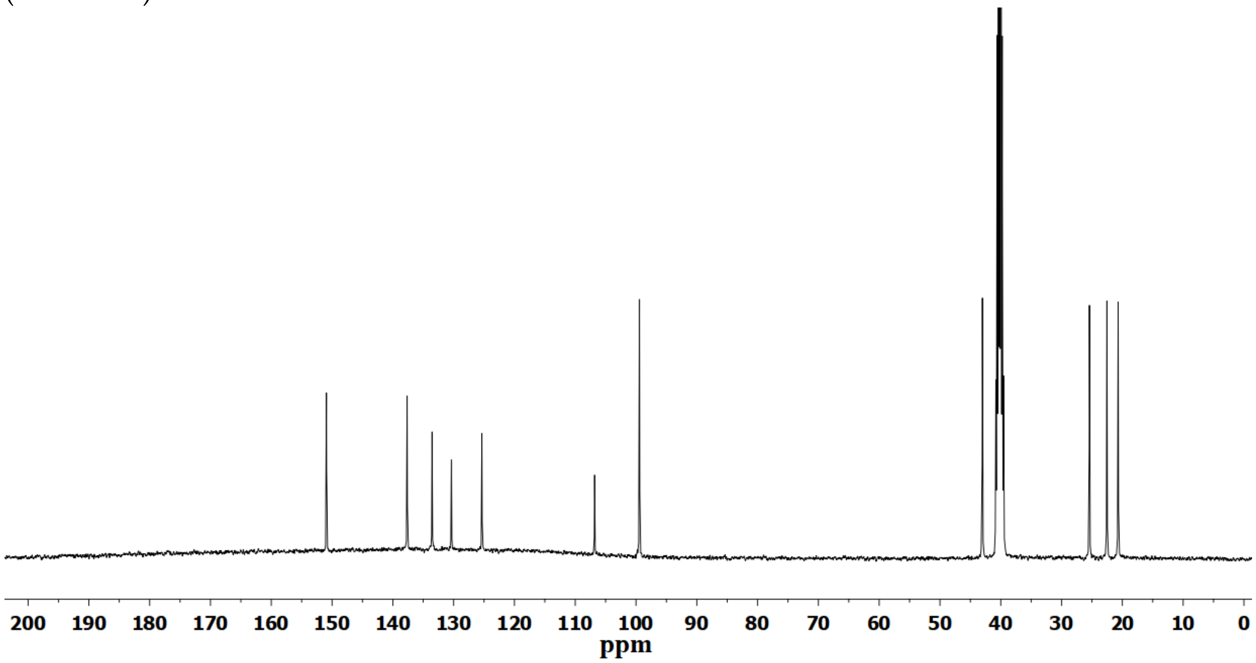

^{13}C NMR spectra of 7,8-dicloro-1,2,3,4-tetrahydropyrido[1,2-*a*]benzimidazole-6-amine **(5d)** (DMSO-*d*6)

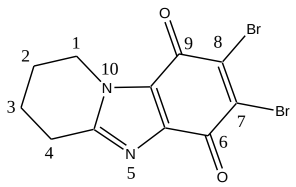

6a

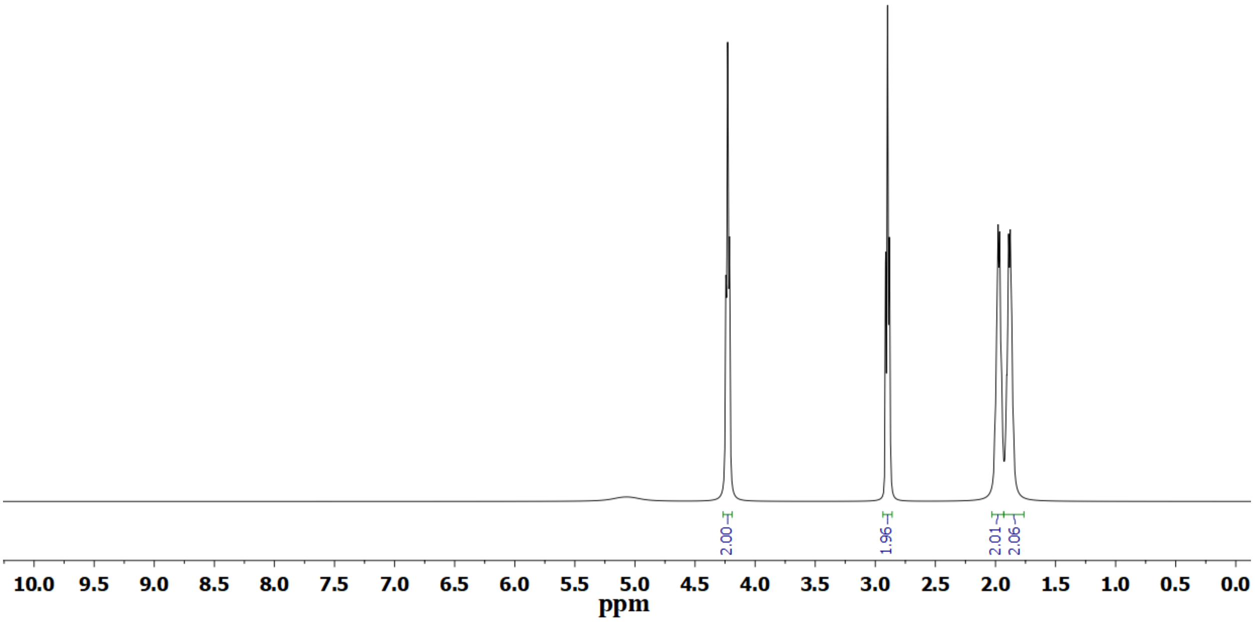

^{1}H NMR spectra of 7,8-dibromo-1,2,3,4-tetrahydropyrido[1,2-*a*]benzimidazole-6,9-dione **(6a)** (DMSO-*d*6)

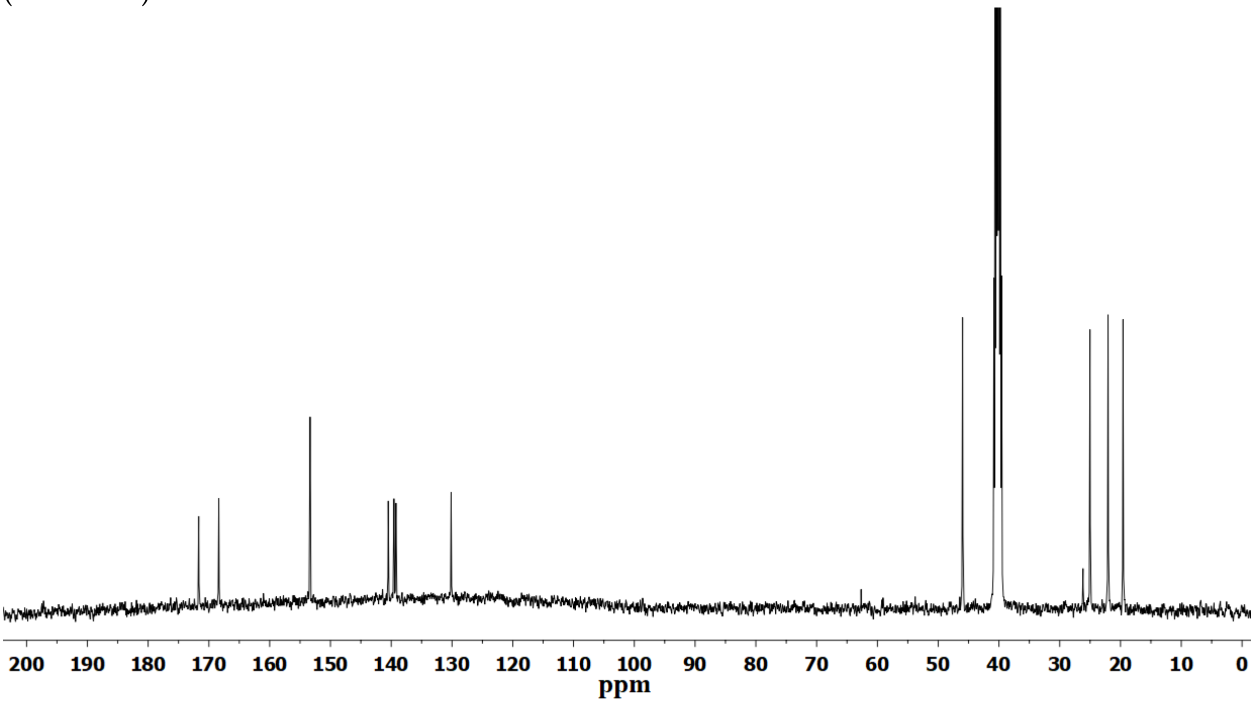

^{13}C NMR spectra of 7,8-dibromo-1,2,3,4-tetrahydropyrido[1,2-*a*]benzimidazole-6,9-dione **(6a)** (DMSO-*d*6)

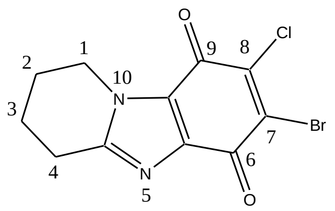

6b

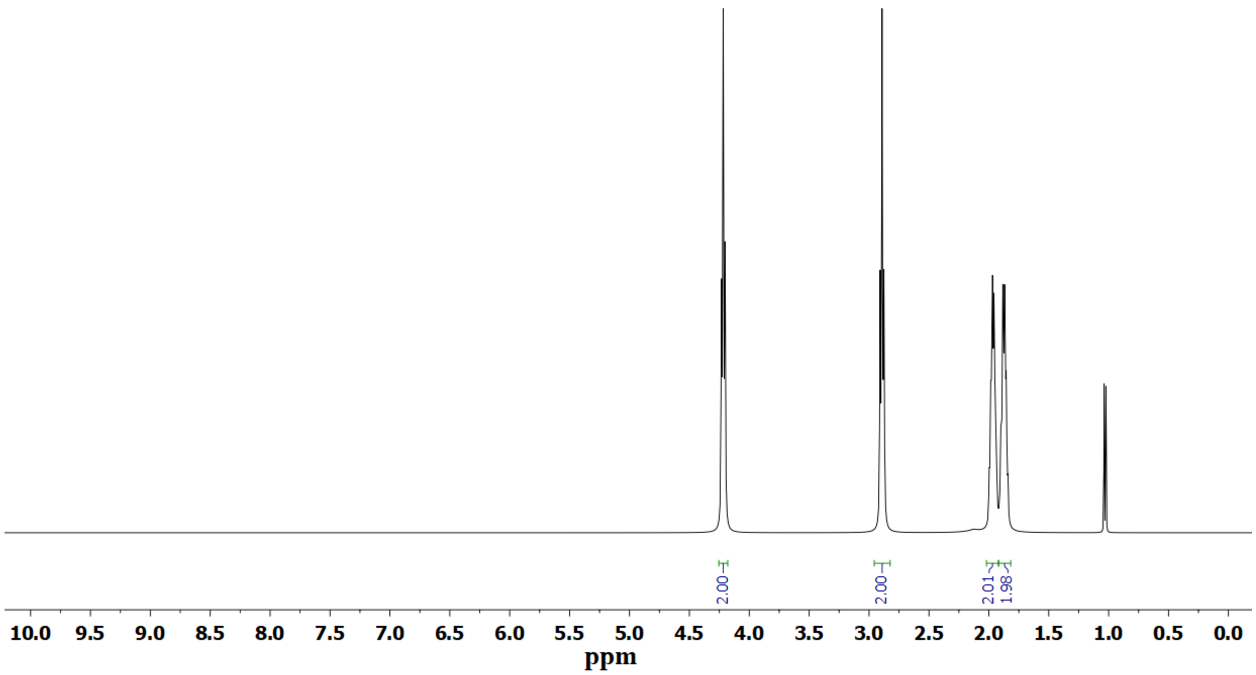

^{1}H NMR spectra of 7-bromo-8-chloro-1,2,3,4-tetrahydropyrido[1,2-*a*]benzimidazole-6,9-dione **(6b)** (DMSO-*d*6)

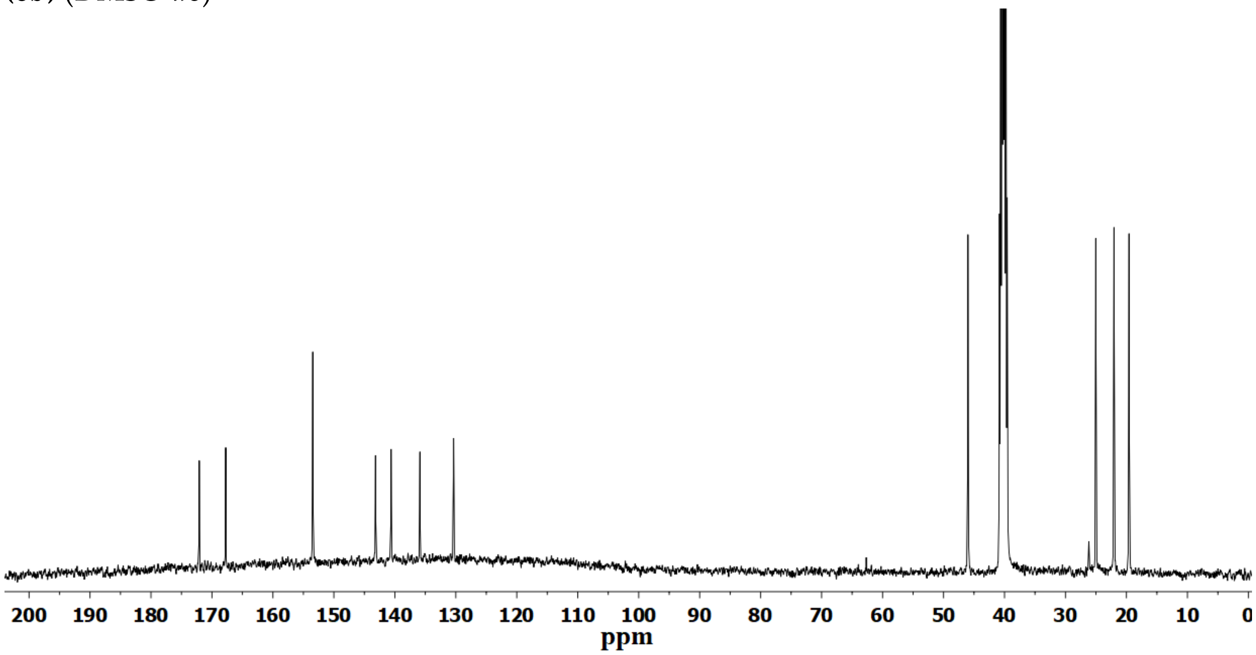

^{13}C NMR spectra of 7-bromo-8-chloro-1,2,3,4-tetrahydropyrido[1,2-*a*]benzimidazole-6,9-dione **(6b)** (DMSO-*d*6)

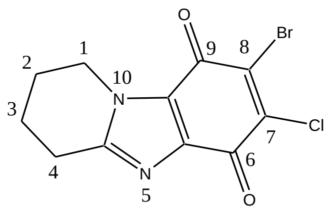

6c

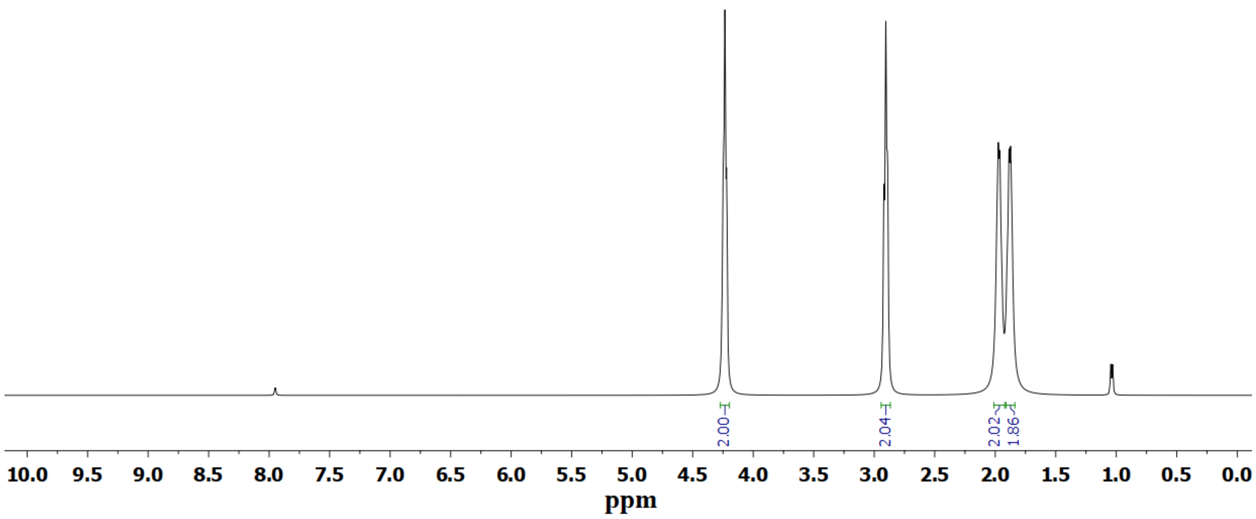

^{1}H NMR spectra of 8-bromo-7-chloro-1,2,3,4-tetrahydropyrido[1,2-*a*]benzimidazole-6,9-dione **(6c)** (DMSO-*d*6)

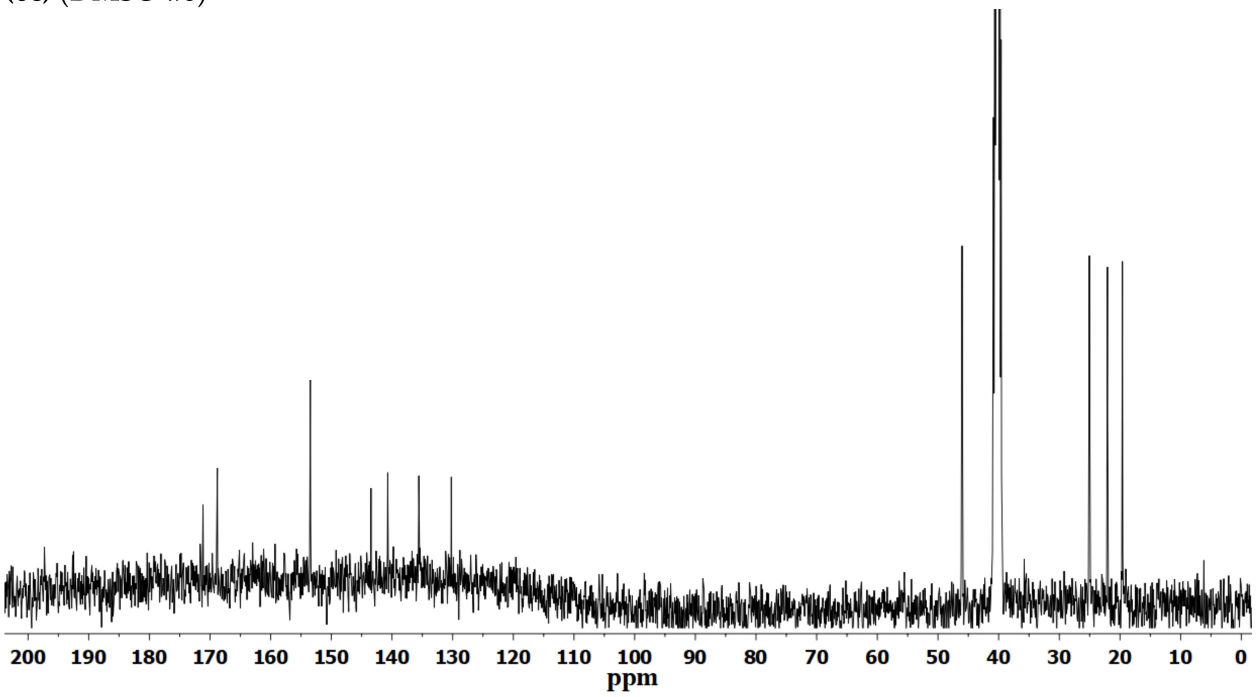

^{13}C NMR spectra of 8-bromo-7-chloro-1,2,3,4-tetrahydropyrido[1,2-*a*]benzimidazole-6,9-dione **(6c)** (DMSO-*d*6)

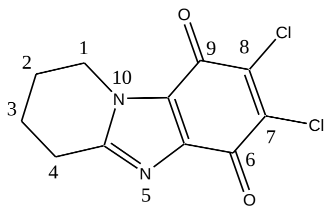

6d

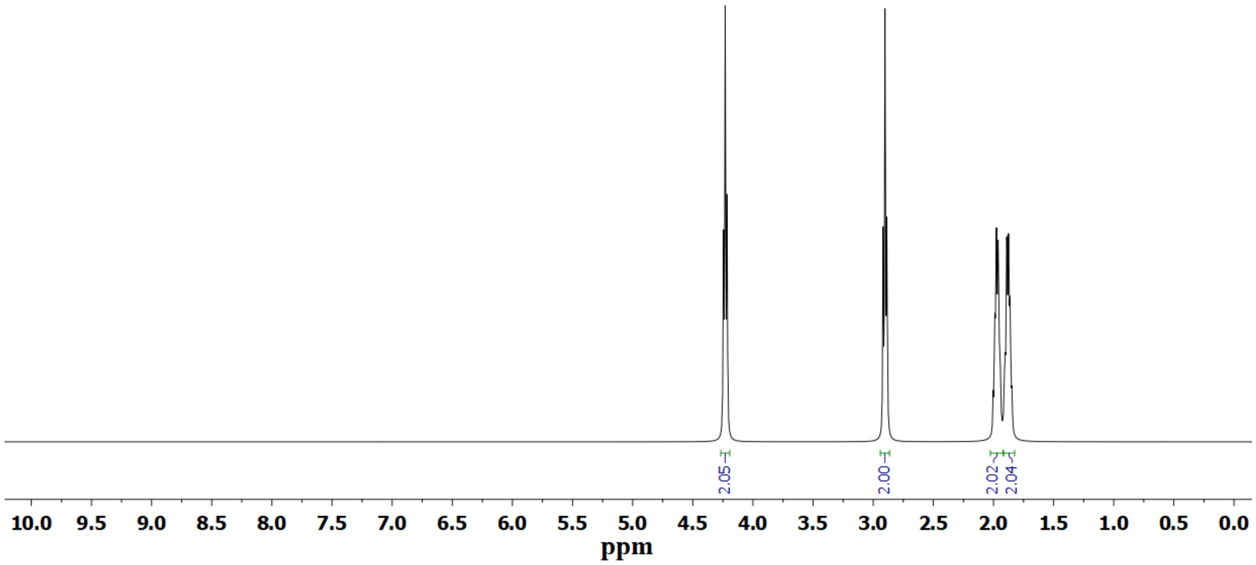

^{1}H NMR spectra of 7,8-dichloro-1,2,3,4-tetrahydropyrido[1,2-*a*]benzimidazole-6,9-dione **(6d)** (DMSO-*d*6)

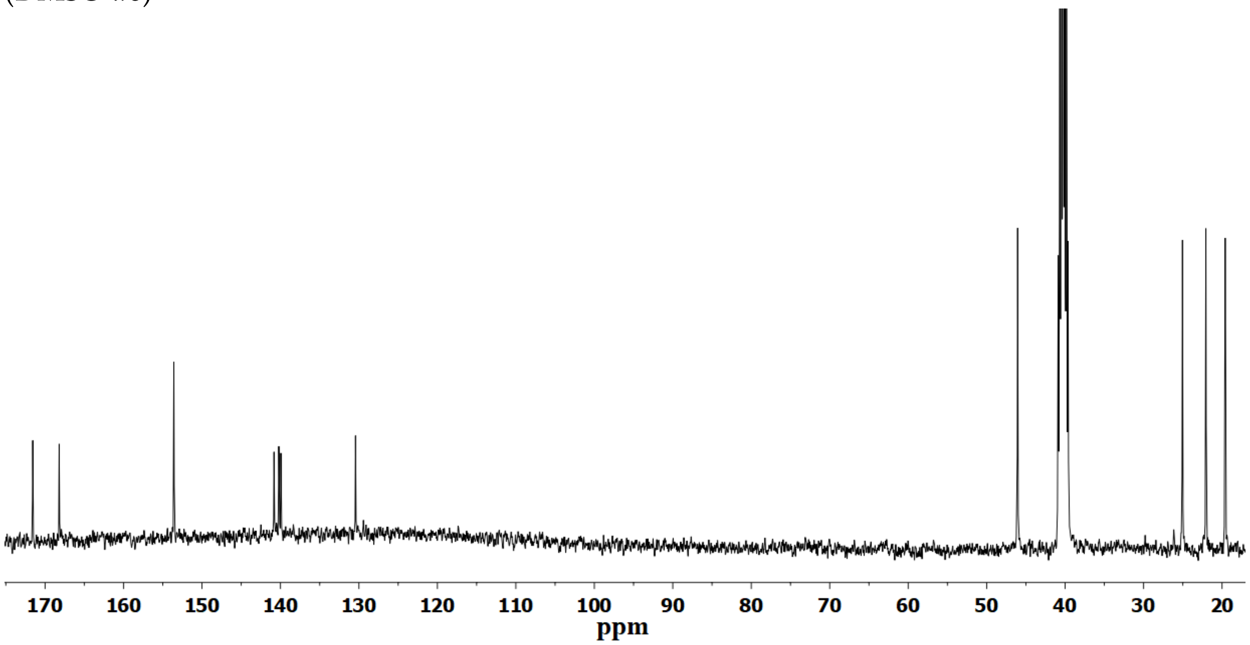

^{13}C NMR spectra of 7,8-dichloro-1,2,3,4-tetrahydropyrido[1,2-*a*]benzimidazole-6,9-dione **(6d)** (DMSO-*d*6)

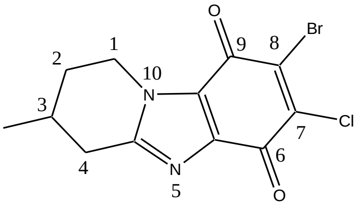

6e

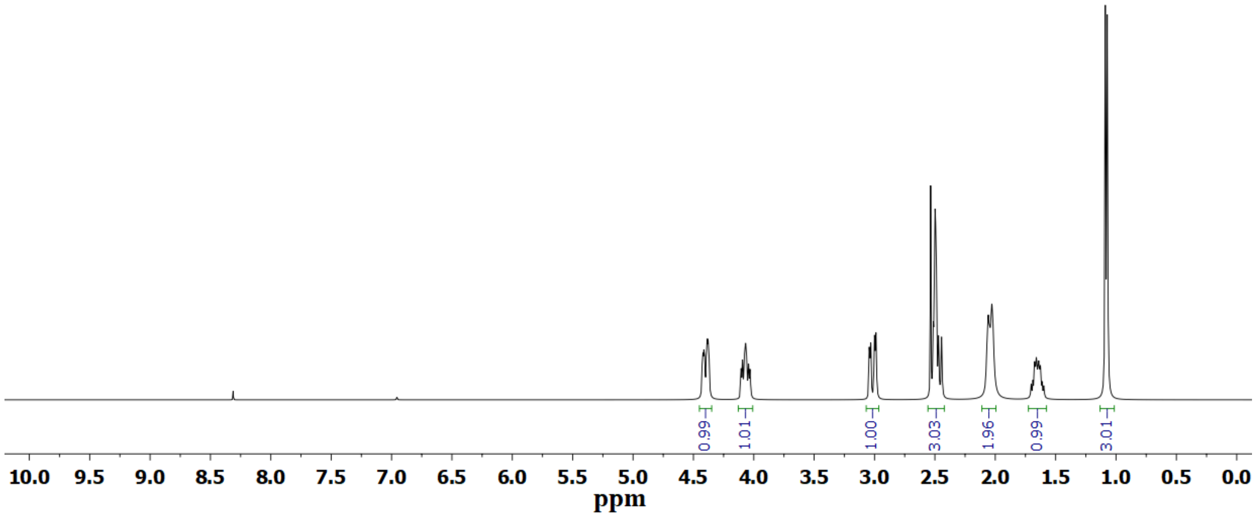

^{1}H NMR spectra of 8-bromo-7-chloro-3-methyl-1,2,3,4-tetrahydropyrido[1,2-*a*]benzimidazole-6,9-dione **(6e)** (DMSO-*d*6)

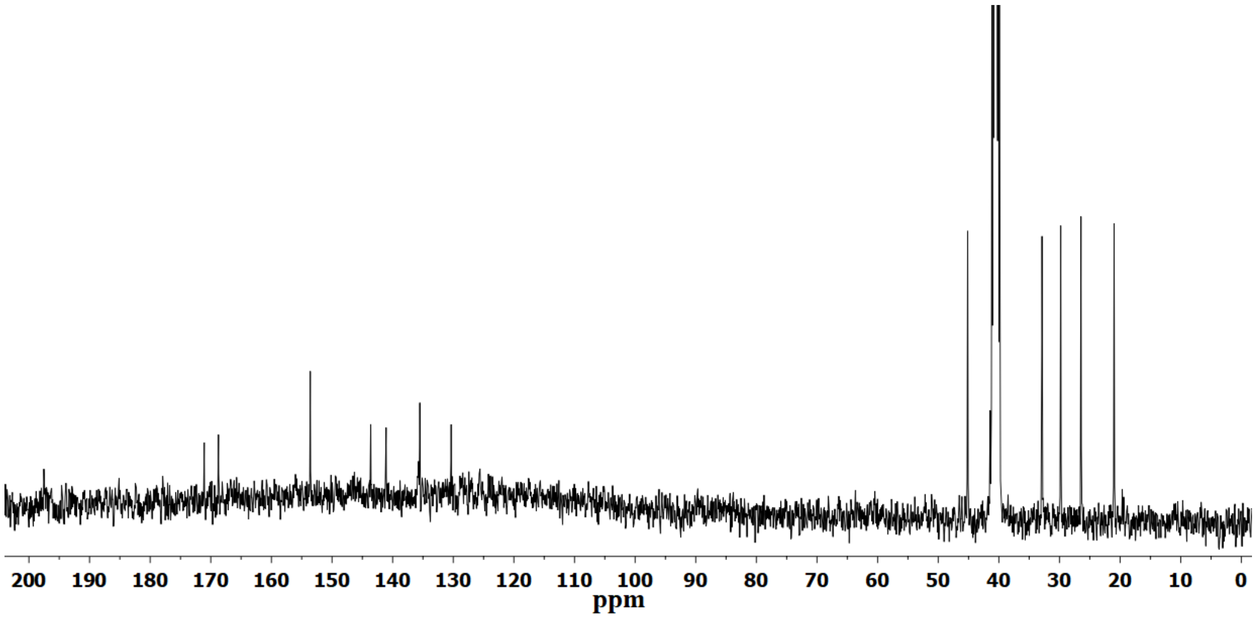

^{13}C NMR spectra of 8-bromo-7-chloro-3-methyl-1,2,3,4-tetrahydropyrido[1,2-*a*]benzimidazole-6,9-dione **(6e)** (DMSO-*d*6)

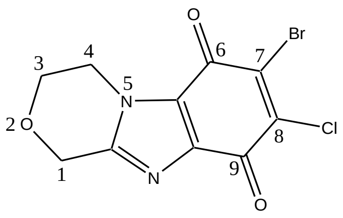

6f

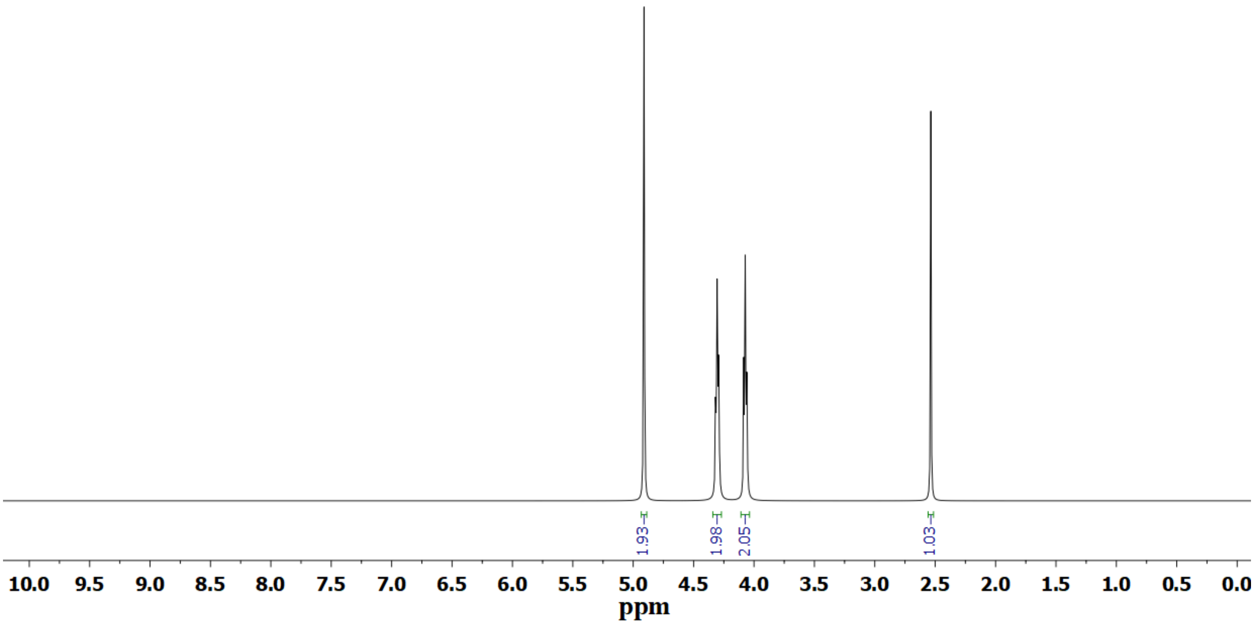

^{1}H NMR spectra of 7-bromo-8-chloro-3,4-dihydro-1*H*-[1,4]oxazino[4,3-*a*]benzimidazole-6,9-dione **(6f)** (DMSO-*d*6)

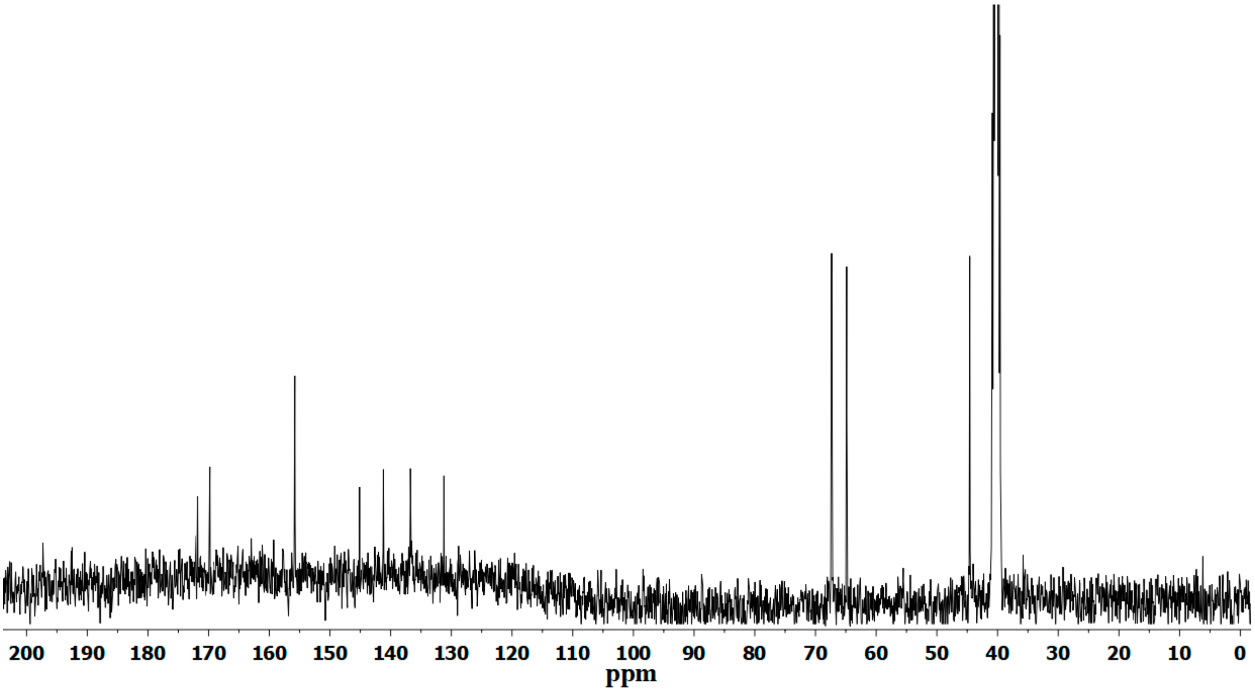

^{13}C NMR spectra of 7-bromo-8-chloro-3,4-dihydro-1*H*-[1,4]oxazino[4,3-*a*]benzimidazole-6,9-dione **(6f)** (DMSO-*d*6)

P_a[1]	P_i[2]	Cell line	Description
		**6a**	
0.705	0.009	DU-145	Prostate carcinoma
0.644	0.009	UACC-257	Melanoma
0.643	0.009	NCI-H322M	Non-small cell lung carcinoma
0.637	0.009	Malme-3M	Melanoma
0.607	0.013	OVCAR-5	Ovarian adenocarcinoma
0.566	0.014	A498	Renal carcinoma
0.557	0.014	HCC 2998	Colon adenocarcinoma
0.543	0.016	HCT-15	Colon adenocarcinoma
0.525	0.016	UO-31	Renal carcinoma
0.526	0.018	NCI-H226	Non-small cell lung carcinoma
		**6b**	
0.686	0.009	DU-145	Prostate carcinoma
0.641	0.009	UACC-257	Melanoma
0.628	0.010	Malme-3M	Melanoma
0.620	0.011	NCI-H322M	Non-small cell lung carcinoma
0.585	0.014	OVCAR-5	Ovarian adenocarcinoma
0.555	0.016	A498	Renal carcinoma
0.546	0.016	HCC 2998	Colon adenocarcinoma
0.527	0.016	UO-31	Renal carcinoma
0.519	0.019	NCI-H226	Non-small cell lung carcinoma
0.510	0.020	HCT-15	Colon adenocarcinoma
		**6c**	
0.686	0.009	DU-145	Prostate carcinoma
0.641	0.009	UACC-257	Melanoma
0.628	0.010	Malme-3M	Melanoma
0.620	0.011	NCI-H322M	Non-small cell lung carcinoma
0.585	0.014	OVCAR-5	Ovarian adenocarcinoma
0.555	0.016	A498	Renal carcinoma
0.546	0.016	HCC 2998	Colon adenocarcinoma
0.527	0.016	UO-31	Renal carcinoma
0.519	0.019	NCI-H226	Non-small cell lung carcinoma
0.510	0.020	HCT-15	Colon adenocarcinoma
		**6d**	
0.726	0.008	DU-145	Prostate carcinoma
0.713	0.008	OVCAR-5	Ovarian adenocarcinoma
0.691	0.007	NCI-H322M	Non-small cell lung carcinoma
0.689	0.006	UACC-257	Melanoma
0.668	0.007	Malme-3M	Melanoma
0.631	0.012	NCI-H226	Non-small cell lung carcinoma
0.613	0.009	UO-31	Renal carcinoma
0.606	0.010	A498	Renal carcinoma
0.604	0.010	HCC 2998	Colon adenocarcinoma
0.594	0.012	HCT-15	Colon adenocarcinoma
0.607	0.038	A549	Lung carcinoma
0.560	0.010	SNB-75	Glioblastoma
0.546	0.013	U-251	Glioma
0.543	0.012	BT-549	Breast ductal carcinoma
0.537	0.013	M14	Melanoma
0.534	0.013	NCI-H522	Non-small cell lung carcinoma

0.517	0.016	SR	Adult immunoblastic lymphoma
		**6e**	
0.486	0.022	DU-145	Prostate carcinoma
0.448	0.036	UACC-257	Melanoma
0.428	0.082	A549	Lung carcinoma
0.384	0.041	HCT-15	Colon adenocarcinoma
0.381	0.050	OVCAR-5	Ovarian adenocarcinoma
0.378	0.042	SK-MEL-2	Melanoma
0.377	0.043	Malme-3M	Melanoma
0.377	0.052	NCI-H322M	Non-small cell lung carcinoma
0.370	0.043	UO-31	Renal carcinoma
0.367	0.146	Hs 683	Oligodendroglioma
0.352	0.044	U-251	Glioma
0.335	0.051	HCC 2998	Colon adenocarcinoma
0.331	0.064	SNB-75	Glioblastoma
0.322	0.068	A498	Renal carcinoma
0.311	0.079	NCI-H226	Non-small cell lung carcinoma
0.311	0.018	HUVEC	Umbilical vein endothelial cell
		**6f**	
0.424	0.033	DU-145	Prostate carcinoma
0.395	0.121	Hs 683	Oligodendroglioma
0.379	0.054	K562	Erythroleukemia
0.364	0.047	SK-MEL-2	Melanoma
0.349	0.186	SK-MEL-1	Metastatic melanoma
0.336	0.112	A549	Lung carcinoma
0.317	0.082	PC-9	Lung adenocarcinoma
		**Mytomycin C**	
0.986	0.003	HeLa	Cervical adenocarcinoma
0.958	0.003	PBMC	Peripheral blood mononuclear cell
0.838	0.003	NCI-H838	Non-small cell lung cancer
0.714	0.004	DMS-114	Lung carcinoma
0.666	0.003	MDA-MB-453	Breast adenocarcinoma
0.658	0.002	A2780cisR	Cisplatin-resistant ovarian carcinoma
0.657	0.004	NCI-H187	Small cell lung carcinoma
0.653	0.003	SK-MEL-1	Metastatic melanoma
0.630	0.004	A2058	Melanoma
0.580	0.004	U-266	Plasma cell myeloma
0.559	0.010	RPMI-8226	Multiple myeloma
0.553	0.003	YAPC	Pancreatic carcinoma
0.536	0.004	SK-MES-1	Squamous cell lung carcinoma
0.529	0.004	HuP-T3	Pancreatic adenocarcinoma
0.522	0.021	UACC-257	Melanoma
0.520	0.005	PA-1	Ovarian carcinoma
0.519	0.005	RKO	Colon carcinoma
0.515	0.019	NCI-H226	Non-small cell lung carcinoma
0.503	0.022	NCI-H322M	Non-small cell lung carcinoma
0.502	0.005	AGS	Gastric adenocarcinoma

[1] Probability “to be active”.

[2] Probability “to be inactive”.
